# Harnessing Photo‐Energy Conversion in Nanomaterials for Precision Theranostics

**DOI:** 10.1002/adma.202501623

**Published:** 2025-05-16

**Authors:** Jingyu Shi, Yadi Fan, Qin Zhang, Yingying Huang, Mo Yang

**Affiliations:** ^1^ Department of Biomedical Engineering The Hong Kong Polytechnic University Kowloon Hong Kong 999077 China; ^2^ The Hong Kong Polytechnic University Shenzhen Research Institute Shenzhen 518000 China; ^3^ Joint Research Center of Biosensing and Precision Theranostics The Hong Kong Polytechnic University Kowloon Hong Kong 999077 China; ^4^ Research Center for Nanoscience and Nanotechnology The Hong Kong Polytechnic University Kowloon Hong Kong 999077 China

**Keywords:** bioimaging, multimodality therapy, nanomaterials, photo‐energy conversion, precision theranostics

## Abstract

The rapidly advancing field of theranostics aims to integrate therapeutic and diagnostic functionalities into a single platform for precision medicine, enabling the simultaneous treatment and monitoring of diseases. Photo‐energy conversion‐based nanomaterials have emerged as a versatile platform that utilizes the unique properties of light to activate theranostics with high spatial and temporal precision. This review provides a comprehensive overview of recent developments in photo‐energy conversion using nanomaterials, highlighting their applications in disease theranostics. The discussion begins by exploring the fundamental principles of photo‐energy conversion in nanomaterials, including the types of materials used and various light‐triggered mechanisms, such as photoluminescence, photothermal, photoelectric, photoacoustic, photo‐triggered SERS, and photodynamic processes. Following this, the review delves into the broad spectrum of applications of photo‐energy conversion in nanomaterials, emphasizing their role in the diagnosis and treatment of major diseases, including cancer, neurodegenerative disorders, retinal degeneration, and osteoarthritis. Finally, the challenges and opportunities of photo‐energy conversion‐based technologies for precision theranostics are discussed, aiming to advance personalized medicine.

## Graphical abstract

Harnessing photo‐energy conversion in nanomaterials enables precision theranostics through light‐driven mechanisms such as photoluminescence, photothermal, photoelectric, photoacoustic, photo‐triggered surface‐enhanced Raman scattering (SERS), and photodynamic processes. This review explores six fundamental principles of photo‐energy conversion, recent advances in photo‐triggered nanomaterials, and their diagnostic and therapeutic applications, with emphasis on their potential to address cancer, neurodegenerative diseases, retinal degeneration, and osteoarthritis.

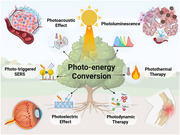

## 1 Introduction

Energy conservation is a fundamental principle stating that energy can neither be created nor destroyed, but can only be converted between different forms. This important concept lays the foundation for groundbreaking therapeutic strategies, particularly in the field of nanomedicine.^[^ ^1^ ^]^ Here, the ability to manipulate and convert various energy forms (e.g., photon, thermal, electrical, chemical, and mechanical energy) at the cellular and molecular levels opens the door to innovative healthcare solutions. Advanced nanomaterials and smart delivery systems are powerful platforms for orchestrating these energy conversion processes, enabling targeted energy conversion tailored to specific biomedical applications.^[^ ^2^, ^3^ ^]^ However, one of the most pressing challenges in energy‐converting nanomedicine is achieving precise control of therapeutic actions at disease sites while minimizing the impact on healthy tissues.

To address this challenge, stimuli‐responsive multifunctional nanosystems have been developed to convert external energy (such as ultrasound,^[^ ^4^, ^5^, ^6^, ^7^ ^]^ magnetic fields,^[^ ^8^, ^9^, ^10^, ^11^ ^]^ and light)^[^ ^12^, ^13^, ^14^ ^]^ into localized theranostic effects with remote controllability. Among these approaches, light (photo)‐triggered nanosystems are particularly promising due to their unique advantages. Light‐triggered nanosystems offer sub‐millimeter spatial precision, which surpasses the centimeter‐scale dispersion associated with other energy sources like ultrasound or magnetic fields.^[^ ^15^, ^16^ ^]^ Additionally, light provides multispectral programmability, allowing wavelength‐specific activation of multiple functions, as well as non‐contact operability.^[^ ^13^, ^17^ ^]^ Unlike conventional therapeutic approaches (e.g., chemotherapy, radiotherapy, systemic drug delivery), photo‐triggered systems enable treatment at target sites with on‐demand activation and deactivation, significantly reducing off‐target effects.^[^ ^18^, ^19^ ^]^ Furthermore, their ability to integrate diagnostic and therapeutic functions into a single platform represents a major advancement over traditional diagnostic tools (e.g., MRI, CT, PET) and standalone therapeutic modalities.^[^ ^20^, ^21^, ^22^ ^]^ The importance of photo‐triggered nanosystems is further enhanced by the use of near‐infrared (NIR) light (700–1700 nm), which is especially valuable for in vivo applications due to its deeper tissue penetration (up to several centimeters) and minimal interference with biological tissues.^[^ ^23^ ^]^

**Figure 1:**
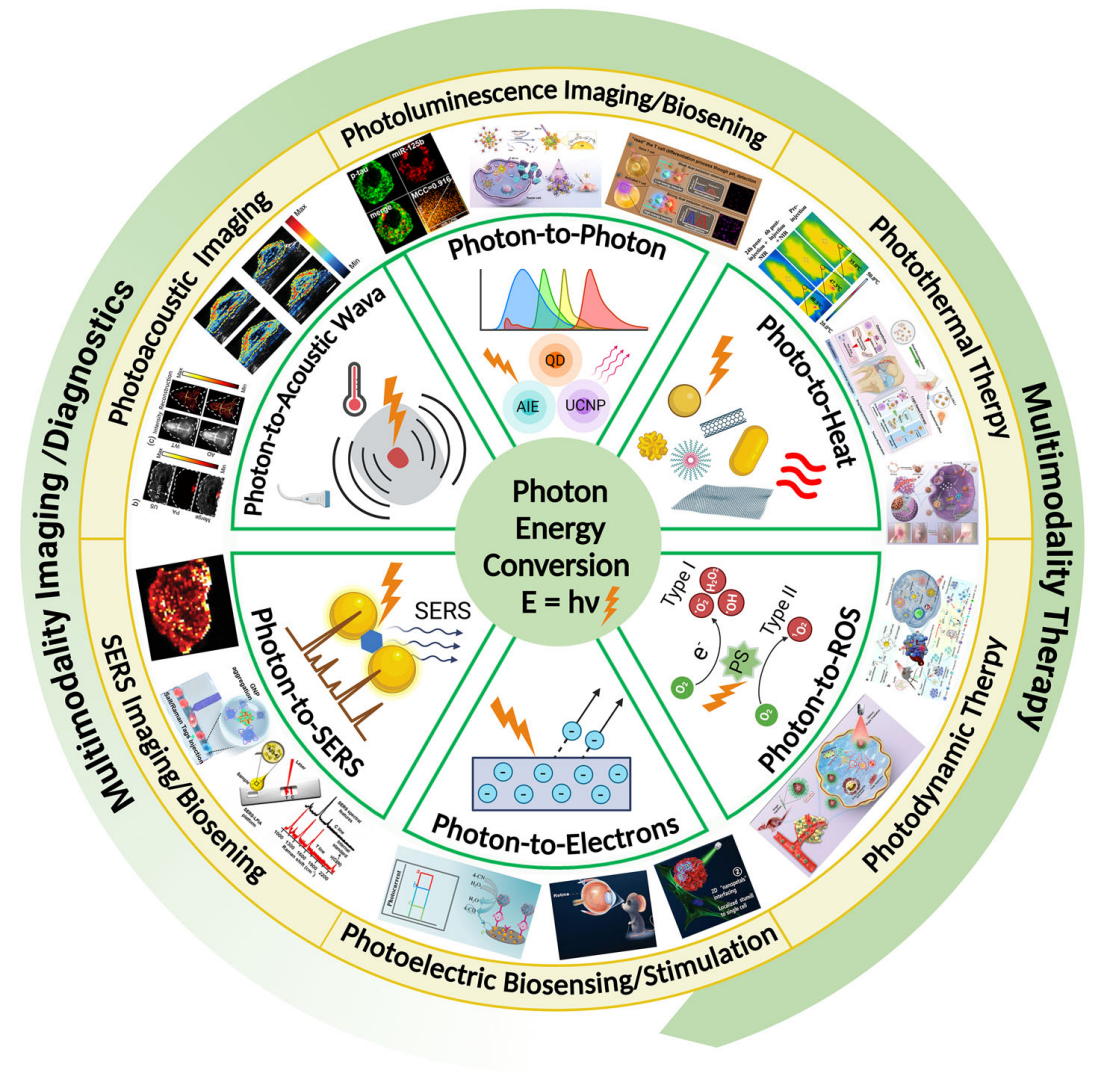
Overview of mechanisms, categories, and applications of photo‐energy conversion‐based nanomaterials. Created with BioRender.com. Composite image created using figures reproduced with permission.^[^ ^90^, ^226^, ^300^ ^]^ Copyright 2024;^[^ ^403^ ^]^Copyright 2021, American Chemical Society;^[^ ^91^, ^235^, ^326^ ^]^ Copyright 2023;^[^ ^406^ ^]^ Copyright 2024 Elsevier;^[^ ^107^, ^295^, ^297^ ^]^ Copyright 2024;^[^ ^34^, ^259^ ^]^ Copyright 2023, Wiley‐VCH;^[^ ^32^ ^]^ Copyright 2024, Oudeng et al., published by Wiley‐VCH;^[^ ^350^ ^]^ Copyright 2024, Liao et al., published by Elsevier.

Photo‐energy conversion‐based nanomaterials exhibit remarkable capabilities in converting absorbed photon energy into various forms through distinct photophysical and photochemical processes, enabling a wide range of biosensing, bioimaging, and theranostic applications (**Figure** **1**). Briefly, photoluminescence (PL) involves the re‐emission of photons at different wavelengths via radiative relaxation, enabling luminescent sensing and imaging of biological processes.^[^ ^24^, ^25^ ^]^ Photothermal effect occurs when absorbed photon energy transforms into heat through plasmonic localized heating or non‐radiative relaxation pathways, providing the basis for photothermal therapy (PTT) and thermal‐triggered drug release.^[^ ^2^, ^26^, ^27^, ^28^ ^]^ Photoelectric effect generates electron‐hole pairs that can be separated to produce photocurrent, finding applications in photoelectrochemical (PEC) biosensing and cellular photostimulation.^[^ ^29^, ^30^, ^31^, ^32^ ^]^ Photo‐triggered surface‐enhanced Raman scattering (SERS) utilizes photo‐excited plasmons in metallic nanostructures to amplify localized electromagnetic fields, making it capable of ultrasensitive molecular detection and imaging.^[^ ^33^, ^34^, ^35^, ^36^ ^]^ Photoacoustic (PA) effect, resulting from rapid thermal expansion and subsequent generation of acoustic waves upon light absorption, is particularly advantageous for deep‐tissue photoacoustic imaging (PAI) with high spatial resolution.^[^ ^37^, ^38^, ^39^, ^40^ ^]^ Lastly, photodynamic effect involves light‐activated photosensitizers that generate cytotoxic reactive oxygen species (ROS) for photodynamic therapy (PDT).^[^ ^41^, ^42^, ^43^ ^]^ These diverse photo‐triggered energy conversion processes often occur simultaneously or sequentially, offering several key advantages in theranostic applications. First, they enable precise spatiotemporal control over therapeutic actions through non‐invasive light activation, allowing physicians to selectively treat diseased tissues while minimizing side effects on healthy tissues. Second, the versatility of photo‐triggered responses allows the integration of sensing, imaging, and therapy within a single platform, providing a multifunctional approach to disease management. This integration is achieved by combining several key functions into one cohesive system. The targeting function can be achieved either actively or passively, ensuring the nanomaterials specifically localize to diseased tissues or cells, enhancing the precision of both diagnosis and therapy. The imaging function, often facilitated by fluorescence imaging (FLI), SERS imaging, or PAI, provides real‐time visualization of the target area, aiding in accurate diagnosis and monitoring of treatment progress. The passivation function involves surface modification with biocompatible materials (such as PEG, zwitterionic polymers, or cell membranes) to improve biocompatibility and stability and enhance circulation time in the body. The diagnostic function utilizes photo‐triggered mechanisms (such as PL, SERS, PA, and PEC) to detect molecular biomarkers with high sensitivity, enabling early and accurate disease detection. Finally, the therapy function utilizes effects such as PTT and PDT to deliver targeted treatment, such as heat‐induced tumor ablation and ROS generation for cell destruction. By integrating these functions, photo‐triggered platforms offer a comprehensive solution that not only improves the efficacy and specificity of disease management but also streamlines the clinical workflow, ultimately leading to better patient outcomes.

In this review, we aim to present a comprehensive overview of the state‐of‐the‐art advancements in photo‐energy conversion using nanomaterials for diagnosis, multimodal imaging, and therapy. We will first discuss the basic principles of light energy conversion processes, including PL, photothermal, photoelectronic, photoacoustic, and photo‐triggered SERS, and photodynamic effects, and summarize the main categories of nanomaterials according to different conversion mechanisms. We will then explore the current applications of precision theranostics based on light energy conversion in various diseases such as cancer, neurodegenerative diseases, retinal degeneration, and osteoarthritis. Finally, we will provide our perspectives on the current clinical status and challenges as well as future directions in the field of photo‐triggered nanomedicine. In contrast to our earlier review papers,^[^ ^44^, ^45^, ^46^, ^47^, ^48^ ^]^ this work prioritizes elucidating the fundamental photo‐energy conversion mechanisms and their broad applications in precision theranostics, shifting focus away from material‐centric developments or application‐specific studies. This comprehensive review will provide researchers with a thorough understanding of the current status of photo‐energy conversion in theranostics, serving as a valuable resource for future developments in this rapidly evolving field.

## 2 Fundamental Principles of Photo‐Energy Conversion in Nanomaterials

Photo‐responsive energy conversion represents the cornerstone of photo‐triggered nanomaterials in theranostics. When nanomaterials absorb photons, they initiate various conversion pathways that generate therapeutic and diagnostic effects. The effectiveness of these conversions fundamentally depends on the incident photon energy (*E = hν*), which must meet specific energetic thresholds to activate different processes. Higher energy UV photons (3.1–6.2 eV; 200–400 nm) can directly trigger electronic transitions and photochemical reactions but face limited tissue penetration due to strong absorption and scattering by biological chromophores.^[^ ^49^ ^]^ Visible photons (1.8–3.1 eV; 400–700 nm) provide sufficient energy for many photosensitizer excitations and plasmonic effects while offering moderate tissue penetration.^[^ ^50^ ^]^ NIR photons (NIR‐I: 700–950 nm; NIR‐II: 1000–1700 nm) possesses lower energy (0.7–1.8 eV) but enables deeper tissue penetration, making it ideal for treating deep‐seated conditions.^[^ ^51^ ^]^

**Figure 2:**
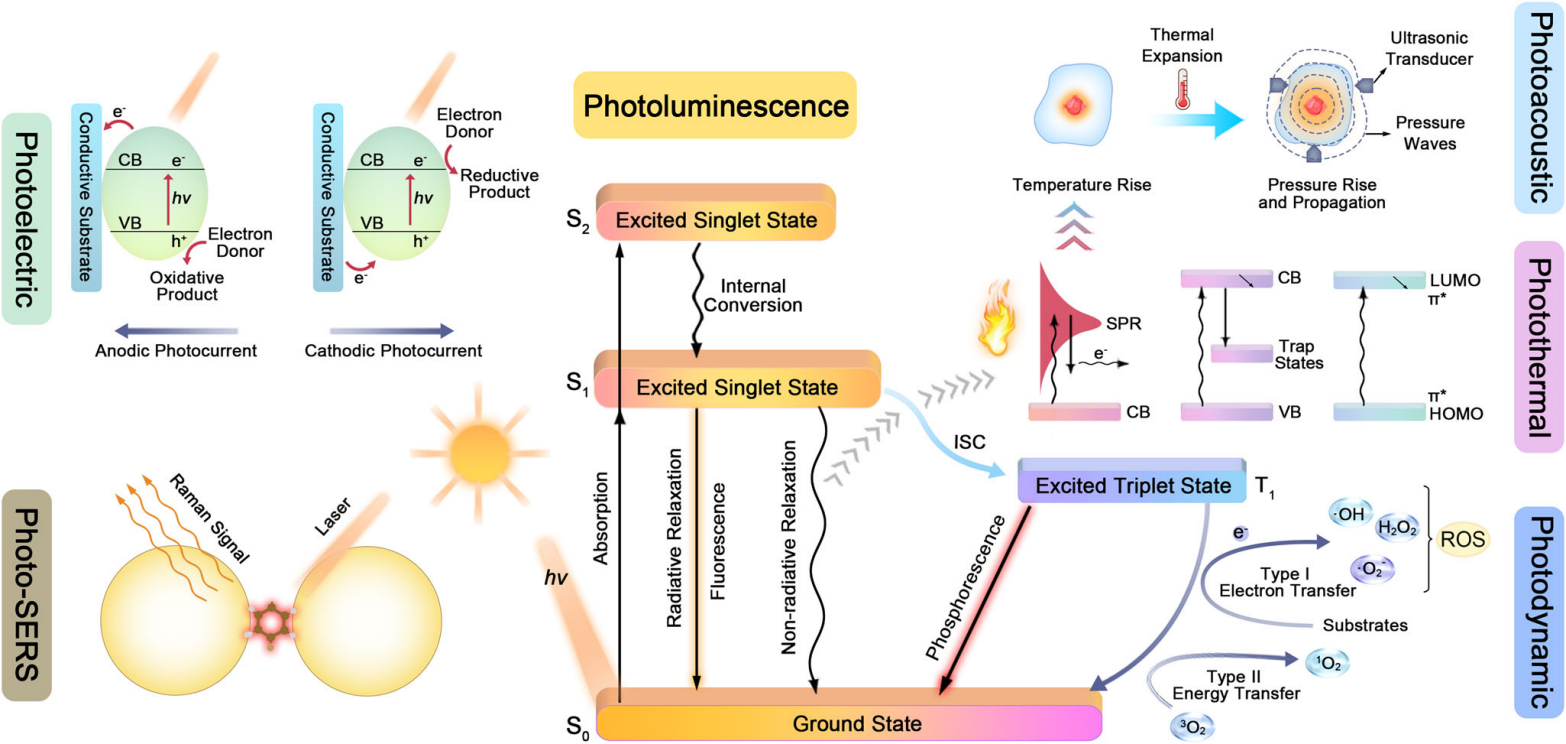
Energy diagrams of various photo‐energy conversion phenomena.

Following the Jablonski diagram, photo‐triggered energy conversion processes begin with photon absorption, where incident photons excite electrons from their ground electronic state (*S₀*) to higher‐energy excited singlet states (*S₁ or S_2_*). This photon absorption step can be quantitatively described by the Beer–Lambert law:

(1) $$I=I_{0}e^{\left(-\varepsilon cl\right)}$$

where I and I_0_ respectively represent the light radiation intensity after and before the absorption, 𝓔 is the extinction coefficient, c is the concentration of particles, and l is the depth or optical path length. Electrons in these excited states subsequently undergo various relaxation pathways, categorized as distinct primary and secondary conversion processes, each characterized by unique mechanisms and occurring over different temporal and spatial scales (**Figure** **2** and **Table** **1**). The primary processes occur rapidly at nanoscale dimensions. For example, photon‐to‐photon conversion involves radiative relaxation back to the ground state (*S_1_* to *S_0_*), emitting photons of different wavelengths via fluorescence (*S₁* to *S₀*, nanoseconds (10^−9^ s)) or phosphorescence (*T₁* to *S₀*, milliseconds to seconds (10^−3^–1 s)) at molecular scales (0.1–10 nm).^[^ ^52^ ^]^ The photothermal effect involves the conversion of photon engergy into heat through nonradiative relaxation or localized heating of plasmons, generating thermal energy in picoseconds to nanoseconds (10^−12^–10^−9^ s) that are initially confined to nanoscale dimensions of <100 nm before diffusing thermally to micrometer (µm) scales.^[^ ^53^ ^]^ The photoelectric effect generates charge carriers through photon‐induced bandgap transitions or electron ejection, occurring on femtosecond (10^−15^ s) timescales and spanning atomic to nanometer dimensions (0.1–10 nm).^[^ ^29^ ^]^ In contrast, secondary conversion processes typically occur over longer timescales and broader spatial dimensions, often requiring specific environmentalinteractions. For instance, the PA effect transforms photo‐induced thermal energy into mechanical waves through thermoelastic expansion of the surrounding medium, propagating on microsecond timescales and extending from microscale to millimeter scales (1 µm–10 mm).^[^ ^54^ ^]^ Photo‐stimulated SERS involves the amplification of local electromagnetic fields through photo‐excited plasmons, enhancing Raman scattering from nearby molecules within near‐field regions (1–50 nm) of the plasmonic surface.^[^ ^55^ ^]^ Finally, the photodynamic effect generates ROS through a cascade process involving photosensitizer excitation, intersystem crossing (ISC), and energy transfer to molecular oxygen, occurring on microsecond to millisecond (10^−6^–10^−3^ s) timescales and diffusing across cellular dimensions.^[^ ^56^ ^]^

**Table 1:** Comparison of various photo‐energy conversion phenomena.

Photo‐energy conversion effects	Energy conversion	Mechanisms	Timescales	Spatial scales	Applications	Refs.
Photoluminescence	Photon‐to‐photon	Radiative relaxation Downconversion luminescence: emission *S_1_* to *S_0_* Upconversion luminescence: ESA, ETU, PA Phosphorescence: emission *T_1_* to *S_0_*	Downconversion: 10^−9^ s; Upconversion: 10^−6^–10^−3^ s; Phosphorescence: 10^−3^–1 s	0.1‐10 nm	Biosensing, Bioimaging	[52, 95, 96, 109]
Photothermal effect	Photon‐to‐heat	Plasmonic localized heating Non‐radiative relaxation in semiconductors Thermal vibrations of molecules	Photon absorption: 10^−15^–10^−13^ s; Electron‐phonon coupling: ≈10^−12^ s; Thermal diffusion: 10^−10^–10^−8^ s	Initial heating: < 100 nm; Thermal diffusion: µm	Therapy, Bioimaging	[53, 120, 121, 122, 123, 124, 125]
Photoelectric effect	Photon‐to‐electrons	Electron‐hole pairs generation in semiconductors Free electrons ejection in metals Interfacial charge separation and transfer in hybrid nanostructures	Photon absorption: 10^−15^ s; Carrier generation: 10^−12^ s; Current collection: 10^−9^ s	Charge generation: 0.1‐10 nm; Current collection: µm	Biosensing, Therapy	[29, 135, 136]
Photoacoustic effect	Photon‐to‐ heat‐to‐ acoustic wave	Photon absorption generates localized heating, causing rapid thermal expansion and acoustic wave propagation detectable as ultrasound signals	Light absorption: 10^−12^ s; Thermal expansion: 10^−9^ s; Wave propagation: 10^−6^ s	Initial absorption: nm; Wave propagation: 1 µm‐10 mm	Bioimaging	[54, 150, 151]
Photo‐triggered SERS effect	Photon‐to‐vibration energy	Photon excitation of localized surface plasmons creates highly intensified electromagnetic fields, significantly enhancing Raman scattering signals from nearby molecules	Plasmon excitation: 10^−15^ s; Raman scattering: 10^−12^ s	< 10 nm (hot spots)	Biosensing, Bioimaging	[55, 163]
Photodynamic effect	Photon‐to‐ ROS	Photosensitizer absorbs photons and undergoes ISC to *T₁*, subsequently transferring energy to molecular O₂ to generate ROS, causing oxidative damage to biological targets	Initial excitation: 10^−9^ s; ISC: 10^−6^ s; Energy transfer (ROS generation): 10^−3^ s	Singlet oxygen (¹O₂): 10–20 nm; other ROS (e.g., •OH, H₂O₂, O_2_^•−^): 100 nm‐100 µm	Therapy	[56, 173, 174, 175, 176]

By carefully considering the temporal and spatial characteristics of each photo‐energy conversion process, along with the desired biological effects, more effective diagnostic and therapeutic platforms can be developed. The following sections will elaborate on each conversion mechanism and discuss the corresponding nanomaterials employed, providing insights for advancing the field of photo‐triggered nanomedicine.

### 2.1 Photon‐to‐Photon Conversion in Nanomaterials

Photon‐to‐photon conversion, or PL, occurs when absorbed photons trigger electronic transitions that ultimately result in photons emission at different wavelengths. This quantum mechanical process elevates electrons from their ground state (*S₀*) to excited singlet states (*S₁, S₂*), followed by distinct relaxation pathways: (a) down‐conversion luminescence, (b) phosphorescence, and (c) up‐conversion luminescence.

#### 2.1.1 Downconversion Luminescence in Nanomaterials

Down‐conversion luminescence, specifically fluorescence, represents the most direct radiative relaxation pathway. After rapid internal conversion from higher excited states to the first excited singlet state (*S₁*), electrons undergo spin‐allowed radiative relaxation to *S₀* on nanosecond timescales (10⁻⁹–10⁻⁸ s). The process is confined to molecular dimensions (0.1–1 nm) and results in the short‐lifetime emission of lower‐energy photons due to vibrational relaxation, manifesting as the characteristic Stokes shift. The quantum yield of fluorescence is determined by the competition between radiative and non‐radiative decay pathways, which can be modulated through material design and environmental conditions.

Among various fluorescent nanomaterials, quantum dots (QDs) have emerged as exceptional candidates for biomedical applications due to their pronounced quantum confinement and edge effect. Traditional semiconductor QDs, including ZnX, CdX, HgX, and PbX (where X = S, Se, Te), offer superior downconversion properties with a narrow fluorescence emission spectrum, high quantum yield, and remarkable photostability.^[^ ^57^ ^]^ However, concerns regarding their potential toxicity have driven the development of alternative QD systems. Graphene quantum dots (GQDs) represent a unique class of 0D nanomaterials characterized by an atomically thin layer with dimensions less than 10 nm.^[^ ^58^ ^]^ Unlike traditional semiconductor QDs containing heavy metals, GQDs exhibit superior biocompatibility and lower toxicity as carbon‐based material while maintaining excellent optical properties as QDs, such as tunable PL, high quantum yield, and excellent photostability.^[^ ^59^ ^]^ Their small size, ease of functionalization, excellent water dispersibility, and potential photothermal and photodynamic properties make GQDs promising for various biological applications, including biosensing,^[^ ^60^, ^61^, ^62^ ^]^ bioimaging,^[^ ^63^, ^64^ ^]^ and theranostics.^[^ ^65^, ^66^ ^]^ Carbon dots (CDs) with spherical morphology are typically larger than 3 nm.^[^ ^67^ ^]^ Their optical properties are primarily dictated by surface energy traps rather than the quantum confinement effects seen in GQDs. Recent advancements in CDs engineering highlight their advantages, such as simpler synthesis, better chelation stability, and superior multimodal imaging capabilities, making them valuable complements to GQDs in bioimaging and phototherapy.^[^ ^28^, ^68^, ^69^ ^]^ Nanoparticles with quantum confinement effects can also be derived from other graphene‐like 2D nanomaterials, including transition metal dichalcogenides (TMDs, e.g., MoS₂ QDs, WS₂ QDs), MXenes (e.g., Ti_3_C_2_ QDs), black phosphorus (BP QDs), and graphitic carbon nitride (g‐C_3_N_4_ QDs).^[^ ^70^, ^71^ ^]^ Compared to their 2D forms, these graphene‐like 2D nanomaterials‐derived QDs (2D QDs) have a larger surface area and a strong quantum confinement effect, which enable them with better dispersibility, easier functionalization, and more distinct PL characteristics. Similar to GQD, the PL properties of 2D QDs can be systematically tuned by size effect, surface defects, and heteroatom doping. For example, Yin et al. demonstrated highly luminescent WS₂ QDs with size‐tunable emission and an impressive quantum yield of 32%, highlighting their potential for optoelectronic applications.^[^ ^72^ ^]^ Zhu et al. demonstrated the versatility of surface chemistry in tuning optical properties by achieving tunable band gaps in MoS₂ QDs through controlled functionalization with hydroxyl and aldehyde groups.^[^ ^73^ ^]^ Xu et al. developed Ti_3_C_2_ QDs with tunable emission from blue to orange through S, N, and S/N doping, achieving quantum yields ranging from 7.78% to 28.12%.^[^ ^74^ ^]^

Recently, perovskite QDs with the ABX₃ configuration, where A represents Cs⁺, CH₃NH₂⁺ (MA⁺), or CH(NH₂)₂⁺ (FA⁺); B is a divalent metal cation (typically Pb^2^⁺); and X denotes halide ions (Cl⁻, Br⁻, I⁻), have attracted significant attention for their exceptional photoluminescent quantum yield, efficient charge carrier transport and minimized energy loss, driving transformative advancements in photonics and optoelectronics.^[^ ^75^, ^76^ ^]^ However, their stability and toxicity issues hinder their widespread application. Various methods have been developed to address these limitations. For example, Li et al. used a core/shell strategy to grow a lead‐free CsMnCl_3_ shell on the surface of CsPbCl_3_ to form CsPbCl_3_/CsMnCl_3_ core/shell nanocrystals, achieving enhanced environmental stability and improved PL quantum yield (28.5%).^[^ ^77^ ^]^ Li et al. reported Mn^2+^‐doped CsPbCl_3_/CsPb_2_Cl_5_ core/shell heteroperovskite nanocrystals and further embedded them into a hydrophilic agarose matrix to create a stable sensing platform for pesticide detection in food.^[^ ^78^ ^]^

Metal–organic frameworks (MOFs) are porous hybrid materials constructed from metal ions/clusters and polydentate organic ligands connected by coordination bonds.^[^ ^79^ ^]^ They exhibit remarkable structural flexibility, allowing reversible guest uptake and release, while their frameworks can be readily functionalized through pre‐installation or post‐modification strategies. Traditional single‐emission MOFs are often limited by narrow analyte sensitivity and susceptibility to environmental interference, which alter absolute emission intensity.^[^ ^80^, ^81^ ^]^ To overcome these issues, dual‐emission MOFs have been developed by incorporating two luminescent centers. Deng et al. encapsulated Rh6G dye within UIO‐66‐NH₂ MOF pores (Rh6G@UIO‐66‐NH_2_), displaying two broad emission bands centered at 378 and 495 nm.^[^ ^82^ ^]^ Zhao et al. anchored a Zr‐based MOF on a rare‐earth MOF (UiO‐66(OH)_2_@Y‐TCPP), yielding dual emissions at 496 and 676 nm.^[^ ^83^ ^]^ Zhang et al. incorporate gold nanoclusters (AuNCs) and GQDs onto the inner and outer surface of a zeolitic imidazolate MOF (AuNCs/GQDs@ZIF‐8), resulting in dual emission at 445 nm and 580 nm.^[^ ^84^ ^]^ Through these approaches, the PL properties of MOF can be tuned while providing ratiometric fluorescence sensing responses.

Recently, aggregation‐induced emission (AIE) processes have attracted considerable attention. AIE luminogens (AIEgens) exhibit strong fluorescence when aggregated but weak or no emission when dissolved, which is the opposite of the aggregation‐caused quenching (ACQ) effect observed in traditional fluorophores.^[^ ^85^ ^]^ Their distinctive emission mechanism relies on the restriction of intramolecular motions (RIM) in the aggregated state. When molecularly dissolved, AIEgens, typically featuring propeller‐shaped structures with rotatable groups, undergo active intramolecular rotations that serve as non‐radiative relaxation pathways, effectively quenching fluorescence. Upon aggregation, these molecular motions become restricted, blocking the non‐radiative pathways and enabling strong emission. The emission properties of AIE molecules can be systematically tuned through molecular structure design, including modifying π‐conjugation length,^[^ ^86^ ^]^ incorporating donor–acceptor (D‐A) groups,^[^ ^87^ ^]^ and engineering propeller‐shaped structures.^[^ ^88^ ^]^ For example, Xu et al. developed a high‐performance two‐photon NIR‐II AIEgen (MeTTh) by introducing a strong D‐A structure, in which methyl‐substituted diphenylamines acted as donors, thiophenes acted as π‐bridges, and rhodanines acted as acceptors.^[^ ^89^ ^]^ In addition, low cytotoxicity and excellent biocompatibility make AIEgens ideal for biomedical applications, spanning biosensing,^[^ ^44^, ^90^, ^91^, ^92^ ^]^ bioimaging,^[^ ^93^ ^]^ and theranostic platforms.^[^ ^94^ ^]^

#### 2.1.2 Upconversion Luminescence in Nanomaterials

Up‐conversion luminescence represents a distinct mechanism involving the sequential absorption of multiple lower‐energy photons to produce higher‐energy photon emission (anti‐Stokes shift).^[^ ^95^ ^]^ The most widely studied materials for upconversion are lanthanide‐doped nanoparticles, with systems such as NaYF_4_:Yb/Er, NaYF_4_:Yb/Tm, and NaGdF_4_:Yb/Er, where NaYF_4_ or NaGdF_4_ as crystalline host matrix, Yb^3+^ as a sensitizer and Er^3+^ or Tm^3+^ as activators.

The upconversion process occurs through three primary mechanisms, including excited‐state absorption (ESA), energy transfer upconversion (ETU), and photon avalanche upconversion.^[^ ^96^ ^]^ In ESA, a single ion (e.g., Er^3+^, Tm^3+^) undergoes sequential multi‐photon absorption to reach higher energy excited states from the ground state, representing the fundamental upconversion process. ETU as the most efficient mechanism in lanthanide‐doped systems involves energy coupling between two excited state ions with similar energies through non‐radiative processes. In this process, the sensitizer ion (typically Yb^3^⁺) absorbs NIR photons and sequentially transfers this energy to nearby activator ions (commonly Er^3^⁺ or Tm^3^⁺), ultimately leading to the population of higher excited states and subsequent radiative emission at shorter wavelengths. Photon avalanche upconversion combines excited state absorption and energy transfer, distinguished by energy transfer occurring exclusively between ions of the same type. The spatiotemporal dynamics of these processes are complex, spanning microseconds to milliseconds (10^−6^–10^−3^ s) with energy migration across 1–10 nm, typically confined within nanoparticles of 10–100 nm dimensions. The composition and structure of upconversion nanoparticles (UCNPs) significantly influence their efficiency. Fluoride‐based crystals, such as NaYF₄ and NaGdF₄, are the most effective hosts due to their low phonon energies, which reduce non‐radiative losses. Other host materials include oxides like Y₂O₃ and ZrO₂, and halides like YCl₃ and YBr₃. The selection of lanthanide dopants is also crucial, as tailored dopants allow UCNPs to emit light across a broad spectrum, from UV and visible (blue, green, red) to short NIR wavelengths.^[^ ^97^ ^]^ UCNPs have several unique optical properties, including long luminescence lifetimes (ranging from µs to ms), sharp emission bands, large anti‐Stokes shifts, high photostability (with no photobleaching or blinking), and minimal autofluorescence background. Excitable by NIR light, UCNPs enable deep tissue penetration while generating visible or UV emissions, making them highly suitable for biosensing,^[^ ^97^, ^98^, ^99^ ^]^ bioimaging,^[^ ^100^ ^]^ and therapy.^[^ ^101^, ^102^, ^103^ ^]^

#### 2.1.3 Förster Resonance Energy Transfer (FRET) Effect

FRET is a non‐radiative energy transfer process that occurs between a donor fluorophore in an excited state and an acceptor fluorophore in close spatial proximity (typically 1–10 nm).^[^ ^104^ ^]^ This mechanism relies on dipole‐dipole interactions and is highly dependent on the spectral overlap between the donor's emission and the acceptor's absorption. FRET plays a pivotal role in photo‐energy conversion by enabling highly localized energy transfer with nanometer‐scale precision, making it an ideal tool for applications requiring molecular‐level resolution.^[^ ^70^ ^]^ In biosensing, FRET is extensively applied for the detection of biomolecular interactions, conformational changes, and enzymatic activities by translating these events into quantifiable fluorescent signals.^[^ ^105^ ^]^ Similarly, in bioimaging, FRET serves as a powerful modality for visualizing dynamic processes in live cells and tissues with high sensitivity and specificity.^[^ ^106^ ^]^ In phototheranostics, FRET enhances therapeutic efficacy by facilitating controlled energy transfer to photosensitizers or photothermal agents, thereby improving the efficiency of PDT and PTT. For example, UCNPs have served as NIR‐responsive optical transducers that can penetrate deep tissues through biological windows and activate downstream photosensitizers.^[^ ^107^, ^108^ ^]^ Furthermore, when combined with downconversion and upconversion luminescence processes, FRET‐based nanomaterials provide synergistic opportunities for enhanced imaging contrast, multiplexed detection, and spatiotemporally controlled therapeutic interventions. This integration underscores the versatility of FRET as a mechanism for advancing precision theranostics through photo‐energy transfer.

#### 2.1.4 Phosphorescence in Nanomaterials

Phosphorescence is a photophysical process in which a material emits light over an extended period after being excited. After absorbing energy, an electron transitions from the ground state to the excited singlet state, then undergoes ISC to the triplet state (*S*_*0*_ to *S₁* to *T₁*). The phosphorescence is emitted when the electron transitions from the triplet state back to the ground singlet state (*T₁* to *S₀*). This final transition is spin‐forbidden because the electron's spin orientation changes between the triplet state and the ground state.^[^ ^109^ ^]^ As a result, phosphorescence has a longer lifetime, typically in the millisecond to second (10^−3^–1 s) range, or even longer. Additionally, phosphorescence exhibits a larger Stokes shift compared to fluorescence due to the energy loss during the ISC process and the long‐lived triplet state. Fluorescence and phosphorescence are two competing light emission processes. Phosphorescence is favored in systems with efficient ISC or under conditions like low temperatures or reduced oxygen, which suppress triplet‐state quenching. Otherwise, fluorescence, which involves the faster spin‐allowed transition (*S₁* to *S₀*), is more likely to dominate.

Traditional metal‐based phosphorescent nanomaterials, including iridium (Ir),^[^ ^109^ ^]^ ruthenium (Ru),^[^ ^110^ ^]^ and palladium (Pd)^[^ ^111^ ^]^ based compounds, remain the most extensively studied class. Among these, Ru(II) polypyridine complex TLD‐1433 shows exceptional clinical promise and is currently in phase II trials for bladder cancer treatment.^[^ ^112^ ^]^ Room temperature phosphorescent (RTP) organic materials as an alternative class achieve stable phosphorescence by extending triplet state lifetimes through rigid crystal structures or isomer doping.^[^ ^113^, ^114^ ^]^ For example, Chen et al. demonstrated that commercial carbazole's ultralong phosphorescence at room temperature is induced by trace isomeric impurities, specifically 1H‐benzo[*f*]indole (Bd).^[^ ^114^ ^]^ These isomers act as charge traps, enabling the long‐lived triplet states that are essential for RTP. Metal nanoclusters protected by thiol ligands (e.g., AuNCs (Au_n_(SR)_m_), silver nanoclusters (AgNCs, Ag_n_(SR)_m_) have long‐lived phosphorescence due to ligand‐metal interactions and strong quantum confinement effects at the nanoscale. Mitsui et al. reported that silver‐doped Ag_x_Au_25−x_ nanoclusters enhance phosphorescence by blue‐shifting triplet states, suppressing ISC, and enabling efficient photon upconversion.^[^ ^115^ ^]^ Semiconductor QDs doped with transition metal ions, such as ZnS/CdS QDs doped with Mn^[^ ^116^ ^]^ or Cu,^[^ ^117^ ^]^ combine size‐tunable optical properties with long‐lived emission from phosphorescent dopants, making them useful for detecting specific pathological signals. Finally, hybrid phosphorescent nanomaterials, such as MOFs, can be tuned for RTP by modifying organic ligands, engineering structural rigidity, and incorporating guest molecules to enhance spin‐orbit coupling while restricting non‐radiative transitions.^[^ ^118^ ^]^ Xue et al. introduced fluorine atoms into calcium‐based MOFs (5FCa‐MOF) to create intermolecular aggregate interlocking that significantly enhances room‐temperature phosphorescence, achieving a remarkable lifetime of 264 ms and visible afterglow lasting about 2 s.^[^ ^119^ ^]^

### 2.2 Photon‐to‐Heat Conversion in Nanomaterials

The photothermal effect represents a distinct energy conversion process in which absorbed light energy is transformed into thermal energy through three physical mechanisms: plasmonic localized heating, nonradiative relaxation‐based photothermal conversion in semiconductors, and thermal vibration of molecules.^[^ ^120^ ^]^ Plasmonic localized heating occurs primarily in metallic nanostructures (such as Au, Ag, Al, Cu, Pd, and Pt nanoparticles) through localized surface plasmon resonance (LSPR).^[^ ^121^ ^]^ When the photon energy matches the LSPR band, free conduction electrons in metals collectively oscillate with the incident light's electric field, creating strong local field enhancement. The absorbed energy is then dissipated through a cascade process. Briefly, landau damping (10^−15^–10^−13^ s) transfers plasmon energy to electron‐hole pairs, followed by electron‐electron collisions (10^−13^–10^−12^ s) converting energy to heat. Then, the electron‐phonon scattering (few to hundreds of picoseconds) transfers energy to the metallic lattice, and finally phonon‐phonon collisions (10^−10^–10^−8^ s) dissipate thermal energy to the surroundings.^[^ ^122^, ^123^ ^]^ In semiconductor materials (such as metal oxides (TiO_2_, Ti_2_O_3_, MoO_3_) and chalcogenides (Cu_2x_S, Cu_2x_Se)), non‐radiative photothermal conversion occurs when absorbed photons with energy equal to or greater than their bandgap generate electron‐hole pairs. These electron‐hole pairs then relax by transferring energy to the crystal lattice as heat rather than emitting light.^[^ ^124^ ^]^ In carbon‐based nanomaterials (e.g., graphene, graphene oxide (GO), reduced graphene oxide (rGO), carbon nanotubes (CNT), CDs etc.), and organic polymers (e.g., polythiophene (PT), polypyrrole (PPy), polydopamine (PDA)), the photo‐thermal conversion process involves the excitation of loosely held π electrons from the ground state (HOMO) to a higher energy state (LUMO).^[^ ^125^ ^]^ The excited electrons then relax to the ground state through vibration‐electron coupling, releasing the excess energy as heat. Quantitatively, the photothermal conversion efficiency (PTCE, η) can be determined by the ratio of heat generation and laser power input, which can be written as:

(2) $$\eta =\frac{hs\left(\Delta T\right)-Q_{dis}}{I\left(1-10^{-A_{\lambda }}\right)}$$

where h is the heat transfer coefficient, s is the surface area of the absorbing material, ΔT is the temperature increase of the system after laser irradiation, Q_dis_ stands for the heat dissipated by the surrounding environment, I is the power density of the incident laser (W cm^−^^2^), and A is the absorbance of the nanomaterial at the laser wavelength (λ). The generated heat transfers to the surroundings primarily through conduction and convection (with typically negligible radiation), driven by thermal gradients between the heated material and its environment.

Recent studies have highlighted the great potential of 2D nanomaterials, such as MXenes, TMDs, and black phosphorus (BP) nanosheets, as powerful photothermal agents, especially for applications in the NIR‐II region.^[^ ^126^ ^]^ MXenes (e.g., Ti₃C₂, Nb₂C, Mo₂C) stand out due to their superior photothermal efficiency derived from plasmonic‐driven mechanisms.^[^ ^127^, ^128^ ^]^ For example, Nb₂C nanosheets exhibited excellent thermal responsiveness, achieving temperatures up to 50 °C within 5 min at a low concentration (40 µg mL^−1^) under irradiation with a 1064 nm laser.^[^ ^129^ ^]^ TMDs predominantly rely on exciton generation followed by non‐radiative recombination pathways. Huang et al. reported Cu‐MoS₂ nanocomposites with a photothermal conversion efficiency of 45.44% in the NIR‐II region for effective killing of tumors.^[^ ^130^ ^]^ BP is known for its excellent biosafety and biodegradability, but its performance in the NIR‐II region remained limited. Wu et al. reconstructed the BP lattice into nickel phosphide QDs (Ni₂P QDs) through topochemical transformation, significantly improving the light absorption at 1064 nm and the photothermal conversion efficiency to 43.5%.^[^ ^131^ ^]^ Additionally, organic photothermal materials have made significant advancements through innovative molecular design strategies, particularly in addressing the critical challenges of NIR‐II absorption and photothermal conversion efficiency. Kong and colleagues introduced the “electron–donor iteration” strategy through CR‐(DPA)3‐T dendrimer design, achieving 60.4% NIR‐II conversion efficiency by optimizing D‐A effects and intramolecular motions.^[^ ^132^ ^]^ Yang et al. advanced the field by introducing the “motion and stillness” concept in NIR‐II AIEgens (Y5‐2BO‐2BTF), which achieved an ε of 1.06 × 10^5^ M^−1^ cm^−1^ and 77.8% conversion efficiency through the synergy of a highly planar A–D–A configuration and placement of ─CF₃ groups.^[^ ^133^ ^]^ Parallel efforts in hybrid materials have integrated multiple functional components to achieve enhanced stability and functionality. Liao et al. developed a perylene‐based MOF with unique spatial arrangements that enable panchromatic absorption and efficient NIR‐I/II photothermal conversion through inherent charge transfer interactions while maintaining exceptional stability.^[^ ^134^ ^]^

### 2.3 Photon‐to‐Electron Conversion in Nanomaterials

Photoelectric conversion represents a fundamental process where incident photons are transformed into electrical signals through light‐matter interactions in various materials. This phenomenon underlies various optoelectronic devices, such as photovoltaic cells, photodetectors, PEC systems, and photocatalytic materials. The conversion mechanism comprises several sequential steps. Initially, photon absorption occurs on femtosecond timescales (10^−15^ s), exciting electrons to higher energy states. This is followed by hot carrier relaxation and recombination, which follow on picosecond timescales (10^−12^ s) due to the electron‐photon coupling. Carrier generation and separation typically occur on picosecond to nanosecond timescales (10^−12^–10^−9^ s), with the specific mechanisms varying by material class.^[^ ^135^ ^]^ In semiconductors, when photon energy exceeds the bandgap (E_g_), electrons are excited from the valence to the conduction band, creating electron–hole pairs (e^−^/h^+^). These charge carriers can be separated by built‐in electric fields (e.g., in p‐n heterojunctions) or interfaces of heterojunctions, generating a photocurrent.^[^ ^136^ ^]^ The charge separation efficiency (η_CS_) can be expressed as:

(3) $$\eta_{\mathrm{CS}}=\frac{K_{\mathrm{CS}}}{K_{\mathrm{rec}}+K_{\mathrm{CS}}}$$

where *k*_CS_ is the rate constant for charge separation, *k*_rec_ is the rate constant for electron‐hole recombination. High‐performance devices have *k*_CS_≫*k*_rec_, ensuring efficient exciton dissociation and minimal recombination. After separation, charge carriers must migrate to electrodes to be collected as current. Charge transport efficiency (η_CT_) can be described by:

(4) $$\eta_{\mathrm{CT}}=\frac{\tau_{\mathrm{rec}}}{\tau_{\mathrm{rec}}+\tau_{\mathrm{trans}}}$$

where τ_rec_ is the carrier recombination lifetime, and τ_trans_ is the transport time to reach electrodes. Efficient charge collection requires long recombination lifetimes and short transport times. The incident photon‐to‐current efficiency (IPCE) is expressed as:

(5) $$\mathrm{IPCE}=\eta_{\mathrm{abs}}\;\times \eta_{\mathrm{cs}}\times \;\eta_{\mathrm{cT}}=1240\times \frac{J_{ph}}{\lambda \times p_{light}}$$

where η_abs_ is the light‐harvesting efficiency (governed by the Beer–Lambert law), J_ph_ is the photocurrent density measured under illumination, P_light_ is the incident light power density, λ is the wavelength of the incident light, and 1240 is a constant derived from the relationship between the energy of the photon and their wavelength. In metals, the photoelectric effect occurs when the energy of incident photons exceeds the material's work function, leading to the direct ejection of electrons from the surface into the vacuum. In hybrid systems, such as perovskites and organic–inorganic heterojunctions, they take advantage of interfacial charge transfer and energy level alignment to improve carrier separation and increase their lifetime.

Ideal photoactive materials should exhibit high absorption coefficients to maximize light harvesting and efficient carrier transport to minimize recombination losses, ensuring effective charge transfer. Traditional inorganic semiconductors such as Si, GaAs, CdS, Bi₂S₃, and CdTe have dominated applications like photovoltaics and photoelectrochemistry due to their excellent stability and high conversion efficiencies.^[^ ^137^ ^]^ However, their rigid crystal structures, toxicity (e.g., Cd‐based compounds), and limited bandgap tunability have driven the exploration of alternative materials with enhanced flexibility and optimized interfacial charge dynamics. Metal oxides, like TiO₂, ZnO, WO_3_, and CuO have emerged as important wide‐bandgap semiconductors often employed as electron‐transport layers due to favorable band alignment and robustness.^[^ ^30^, ^138^ ^]^ Zhang et al. demonstrated a type‐II heterojunction composed of TiO₂–CeO₂ nanotubes decorated on carbon fiber paper, significantly enhancing photoreactive surface areas and promoting rapid electron transport.^[^ ^139^ ^]^ Emerging 2D materials, particularly metal chalcogenides like MoS₂ and WS₂, offer tunable bandgaps and high surface‐to‐volume ratios, enabling efficient light‐matter interaction and charge separation.^[^ ^140^ ^]^ Seo et al. achieved controllable growth of MoS₂ nanosheets on WO₃ nanorods, forming a staggered heterojunction with improved charge separation and PEC efficiency.^[^ ^141^ ^]^ MXenes have gained prominence for their intrinsic conductivity and interfacial compatibility. Ye et al. reported the in situ growth of flower‐like BiOI structures on negatively charged Ti₃C₂ MXene nanosheets to form a robust Schottky junction, which significantly enhanced the charge separation through the built‐in electric field and reduced the interfacial contact resistance.^[^ ^142^ ^]^ Carbon‐based materials, such as GO, rGO, and g‐C₃N₄, combine high carrier mobility with tunable electronic properties and ease of functionalization.^[^ ^31^, ^143^ ^]^ Chen et al. highlighted the promising potential of g‐C₃N₄ as a metal‐free semiconductor in developing a sensitive PEC aptasensor for detecting Staphylococcus aureus.^[^ ^144^ ^]^ Additionally, integrating plasmonic metal nanoparticles (Au, Ag, Pd, Pt) can markedly enhance optical absorption via surface plasmon resonance (SPR).^[^ ^145^ ^]^ Zhang et al. demonstrated this by developing a CuO/Au nanoparticle Schottky junction, which creates an irreversible electron‐transfer pathway and significantly enhances photoelectric conversion efficiency through the plasmon‐induced enhancement of visible light absorption.^[^ ^146^ ^]^ Organic semiconductors like polyaniline (PAni) and polyindole (PInd) offer advantages of solution processability, cost‐effectiveness, and structural tunability, though they generally show lower carrier mobility compared to inorganic counterparts.^[^ ^147^ ^]^ Now, hybrid materials have bridged these gaps. Liu et al. reported a CdS/Ni‐CAT‐1 MOF Z‐scheme heterojunction as a photoelectrode for PEC sensor construction, successfully combining the photocatalytic activity of CdS with the superior conductivity and structural benefits of the Ni‐CAT‐1 MOF, resulting in efficient charge transfer and significantly reduced recombination of photogenerated carriers.^[^ ^148^ ^]^ Recently, lead‐halide perovskites (e.g., CH₃NH₃PbI₃) have revolutionized photovoltaics with their high absorption coefficients, long carrier diffusion lengths, and remarkably high carrier mobilities. Ren et al. embedded CsPbX₃ PeQDs into mesoporous MOF‐5 to obtain significantly enhanced thermal‐/photo‐/long‐term stability and high PL performance with quantum yields reaching up to 52–56%.^[^ ^149^ ^]^

### 2.4 Photon‐to‐Acoustic Conversion in Nanomaterials

The mechanism of PA signal generation involves three key processes: photoabsorption, photothermal conversion, and thermoacoustic conversion. Similar to photo‐to‐thermal conversion processes, it begins with photon absorption and electronic excitation from the ground state (*S₀*) to excited states (*S₁, S₂)* and follows non‐radiative decay channels (such as internal conversion and vibrational relaxation), converting optical energy into thermal energy at molecular scales. This localized heating induces rapid thermoelastic expansion of the surrounding medium, generating pressure waves that propagate as acoustic signals. These ultrasonic waves then propagate through the medium and are acquired to generate PA images.^[^ ^150^ ^]^ The generated photoacoustic signal amplitude (P_0_) can be determined by:

(6) $$P_{0}=\Gamma \mathrm{aF}$$

where Γ is Grüneisen parameter, a is the optical absorption coefficient, and F is the optical fluence. The Grüneisen parameter is a dimensionless coefficient characterizing the efficiency of thermal‐to‐mechanical energy conversion, and it depends on temperature and medium properties:

(7) $$\Gamma =\frac{\beta v^{2}}{C_{p}}$$

where β is the thermal expansion coefficient, *v* is the speed of sound, and *C*_p_ is the specific heat capacity at constant pressure. Temporally, the process spans from picosecond (10^−12^ s) photon absorption to nanosecond (10^−9^ s) thermal expansion and microsecond (10^−6^ s) acoustic propagation, while spatially expanding from nanometer (nm) scale molecular absorption to millimeter–centimeter (mm‐cm) acoustic propagation.^[^ ^151^ ^]^

PA contrast agents can be broadly categorized into endogenous and exogenous types. Endogenous contrast agents include intrinsic components like hemoglobin, myoglobin, melanin, and lipids, which are inherently safe but suffer from limited sensitivity, specificity, and restricted application scenarios due to their fixed optical properties.^[^ ^152^ ^]^ In contrast, exogenous agents are engineered to enhance signal‐to‐noise ratios, allow tunable absorption in the near‐infrared (NIR‐I/II) range, and facilitate molecular targeting. Key exogenous agents include metallic nanomaterials,^[^ ^153^ ^]^ 2D nanomaterials (e.g., transition metal sulfides (CuS, Ag_2_S, Bi_2_S_3_, MoS_2_), MXenes, and BP),^[^ ^154^ ^]^ carbon‐based nanoparticles (e.g., CNT, graphene and its derivatives (GO, rGO), and CDs),^[^ ^155^ ^]^ polymer‐based nanomaterials (e.g., porphyrin‐lipid nanoparticles and semiconducting polymer nanoparticles),^[^ ^156^ ^]^ and organic molecules (e.g., indocyanine green (ICG) and AIEgens).^[^ ^157^, ^158^ ^]^ Emerging trends in PAI include biomimetic agents, such as cell membrane‐coated nanoparticles for improved tumor targeting and immune evasion,^[^ ^159^ ^]^ and stimuli‐responsive agents like pH‐ or enzyme‐activated probes for microenvironment‐specific signal amplification.^[^ ^160^, ^161^, ^162^ ^]^ PAI is applied in molecular imaging, enabling targeted detection of biomarkers for early disease diagnosis, and in image‐guided therapy for real‐time monitoring of photothermal or photodynamic treatment efficacy. However, challenges remain, including biosafety concerns such as long‐term toxicity in PA agents, and the complexity of standardizing PA signal quantification due to nonlinear intensity‐concentration relationships.

### 2.5 Photo‐Triggered SERS in Nanomaterials

Photo‐triggered SERS represents a plasmonic‐enhanced light‐molecule interaction process where incident photons undergo inelastic scattering through interactions with molecular vibrations. The mechanism begins with the excitation of localized surface plasmons on metallic surfaces within femtoseconds (10⁻^15^ s), generating intense electromagnetic fields in hot spots (e.g., nanogaps between metallic surfaces) or at sharp tips and edges of nanostructures, thus amplifying the interaction between the light and molecular vibrations.^[^ ^163^ ^]^ As a result, Raman scattering occurs on a picosecond timescale (10⁻^12^ s),  driven by ultrafast plasmon relaxation dynamics. The scattered photons are further amplified by the plasmonic structures. This entire process occurs within highly confined spatial dimensions of 1–10 nm at the metal‐molecule interface. The enhancement in SERS is primarily attributed to electromagnetic and chemical enhancement. The enhancement factor electromagnetic enhancement ($EF_{SERS}^{EM}$) can be expressed as:

(8) $$EF_{\mathrm{SERS}}^{\mathrm{EM}}={\left[\frac{E\left(\omega_{L}\right)}{E_{0}\left(\omega_{L}\right)}\right]}^{2}\times {\left[\frac{E\left(\omega_{R}\right)}{E_{0}\left(\omega_{R}\right)}\right]}^{2}$$

where E and E₀ are the enhanced and incident electric fields at excitation laser (ω_L_) and scattered Raman (ω_R_) frequencies, respectively. This equation can be further simplified for small Raman shifts (ω_L_ ≅ ω_R_). In this case:

(9) $$EF_{\mathrm{SERS}}^{\mathrm{EM}}={\left[\frac{E\left(\omega_{L}\right)}{E_{0}\left(\omega_{L}\right)}\right]}^{4}$$

referred to the zero Stokes shift limit of the |E|^4^ approximation. The electromagnetic enhancement can amplify Raman signals by up to 10^10^. Chemical enhancement, while contributing a smaller but significant portion of the overall enhancement, involves charge transfer between the adsorbed molecule and the metal surface. The enhancement factor (EF^Chem^) can be expressed as:

(10) $$EF_{SERS}^{Chem}=\frac{\sigma_{k}^{ads}}{\sigma_{k}^{free}}$$

where $\sigma_{k}^{ads}$ and $\sigma_{k}^{free}$ are the Raman cross‐sections of the k‐th vibrational mode of the adsorbed and the free molecule, respectively. The chemical enhancement can amplify Raman signals by up to 10^4^. Therefore, the (total) SERS enhancement factor G_SERS_ combines both mechanisms as:

(11) $$G_{SERS}=EF_{SERS}^{EM}\;EF_{SERS}^{Chem}$$

which can amplify Raman signals by up to 10^14^, thus enabling detection at trace levels.^[^ ^163^ ^]^

SERS substrates are typically fabricated using materials that support plasmonic resonance. Traditional SERS substrates primarily utilize noble metals, with Au and Ag being the most common.^[^ ^164^, ^165^ ^]^ Au offers superior biocompatibility and chemical stability, making it particularly suitable for biomedical applications, while Ag typically provides stronger enhancement but is more prone to oxidation. Recent developments have expanded the material options to include metal alloys such as Au‐Pt and Au‐Ag, which offer the ability to engineer band structures, shift plasma frequencies, and modify interband transition thresholds.^[^ ^166^, ^167^ ^]^ Beyond noble metals, the development has also expanded to include TMDs,^[^ ^168^ ^]^ graphene analogs,^[^ ^169^ ^]^ MXenes,^[^ ^170^ ^]^ BP,^[^ ^171^ ^]^ and MOFs.^[^ ^172^ ^]^ These alternative materials offer advantages such as reduced heat generation and a wider variety of functional groups for surface modification.

### 2.6 Photon‐to‐ROS Conversion in Nanomaterials

The photodynamic effect represents a photochemical process where photosensitizers convert light energy into ROS through interaction with molecular oxygen. The mechanism is initiated when photosensitizer molecules absorb light of a specific wavelength and are excited from the ground state (*S₀*) to a short‐lived excited singlet state (*S₁*) within nanoseconds (10^−9^ s). These excited molecules then undergo ISC (10^−6^ s) to the longer‐lived triplet state (*T₁*), ultimately transferring energy to molecular oxygen to generate ROS (10^−3^ s).^[^ ^173^ ^]^ The efficiency of this ISC process is characterized by the triplet quantum yield (Φ_t_), which can be determined experimentally by measuring the triplet molar absorption coefficient (ɛ_t_) through transient absorption spectroscopy:

(12) $$\varepsilon_{T}=\left(\frac{\Delta OD_{t}}{\Delta OD_{s}}\right)$$

where ɛ is the ground‐state molar absorption coefficient, and ΔOD_t_ and ΔOD_s_ represent amplitude parameters obtained from exponential fittings of transient absorption and singlet depletion decays, respectively. Once in the triplet state, ROS are generated via two principal pathways. The Type I mechanism involves electron transfer between excited photosensitizers and biological substrates, resulting in the formation of free radicals such as hydroxyl radicals (•OH), hydrogen peroxide (H₂O₂), and superoxide anions (O_2_^•−^).^[^ ^174^ ^]^ Conversely, the Type II mechanism involves energy transfer from the photosensitizer triplet state to ground state oxygen (^3^O₂), generating highly cytotoxic singlet oxygen (¹O₂):

(13)

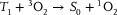




¹O₂ has a short lifetime and limited diffusion distance (≈10–20 nm), with the ensuing oxidative reactions affecting surrounding biomolecules.^[^ ^175^ ^]^ Typically, the effectiveness of a photosensitizer for PDT is defined by long triplet state lifetime (τ_t_), high triplet quantum yields (Φt), and efficient singlet oxygen generation. The τ_t_ refers to how long the photosensitizer remains in its excited triplet state before returning to the ground state. A longer lifetime allows the excited molecule more time to interact with molecular oxygen, enhancing the production of ROS. Although Type II PDT is often preferred due to its therapeutic efficiency and lower energy requirements, its dependency on oxygen limits its use in hypoxic environments. Type I PDT offers a viable alternative in such conditions and hybrid strategies that combine both mechanisms can optimize therapeutic outcomes.

The evolution of PDT has been marked by significant advances in photosensitizer development. First‐generation photosensitizers (like Photofrin) faced limitations of poor water solubility, prolonged photosensitivity, and suboptimal absorption profiles.^[^ ^176^ ^]^ Although second‐generation photosensitizers (such as chlorine and phthalocyanines) have improved NIR absorption and singlet oxygen yields, challenges with aggregation and non‐specific biodistribution persist, driving the development of nanomaterial‐based systems.^[^ ^177^ ^]^ Nanomaterial‐based photosensitizers can be categorized into three functional groups based on their roles in PDT. The first group consists of nanocarriers for photosensitizer delivery, designed to enhance solubility, improve tumor targeting, and prevent premature release of the photosensitizer. Biodegradable carriers, such as polymeric nanoparticles,^[^ ^178^ ^]^ liposomes,^[^ ^179^ ^]^ and dendrimers,^[^ ^180^ ^]^ offer improved pharmacokinetic properties and controlled drug release, while non‐biodegradable carriers, including silica nanoparticles,^[^ ^181^, ^182^ ^]^ gold nanoparticles (AuNPs),^[^ ^183^ ^]^ provide chemical stability, modularity, and additional functionalities like imaging and PTT compatibility. Recently, DNA nanostructures, which are precisely engineered nanoscale architectures formed through the self‐assembly of DNA strands, have been used as programmable and biocompatible carriers for PDT.^[^ ^184^ ^]^ Wang et al. developed a light‐activated multifunctional DNA nanodrug (MCD@TMPyP4@DOX) for the targeted delivery of multiple therapeutic agents, including DNAzyme, the photosensitizer TMPyP4, and the chemotherapeutic drug doxorubicin (DOX), to effectively reverse multidrug resistance in cancer.^[^ ^185^ ^]^ The second group includes direct photosensitizing nanoparticles, such as carbon‐based materials (CNT, GQD, C60, C70),^[^ ^186^ ^]^ TiO_2_,^[^ ^187^ ^]^ and ZnO,^[^ ^188^ ^]^ which generate ROS upon light activation without requiring additional photosensitizer loading. Recently, novel nanomaterials have emerged as highly efficient photosensitizers with excellent ROS generation capabilities. For example, MOFs can either act as intrinsic photosensitizers themselves or enable the efficient loading of photosensitizers. Notable advancements include self‐oxygen‐generating MOFs, which incorporate catalase or oxygen‐containing ligands to alleviate tumor hypoxia and enhance the efficacy of PDT.^[^ ^189^ ^]^ BP nanosheets with broad‐spectrum light absorption ranging from UV to NIR can autonomously generate ROS. Lan et al. used BP as both PTT/PDT agents to induce hyperthermia, mitochondrial damage, and immunogenic cell death (ICD) for cancer therapy.^[^ ^190^ ^]^ Meanwhile, AIEgens have been developed as versatile “photosensitizer‐theranostic” platforms. Gao et al. achieved NIR‐II AIEgens that simultaneously emitted long wavelengths and efficient singlet oxygen by optimizing the acceptor–donor–acceptor–donor–acceptor (A‐D‐A‐D‐A) structure.^[^ ^191^ ^]^ AuNCs with diameters less than 2 nm represent a class of atomically precise photosensitive materials. Liu et al. utilized ICG‐sensitized ultra‐smallAuNCs with atomic‐precision size effects to enhance antibacterial PDT, enabling near‐infrared light absorption, improved ROS production, and effective biofilm penetration, thereby disrupting quorum sensing, virulence, and bacterial biofilms.^[^ ^192^ ^]^ The third group comprises energy‐transducing nanoparticles, including QDs^[^ ^107^ ^]^ and UCNPs,^[^ ^108^ ^]^ which convert one form of energy to another to activate the attached photosensitizer indirectly. The progression from molecular to nanomaterial‐based photosensitizers has enhanced the therapeutic potential of PDT.

**Figure 3:**
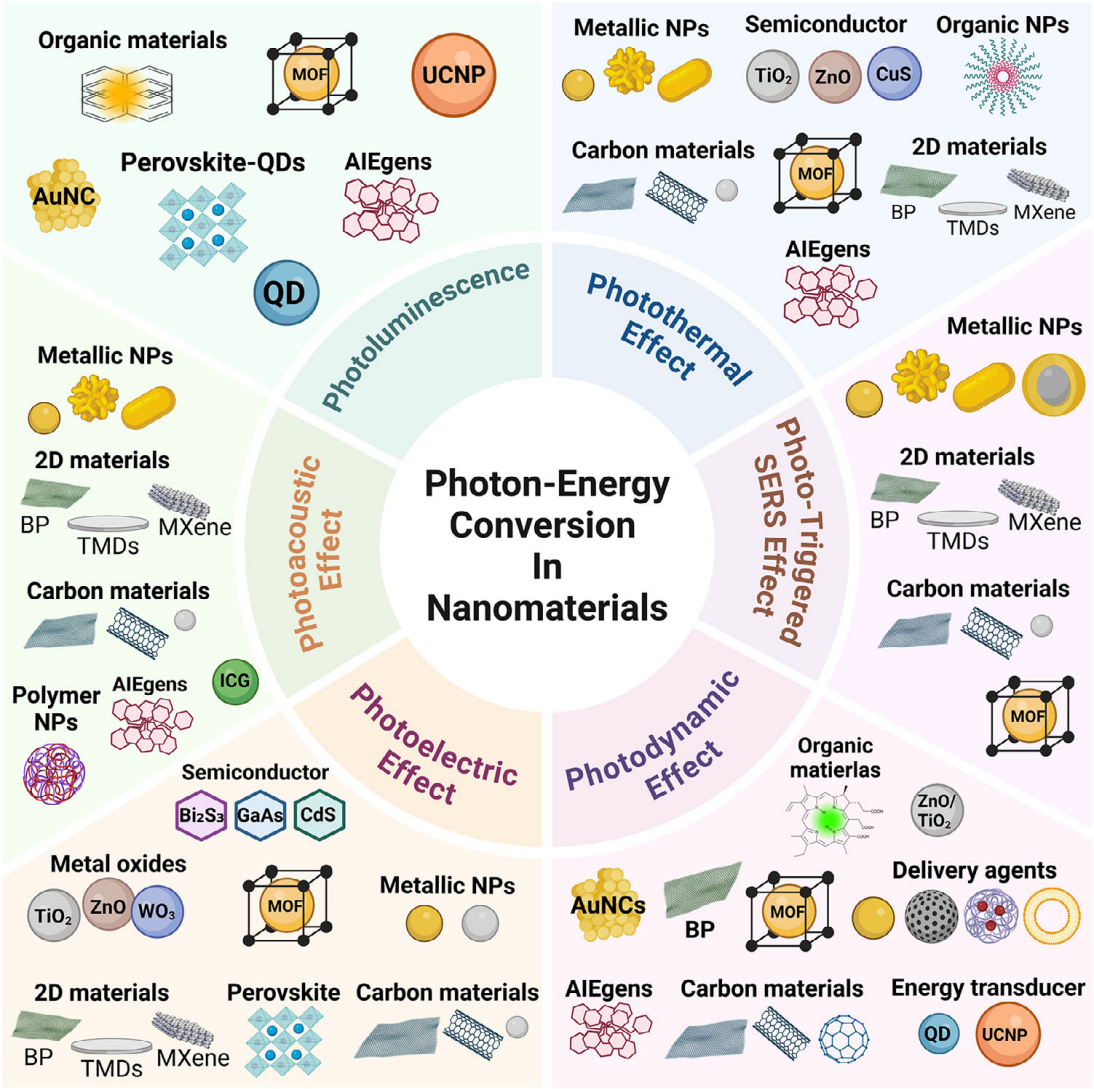
Photo‐energy conversion in nanomaterials. Created with BioRender.com.

The different categories of nanomaterials related to photo‐energy conversion are listed in **Figure** **3** and **Table** **2**.

**Table 2:** Photo‐energy conversion in nanomaterials.

Photo‐energy conversion effects	Material class	Typical examples	Size range	Absorbance range	Key properties in photo‐energy conversion effects
Photoluminescence	QDs	ZnS/CdS,^[^ ^57^, ^116^, ^117^ ^]^ GQDs,^[^ ^58^, ^59^, ^60^, ^61^, ^62^, ^63^, ^64^, ^65^, ^66^ ^]^ CDs,^[^ ^67^ ^]^ WS₂ QDs,^[^ ^72^ ^]^ MoS₂ QDs,^[^ ^73^ ^]^ MXene‐QDs,^[^ ^74^ ^]^ BP QDs,^[^ ^72^ ^]^ g‐C₃N₄‐QDs,^[^ ^71^ ^]^ Perovskite QDs^[^ ^77^, ^78^ ^]^	1–10 nm	UV–vis–NIR‐II	Size‐tunable excitation/emission, high quantum yield
	MOFs	Rh6G@UIO‐66‐NH_2_,^[^ ^82^ ^]^ UiO‐66(OH)_2_@Y‐TCPP^[^ ^83^ ^]^ AuNCs/GQDs@ZIF‐8,^[^ ^84^ ^]^ 5FCa‐MOF^[^ ^119^ ^]^	50–200 nm	UV–vis–NIR‐II	High porosity, guest‐dependent luminescence, large surface area
	AIEgens	MeTTh,^[^ ^89^ ^]^ TPE‐AMC/TPE‐TCF,^[^ ^90^ ^]^ TPET,^[^ ^91^ ^]^	20–200 nm	UV–vis–NIR‐II	Aggregation‐induced emission, tunable luminescence, low self‐quenching
	UCNP	lanthanide‐doped NPs, NaYF₄:Yb,Er/Tm^[^ ^97^ ^]^	10–100 nm	NIR‐I/II	NIR‐to‐vis upconversion, deep tissue penetration, minimal autofluorescence
	Metal nanoclusters	AuNCs, Au_n_(SR)_m_^[^ ^115^ ^]^	<2 nm	UV–vis–NIR‐II	Ultra‐small size, size‐dependent emission, large surface‐to‐volume ratio
Photothermal effect	Metallic NPs	Au, Ag, Cu, Al, Pd, Pt nanoparticles/nanorods/nanostars^[^ ^121^ ^]^	10–200 nm	Vis‐NIR	LSPR‐driven heating, tunable plasmon resonance, high conversion efficiency
	Semiconductors	TiO_2_, Ti_2_O_3_, MoO_3_, Cu_2x_S, Cu_2x_Se)^[^ ^124^ ^]^	20–200 nm	UV–vis–NIR	Bandgap absorption, high thermal stability, broad‐spectrum light harvesting
	Carbon materials	Graphene, GO, rGO, CNTs, CDs^[^ ^125^ ^]^	5–200 nm	UV–vis–NIR‐II	High thermal conductivity, broadband absorption, stability
	2D materials	MXenes (Ti₃C₂,^[^ ^128^ ^]^ Nb₂C,^[^ ^129^ ^]^), TMDs (MoS₂),^[^ ^130^ ^]^ BP^[^ ^131^ ^]^	<10 nm thickness	UV–vis–NIR‐II	Layered structure, strong NIR absorption, rapid heat dissipation, high surface area
	Organic materials	Dendrimers^[^ ^132^ ^]^	20–200 nm	Vis‐NIR	Tailored molecular absorption, biocompatibility
	AIEgens	Y5‐2BO‐2BTF^[^ ^133^ ^]^	20–200 nm	Vis‐NIR‐II	Enhanced photothermal effect upon aggregation, good photostability, molecular structure‐dependent properties
	MOFs	Perylene‐based MOFs^[^ ^134^ ^]^	50–200 nm	Vis‐NIR‐II	Excellent thermal stability, ligand‐mediated light absorption
Photoelectric effect	Semiconductors	Si, GaAs, CdS, Bi₂S₃, CdTe^[^ ^137^ ^]^	Variable thin film or nanoparticles (<500 nm)	Vis‐NIR	Direct bandgap, high charge mobility, efficient electron‐hole separation
	Metal oxides	TiO₂,^[^ ^30^, ^139^ ^]^ ZnO,^[^ ^138^ ^]^ WO_3_^[^ ^141^ ^]^	10–200 nm	UV‐Vis	Wide bandgap, photocatalytic activity, stability under irradiation
	2D materials	MoS₂,^[^ ^140^, ^141^ ^]^ MXenes (Ti₃C₂)^[^ ^142^ ^]^	<10 nm (thickness)	Vis‐NIR	Tunable bandgap, high carrier mobility, large surface area
	Carbon materials	GO, rGO, g‐C₃N₄,^[^ ^143^ ^]^ GQD,^[^ ^31^ ^]^ CDs	5–200 nm	UV–vis–NIR	High conductivity, broadband absorption, interfacial charge transfer
	Metallic NPs	CuO/Au^[^ ^146^ ^]^	nm–µm	UV‐Vis	Plasmon‐enhanced light trapping, hot‐electron injection
	MOFs	Ni‐CAT‐1^[^ ^148^ ^]^	50–500 nm	UV‐Vis	Enhanced charge separation, improved photocurrent response, stability
	Perovskites	CsPbX₃ PeQDs^[^ ^149^ ^]^	1–20 nm (QDs)	UV‐Vis	High absorption coefficient, long carrier lifetimes, tunable bandgap, poor environmental stability
Photoacoustic effect	Metallic NPs	Au, Ag nanorods, nanocages^[^ ^153^ ^]^	10–100 nm	NIR‐I/II	Plasmonic absorption, strong thermal expansion, high signal‐to‐noise ratio
	2D materials	MXenes, BP, TMDs^[^ ^154^ ^]^	<10 nm (thickness)	NIR‐I/II	Broadband NIR absorption, high thermal conductivity
	Carbon materials	CNT, graphene, GO, rGO^[^ ^155^ ^]^	20–200 nm	UV–vis–NIR	Broadband absorption, photostability, biocompatibility
	Polymer NPs	PPy, PDA, semiconducting polymer NPs^[^ ^156^ ^]^	50–500 nm	NIR	High photothermal conversion, design flexibility, biodegradability
Photo‐triggered SERS effect	Metallic NPs	Au, Ag, Au@Ag core‐shell^[^ ^164^, ^165^ ^]^	10–100 nm	Vis‐NIR	Plasmonic hotspots, electromagnetic enhancement, single‐molecule sensitivity
	2D materials	MoS₂, WS₂, Graphene,^[^ ^159^ ^]^ MXenes,^[^ ^170^ ^]^ BP^[^ ^171^ ^]^	5–200 nm	UV–vis–NIR	Charge transfer enhancement, atomically smooth surfaces, chemical stability
Photodynamic effect	Organic photosensitizers	Porphyrins,^[^ ^176^ ^]^ chlorins (Ce6), BODIPY, Zn‐phthalocyanines, AIEgens^[^ ^191^ ^]^	<50 nm	Vis‐NIR	High ROS quantum yield, targeted excitation, tunable singlet‐oxygen generation
	Inorganic photosensitizers	Carbon‐based materials (CNT, GQD, C60, C70),^[^ ^186^ ^]^ TiO_2_,^[^ ^187^ ^]^ ZnO,^[^ ^188^ ^]^ BP^[^ ^190^ ^]^ AuNCs^[^ ^192^ ^]^	2–100 nm	UV–vis–NIR	Bandgap‐driven ROS, broad‐spectrum absorption, photostability
	MOFs	CuTz‐1‐O_2_@F127^[^ ^189^ ^]^	50–200 nm	UV–vis–NIR	Self‐oxygenation, hypoxia alleviation, enhanced PDT efficacy
	Delivery agents	Liposomes,^[^ ^179^ ^]^ dendrimers,^[^ ^180^ ^]^ polymeric NPs,^[^ ^178^ ^]^ silica nanoparticles,^[^ ^181^, ^182^ ^]^ AuNPs,^[^ ^183^ ^]^ DNA nanostructures^[^ ^185^ ^]^	20–500 nm	/	Targeted delivery, improved solubility, controlled release
	Energy transducing systems	QDs^[^ ^107^ ^]^ and UCNPs^[^ ^108^ ^]^	20–100 nm	NIR	NIR‐to‐vis conversion, deep‐tissue activation, multiplexed therapy

## 3 Applications of Photo‐Energy Conversion in Precision Theranostics

Photo‐responsive nanomaterials can respond to specific wavelengths in the electromagnetic spectrum (from UV to NIR) and convert photon energy into diverse outputs (e.g., light, heat, ROS, electrical signals, etc.) for diagnostic and therapeutic applications. In this section, we discuss the applications of photo‐triggered nanoplatforms in biosensing, bioimaging, and theranostics, with a focus on cancer, neurodegenerative diseases, retinal degeneration, and osteoarthritis.

### 3.1 Cancer

Despite significant advances in medical science, cancer remains a global health challenge. Traditional diagnostic methods, such as tissue biopsy and standard imaging techniques, often suffer from invasiveness, low sensitivity, delayed detection, and inability to provide comprehensive molecular profiling.^[^ ^46^ ^]^ Similarly, conventional therapeutic strategies, including surgery, chemotherapy, and radiotherapy, are frequently accompanied by severe side effects, drug resistance, and incomplete tumor elimination.^[^ ^45^ ^]^ The heterogeneous nature of cancer, coupled with its complex microenvironment and adaptive mechanisms, often leads to treatment failure and cancer recurrence.^[^ ^193^, ^194^ ^]^ These challenges have accelerated the development of innovative diagnostic and therapeutic strategies, leading to the emergence of novel biosensing platforms, molecular imaging probes, and light‐responsive theranostic agents that promise more precise cancer management.

#### 3.1.1 Biosensing

Photo‐energy conversion‐based nanomaterials are used for cancer diagnosis through three main detection mechanisms: PL‐based biosensing, SERS‐based biosensing, and PEC biosensing. These platforms enable rapid and sensitive detection of cancer‐specific biomarkers, including circulating tumor DNA (ctDNA), microRNA (miRNA), cancer exosomes, and circulating tumor cells (CTCs).^[^ ^46^ ^]^

##### FRET‐Based Biosensing

**Figure 4:**
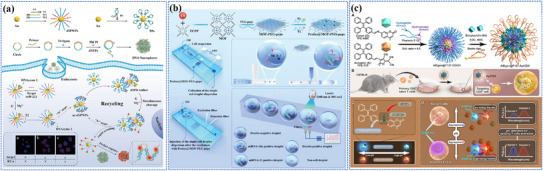
Photo‐triggered FRET‐based biosensors for cancer diagnosis. a) Schematic illustration of the preparation and rapid walking principle of the dSPD walker (Composed of ac‐dSPWPs and TPs) for rapid and ultrasensitive detection and high‐contrast intracellular imaging of miR‐221 in living cells. Reproduced with permission.^[^ ^200^ ^]^ Copyright 2024, American Chemical Society. b) The scheme for the fabrication of MOF‐PEG‐peps nanocomposite‐based nanosensor for simultaneous detection of miR‐21 and miR‐10a. Reproduced with permission.^[^ ^204^ ^]^ Copyright 2022, Wiley‐VCH. c) Schematic illustration of the CD^8+^ T cell‐selective nanoprobe (AIEgens@F127‐AptCD8) for dynamic sensing of intracellular pH to fluorescently distinguish activated CD^8+^ T cells from naiv̈e CD^8+^ T cells. Reproduced with permission.^[^ ^90^ ^]^ Copyright 2024, American Chemical Society.

PL‐based biosensing offers high sensitivity, excellent spatiotemporal resolution, and versatile detection capabilities. Among PL‐based systems, FRET‐based strategies have demonstrated significant advances in cancer diagnosis.^[^ ^60^, ^106^, ^195^, ^196^ ^]^ For example, Luo et al. developed an excitation/emission‐enhanced heterostructure photonic crystal array synergizing with entropy‐driven circuit and donor‐donor‐acceptor FRET for ultrasensitive detection of ctDNA, achieving a limit of detection (LOD) of 12.9 fm and mutation frequency analysis as low as 0.01%.^[^ ^197^ ^]^ He et al. reported a UCNP‐perovskite quantum dot probe that achieved 70.6% energy transfer efficiency under 980 nm excitation, enabling sensitive detection of cancer biomarker miRNA‐155 with a LOD of 73.5 pm.^[^ ^198^ ^]^ Similarly, Wang et al. employed a MnO_2_ nanosheet‐mediated FRET strategy for simultaneous and ratiometric monitoring of miRNA‐373 and miRNA‐96 in living cell.^[^ ^199^ ^]^ To amplify detection signals, Jiang et al. reported a dual self‐protected DNAzyme‐based 3D DNA walker (dSPD walker) system for the ultrasensitive detection and imaging of microRNA in living cells.^[^ ^200^ ^]^ As shown in **Figure** **4**a, the system consists of two key components: dual self‐protected walking particles (dSPWPs) and track particles (TPs). The dSPWPs are assembled on AuNPs using three DNA strands (W1, S1, W2), while TPs are modified with Cy5‐labeled hairpin H1. This system amplified signals through two sequential steps. First, target miRNA‐221 initiated DNAzyme‐mediated cleavage, producing multiple secondary targets that simultaneously activate walking strands. Second, these activated dSPWPs traversed along TPs, cleaving substrate strands and generating fluorescence signals detected by DNA nanospheres. This design achieved rapid miRNA‐221 detection within 10 min at a LOD of 0.84 pm, performing ten times faster than conventional DNA walkers. Liu et al. developed a programmable QD nanosensor that employs three‐way junction skeleton‐mediated cascade signal amplification to achieve ultrasensitive detection of circular RNAs in clinical breast tissues with attomolar sensitivity (7.12 aM) and demonstrated single‐cell analysis capability.^[^ ^201^ ^]^ Furthermore, FRET‐based systems have also demonstrated exceptional sensitivity in detecting cancer exosomes, which are crucial biomarkers for early cancer diagnosis. Zhu et al. developed a magnetic aptamer‐sensor combining ZnCdSe/ZnS QDs with DNA‐modified AuNPs, achieving a LOD of 13 particles mL^−1^ for lung cancer exosomes.^[^ ^202^ ^]^ Zhang et al. developed a platform using cationic lipid‐polymer hybrid nanoparticles encapsulating CHA‐CRISPR‐Cas12a (CLHN‐CCC), achieving over 90% extracellular vesicles (EV) enrichment and in‐situ EV‐associated microRNA detection with a (LOD of 10 particles/µL within 30 min.^[^ ^203^ ^]^ Recent advances have led to the integration of nanosensors with microfluidic platforms for high‐throughput detection, such as the droplet microfluidic flow cytometry (Nano‐DMFC) integrated with 2D MOF‐based FRET nanosensor developed by Chen et al., which can detect multiple intracellular miRNAs in single tumor cells (Figure 4b).^[^ ^204^ ^]^ The operational mechanism involved generating single‐cell droplets through precise control of oil‐aqueous phase flow rates, followed by the injection of biofunctionalized MOF nanosensors. The MOF nanosensors labeled with various dye‐labeled oligos then enter the single cell in the droplets for detection of multiple intracellular miRNAs. When target miRNA is present, dye‐labeled single‐stranded DNA (ssDNA) on the MOF nanosheet hybridized with the target to form double‐stranded DNA (dsDNA), weakening its interaction with the MOF surface and triggering fluorescence recovery with distinct colors for different miRNAs in single cells. This system demonstrated remarkable sensitivity, successfully detecting dual miRNAs at single‐cell resolution and identifying ten positive tumor cells among 10 000 negative epithelial cells in biomimetic serum samples.

Beyond cancer biomarker detection, FRET‐based systems have been extended to smart responsive platforms that respond to tumor microenvironmental changes.^[^ ^205^ ^]^ Zhao et al. developed enzyme‐activated nanoflares with ATP‐specific aptamers conjugated to AuNPs, which utilized overexpressed APE1 in cancer cells to cleave an engineered AP site, restoring ATP‐binding ability and triggering fluorescence enhancement for cancer‐specific biosensing and imaging.^[^ ^206^ ^]^ In addition, monitoring immune cell activity in cancer immunotherapy has emerged as a new frontier. Huang et al. developed a dual‐emitting AIE nanoprobe to monitor T lymphocyte differentiation through intracellular pH changes (Figure 4c).^[^ ^90^ ^]^ The nanoprobe combined pH‐sensitive TPE‐AMC and pH‐insensitive TPE‐TCF in a CD8‐targeting F127 polymer shell, forming a donor–acceptor pair for sensitive intracellular pH detection. As pH decreased, TPE‐AMC's protonation enhanced both its emission and FRET to TPE‐TCF, enabling dual‐emitting enhancement analysis with distinctly sensitive narrow range of intracellular pH value between pH 6.0–7.4. The system successfully distinguished naïve CD^8+^ T cells (pH ∼7.26) from activated ones (pH ∼6.49), advancing both cancer diagnostics and immunotherapy monitoring capabilities.

##### SERS‐Based Biosensing

Plasmon‐enhanced SERS biosensors based on noble metal nanostructures have emerged as a powerful analytical tool for cancer biomarker detection. This technique employs two primary detection strategies: direct (label‐free) detection and indirect (SERS tag‐based) detection.^[^ ^207^ ^]^

**Figure 5:**
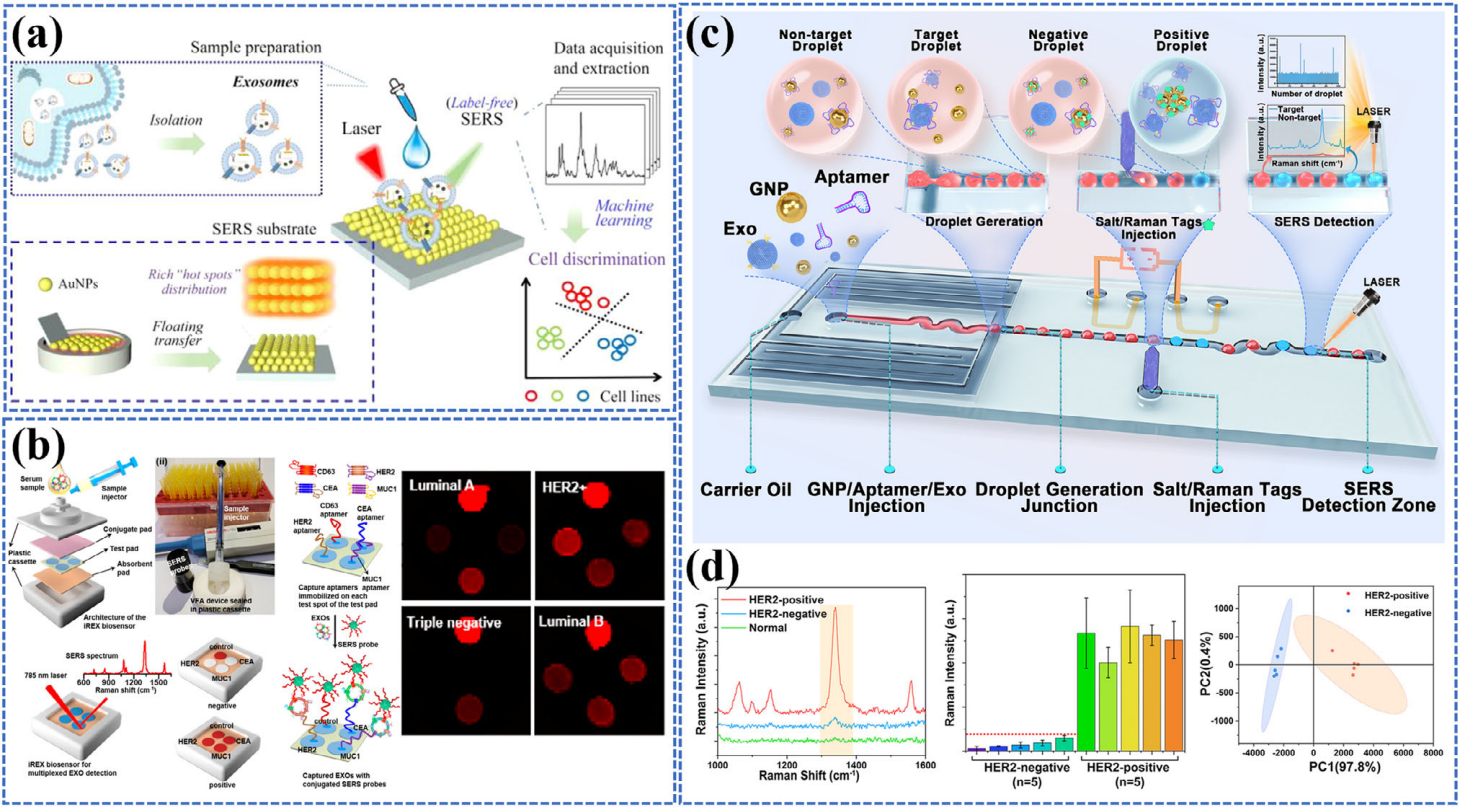
Photo‐triggered SERS‐based biosensors for cancer diagnosis. a) Schematic diagram of label‐free SERS detection and accurate fuzzy discrimination of exosomes derived from different cell lines. Reproduced with permission.^[^ ^212^ ^]^ Copyright 2023, American Chemical Society. b) Operating principle of the iREX biosensor and SERS images of test spots with additional samples. Reproduced with permission.^[^ ^225^ ^]^ Copyright 2023, American Chemical Society. c) Illustration of the process of SERS‐based droplet microfluidic platform for detecting HER2‐positive exosomes. d) SERS‐based microdroplet platform for the detection of HER2‐positive exosomes extracted from plasma in the clinic. c‐d) Reproduced with permission.^[^ ^226^ ^]^ Copyright 2024, American Chemical Society.

In the direct detection approach, cancer biomarkers can interact directly with plasmonic surfaces to generate characteristic Raman fingerprint spectra, enabling label‐free identification and quantification.^[^ ^208^ ^]^ This approach is particularly valuable for studying native cancer biomarkers, though it requires targets with sufficient Raman cross‐sections.^[^ ^209^ ^]^ Artificial intelligence (AI)‐assisted analysis is also necessary to derive characteristic features from SERS spectra for accurate diagnostics. Early developments have demonstrated promising clinical applications, exemplified by Dong et al.’s beehive‐inspired Au‐coated TiO_2_ microporous SERS probes, which enable cancer diagnosis by detecting phosphoproteins in plasma exosomes, showing two‐fold higher signal intensity in cancer patients compared with healthy people.^[^ ^210^ ^]^ Phyo et al. developed a label‐free SERS platform using 3D‐stacked Ag nanowires on a glass fiber filter for urinary metabolomic analysis.^[^ ^211^ ^]^ By combining this platform with calcium ion pretreatment and multivariate statistical analysis, they successfully distinguished between normal controls, pancreatic cancer, and prostate cancer. Recently, the integration of machine learning algorithms has dramatically enhanced the capabilities of label‐free SERS detection. Diao et al. reported a 3D plasmonic AuNPs nanofilm enriched with “hot spots” combined with machine learning analysis for exosome‐based cancer diagnosis.^[^ ^212^ ^]^ They successfully distinguished normal (H8) and cancer (HeLa, MCF‐7) cell‐derived exosomes with an accuracy of 91.1% while demonstrating a prediction accuracy of 93.3% in clinical serum samples and enabling real‐time monitoring of chemotherapy effects through dynamic exosome analysis (**Figure** **5**a). Similarly, Xie et al. created a deep learning‐assisted label‐free SERS strategy that achieved 100% prediction accuracy in distinguishing cancer subtypes via an artificial neural network trained on the spectral patterns of exosomes derived from cancer cell lines.^[^ ^213^ ^]^ These advancements highlight the evolution of label‐free SERS from simple spectroscopic analysis to AI‐integrated diagnostic platforms capable of highly accurate disease detection and monitoring.

The indirect detection approach employs engineered SERS tags consisting of a plasmonic core (Au or Ag nanoparticles), Raman reporter molecules, a protective shell, and a targeting ligand.^[^ ^214^ ^]^ Typically, the plasmonic core amplifies Raman scattering through LSPR, while Raman reporter molecules generate specific spectral patterns for target identification.^[^ ^215^ ^]^ The protective shell, functionalized with targeting ligands (antibodies, aptamers, or peptides), ensures selective binding to different targets.^[^ ^216^ ^]^ The detection process relies on signal enhancement when SERS tags bind to targets, bringing reporter molecules close to the plasmonic core. This proximity effect creates an intense Raman signal that indicates target presence. Furthermore, multiple targets can be detected in a single measurement using different reporter molecules with unique spectral signatures.^[^ ^217^ ^]^ The sensitivity of this approach is remarkable, often achieving detection limits down to the single‐molecule level, making it a powerful tool for cancer diagnostics. For example, Canning et al. achieved excellent sensitivity with LODs of 6.8 and 16.7 zmol for multiple miRNAs (miR21 and miR221) detection using silver‐coated gold nanostars, respectively.^[^ ^218^ ^]^ Zhai et al. developed a dual‐mode biosensor combining SERS and electrochemical detection, using a multifunctional MoS_2_ probe on a silver nanorod array electrode, to detect the gastric cancer biomarker miR‐106a in human serum samples and achieved femtomolar sensitivity (67.44 fm SERS, 248.01 fm electrochemical).^[^ ^219^ ^]^ For protein detection, Lu et al. developed a molecularly imprinted polymer (MIP)‐SERS biosensor for colorectal cancer biomarker NDKB protein detection in serum, achieving a remarkable detection limit of 0.82 pg mL^−1^.^[^ ^220^ ^]^ For CTC detection, Li et al. developed an aptamer‐modified magnetic bead system combined with rolling circle amplification to achieve detection limits of 2 cells mL^−1^.^[^ ^221^ ^]^ Zhang et al. advanced CTC detection by combining dual‐modal SERS probes (Au‐4MBA‐rBSA‐FA and iron oxide (IO)‐AR‐rBSA‐FA) with machine learning.^[^ ^222^ ^]^ The probes enable magnetic separation and Raman signal generation, with folic acid (FA) targeting CTCs and rBSA providing stability. Raman spectra collected from CTCs were analyzed using principal component analysis and Random Forest algorithms, achieving a LOD of 2 cells mL^−1^, a 98% detection rate, and 90% classification accuracy for tumor subtypes.

Recent innovations in exosome detection have further expanded SERS capabilities. Zhang et al.’s ratiometric SERS biosensor utilizing Au@Ag/GO substrate for breast cancer exosome detection achieved a LOD as low as 150 particles mL^−1^ through a dual‐aptamer system.^[^ ^223^ ^]^ Ma et al. developed a highly sensitive SERS‐based immunosensor using Cu_2_O–CuO@Ag nanowire substrates and anti‐EpCAM‐conjugated Au@RhB nanotags for the efficient capture and detection of prostate cancer exosomes, achieving a remarkable LOD of 89 particles mL^−1^.^[^ ^224^ ^]^ Su et al. further advanced the technology by integrating AuSt nanoparticle‐based SERS platform with a paper‐based vertical flow design for simultaneous quantification of three exosomal proteins (MUC1, HER2, and CEA) in breast cancer.^[^ ^225^ ^]^ As shown in Figure 5b, the test pad features four distinct wax‐printed spots, each containing different capture aptamers for MUC1, HER2, CEA, and a CD63 control. The device operates through a sequential process where exosomes first bind to capture aptamers on the test spots, followed by SERS probes forming sandwich complexes with the captured exosomes. Through SERS imaging analysis, the LOD was determined to be 1.0–2.9 × 10^7^ particles mL^−1^. Most recently, Ho et al. developed a SERS‐based droplet microfluidic platform for sensitive and high‐throughput detection of breast cancer exosomes.^[^ ^226^ ^]^ As shown in Figure 5c, a single droplet containing exosomes, aptamers, and AuNPs was first generated and injected with salt solution/Raman tags. In the absence of target exosomes, the aptamer layer covering the AuNPs surface can prevent the aggregation of AuNPs under high salt conditions so that the SERS signal remains unchanged. In the presence of target exosomes, the HER2 aptamer will detach from the AuNPs surface because the aptamer has a higher affinity with the target exosomes, resulting in the aggregation of AuNPs and the generation of “hot spots” under high salt conditions, thereby enhancing the SERS signal. The platform demonstrated high sensitivity with an LOD of 4.5 log_10_ particles mL^−1^ in biomimetic samples and proved effective in analyzing clinical plasma samples from breast cancer patients (Figure 5d). The increasing incorporation of paper‐based flow test strips, microfluidic systems, or machine learning algorithms suggests that SERS‐based cancer diagnostics will become more precise, automated, and clinically applicable in the future, highlighting its growing importance in cancer diagnostics.^[^ ^227^ ^]^

##### PEC Biosensing

**Figure 6:**
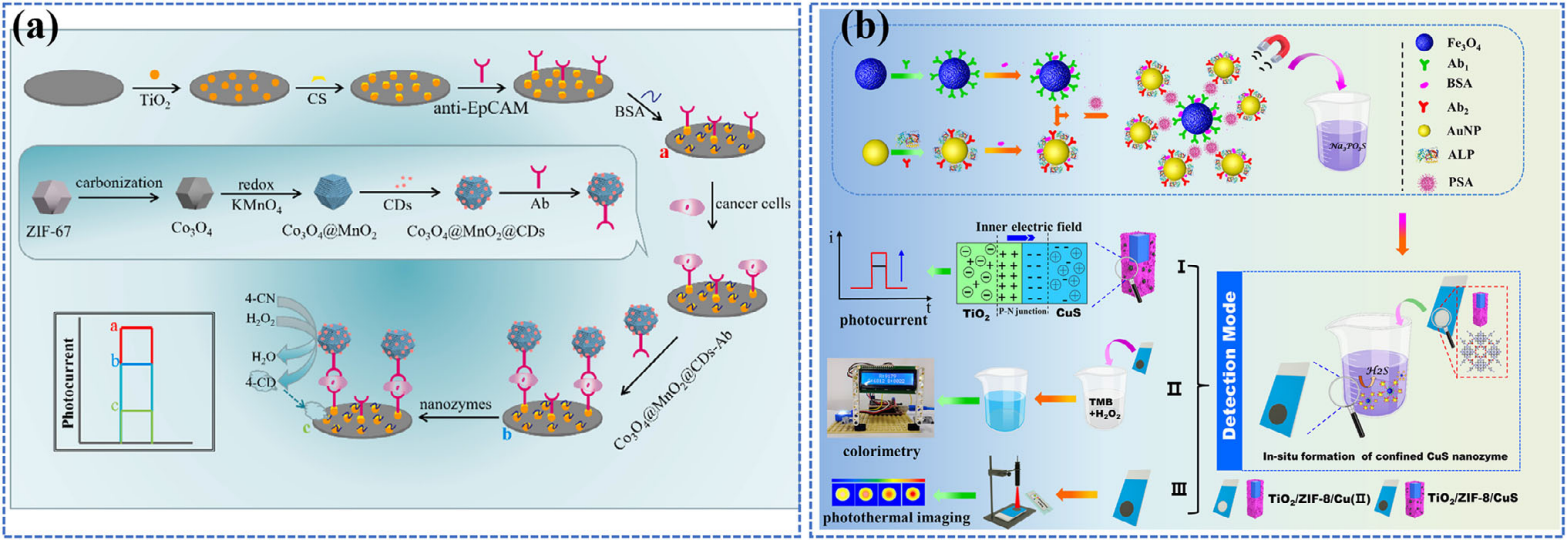
Photo‐triggered PEC biosensors for cancer diagnosis. a) Schematic illustration of the PEC biosensor for the detection of cancer cells based on Co_3_O_4_@MnO_2_@CDs polyhedron with enhanced nanozymes activity. Reproduced with permission.^[^ ^235^ ^]^ Copyright 2023, Elsevier. b) Schematic illustration of the proposed multi‐mode PEC immunoassays‐based on TiO_2_/ZIF‐8/Cu(II). Reproduced with permission.^[^ ^237^ ^]^ Copyright 2024, American Chemical Society.

PEC biosensing platforms represent an innovative integration of photochemical and electrochemical processes, offering unique advantages for cancer biomarker detection. These systems utilize semiconductor nanomaterials or hybrid nanostructures that generate photocurrent under light illumination, with target biomarkers modulating the photocurrent through various mechanisms.^[^ ^228^ ^]^ PEC biosensors excel in delivering low background signals, high sensitivity, and straightforward operation under mild conditions.^[^ ^229^ ^]^ Li et al. developed a BiVO_4_/Ag_2_S heterojunction‐based PEC biosensor with Exo III‐assisted silver nanoclusters amplification, achieving a 112‐fold photocurrent enhancement and a LOD of 0.42 fm for tumor suppressor gene P53.^[^ ^230^ ^]^ Hu et al. developed a PEC aptasensor based on size‐controlled AuNPs/GaN Schottky junction for sensitive CA125 detection (LOD 0.3 U mL^−1^).^[^ ^231^ ^]^ They further enhanced this aptasensor by sputtering a 30 nm Au film onto n‐type GaN and conjugating it with multilayer MoS₂ to provide a sensitive platform for alpha‐fetoprotein (AFP) detection, achieving excellent analytical performance with a LOD of 0.3 ng mL^−1^ and recoveries of 85.2–91.7%.^[^ ^232^ ^]^ Later, significant developments in detection strategies have emerged. Xu et al. developed a Cd:Sb_2_S_3_‐sensitized La_2_Ti_2_O_7_ heterojunction‐based PEC aptasensor employing a novel “on‐off‐on” signal strategy for ultrasensitive prostate‐specific antigen (PSA) detection with a LOD of 4.300 fg mL^−1^.^[^ ^233^ ^]^ Zhong et al. established a dual‐signal PEC immunosensor using MIL‐101(Cr) MOF with Ag_2_S, enabling sensitive detection of breast cancer markers CEA and CA15‐3 at remarkably low LOD of 0.0023 ng mL^−1^ and 0.014 U mL^−1^ respectively.^[^ ^234^ ^]^ Liu et al. established a PEC biosensor based on multifunctional Co_3_O_4_@MnO_2_@CDs polyhedron nanozymes, combining photocurrent quenching and catalytic precipitation effects to achieve sensitive detection of A549 cancer cells with a LOD of 11 cells mL^−1^ (**Figure** **6**a).^[^ ^235^ ^]^ Luo et al. developed a hexagonal carbon nitride tube‐based PEC biosensor for detecting CTCs, which achieved highly sensitive quantitative detection (LOD 1 cell mL^−1^, range 3–3000 cells mL^−1^) by combining specific capture, steric hindrance, and competitive light absorption mechanisms.^[^ ^236^ ^]^

Recently, the development of PEC biosensors has led to multimodal detection systems. Meng et al. developed an integrated TiO_2_/ZIF‐8/Cu(II) chip platform combining PEC, colorimetric, and photothermal detection modes, achieving detection limits of 0.16‐0.41 pg mL^−1^ for PSA across different modalities.^[^ ^237^ ^]^ The system employed alkaline phosphatase‐labeled magnetic probes and in‐situ formation of CuS nanostructures within ZIF‐8 confinement, creating p‐n heterojunctions that enhance photocurrent generation while simultaneously enabling peroxidase‐mimicking activity and photothermal detection (Figure 6b). Moving toward point‐of‐care applications, Zeng et al. developed a smartphone‐based portable PEC immunoassay system for point‐of‐care HER2 detection.^[^ ^238^ ^]^ Utilizing hierarchical Co_9_S_8_@ZnIn_2_S_4_ heterostructures and alkaline phosphatase‐catalyzed ascorbic acid generation for signal amplification, they achieved clinical‐grade sensitivity (3.5 pg mL^−1^) across a broad detection range 0.01‐10 ng mL^−1^, while offering the advantages such as wireless connectivity, real‐time data processing via a custom smartphone app, and the potential for widespread deployment in resource‐limited settings.

#### 3.1.2 Bioimaging

Photo‐triggered bioimaging systems have emerged as an advanced diagnostic tool in oncology, employing light‐responsive nanomaterials to achieve high‐resolution visualization and real‐time disease monitoring. These systems can be fundamentally categorized by their underlying mechanisms, including PL imaging, SERS imaging, and PAI.

##### PL Imaging

**Figure 7:**
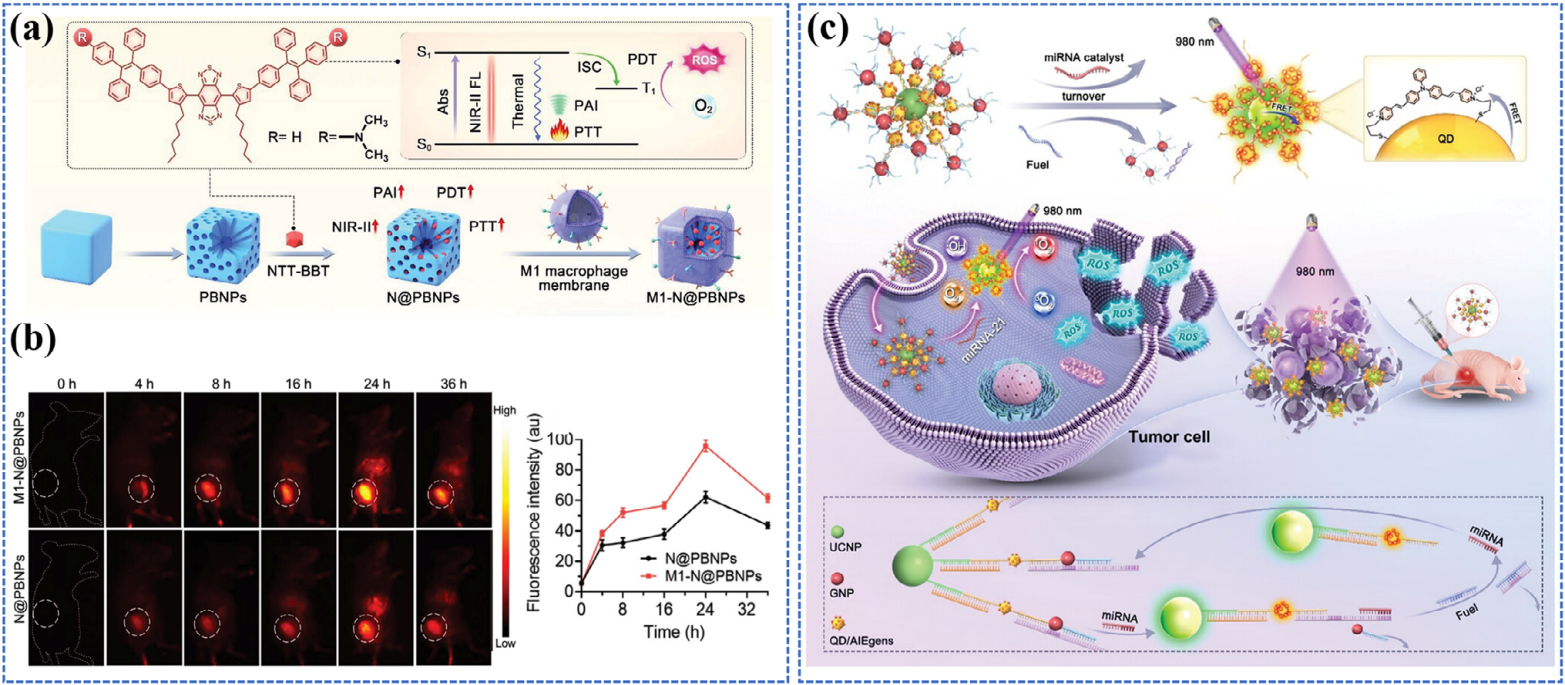
Photo‐triggered PL imaging systems for cancer diagnosis. a) Schematic illustration showing the integration of NIR AIEgen with mesoporous PB nanocatalyzer. b) In vivo NIR‐II FL imaging and the corresponding intensities of orthotopic breast tumor‐bearing mice at various time points after intravenous injection of N@PBNPs or M1‐N@PBNPs. a,b) Reproduced with permission.^[^ ^242^ ^]^ Copyright 2024, Wiley‐VCH. c) Schematic illustration of a hierarchical UCNP‐QDs‐GNPs nanoprobe and its application in catalytic microRNA imaging and image‐guided therapy of living cancer cells and tumor tissues. Reproduced with permission.^[^ ^107^ ^]^ Copyright 2024, Wiley‐VCH.

The development of PL imaging systems has progressed from standalone diagnostic tools to integral components of therapeutic strategies, enabling precise imaging and effective cancer treatments. PL imaging systems leverage both downconversion and upconversion processes to enhance sensitivity and therapeutic precision. Downconversion bioimaging excels in high‐sensitivity diagnostics and imaging, making it ideal for molecular specificity and tumor visualization.^[^ ^63^ ^]^ For example, Li et al. developed a QDs‐mediated FRET nanosensor with multiple primer generation rolling circle amplification (MPG‐RCA) for precise single‐molecule imaging of single‐nucleotide polymorphisms (SNP).^[^ ^239^ ^]^ Liu et al. created PDAD@F68‐HA‐DOX nanoprobes that combine pH‐responsive fluorescence and FRET‐based imaging for precise tumor visualization, while utilizing hyaluronic acid for CD44‐mediated targeting and HAase‐triggered DOX release to achieve selective tumor cell apoptosis with minimal impact on normal tissues.^[^ ^240^ ^]^ Feng et al. developed an AIEgens‐based photosensitizer (CBTM) that co‐assembled with the therapeutic peptide (anti‐tumor tyroservaltide, YSV) to form self‐delivering nanoparticles, overcoming the traditional aggregation‐induced quenching effect while providing strong NIR‐I fluorescence for imaging guidance and efficient ROS generation for PDT.^[^ ^241^ ^]^ Li et al. integrated dimethylamine‐substituted AIEgens into porous Prussian blue nanoparticles (PBNPs) and coated M1 macrophage membrane for FLI/PAI‐guided cancer immunotherapy.^[^ ^242^ ^]^ As shown in **Figure** **7**a, AIE genes modified with dimethylamine exhibited better NIR photothermal conversion and ROS generation, while their incorporation into PB nanoparticles effectively restricted molecular motion, thereby enhancing fluorescence brightness and PDT efficiency. PB catalyzes tumor‐overexpressed H_2_O_2_ into oxygen, alleviating tumor hypoxia to boost PDT, while its NIR absorption enhances both PAI and PTT. M1 macrophage membrane coating enabled tumor‐specific targeting and improved therapeutic efficacy by enhancing accumulation and immune response at tumor sites. In vivo imaging results show excellent tumor visualization by dual‐mode NIR‐II FLI/PAI under 730 nm light (1.0 W cm^−2^), with signal intensity peaking 24 h after injection and membrane‐coated nanoparticles exhibiting 1.5‐fold higher fluorescence than uncoated nanoparticles (Figure 7b).

In contrast, upconversion bioimaging utilizes NIR excitation for deep‐tissue imaging and therapy, enabling superior penetration and reduced background interference.^[^ ^243^ ^]^ Liu et al. developed NIR light‐controlled DNA nanodevices combining UCNPs with photolabile DNA duplexes for targeted drug delivery and imaging.^[^ ^244^ ^]^ Zhou et al. developed FNPs‐Gd nanocomposites by integrating ferrocenylseleno compounds with Gd^3+^‐doped UCNPs to achieve enhanced T_1_‐T_2_ dual‐mode MRI contrast through NIR‐triggered Fenton reactions for cancer therapy.^[^ ^100^ ^]^ Most recently, UCNPs have been used as NIR‐responsive optical transducers capable of penetrating deep tissues through biological windows. Sun et al. constructed a hierarchical nanoprobe (UCNP‐QDs‐GNPs) through DNA‐programmed assembly for simultaneous miRNA detection and image‐guided PDT (Figure 7c).^[^ ^107^ ^]^ The PL of this system was initially quenched through proximity‐dependent FRET to the GNPs via DNA hybridization. Upon encountering miRNA‐21, catalytic disassembly of GNPs occurs via entropy‐driven DNA strand displacement, recovering QD/UCNP fluorescence for amplified imaging (LOD 9.89 pm). Concurrently, NIR light (980 nm, 6.6 mW cm^−2^) harvested by UCNPs transfers energy to QDs, which photosensitize surface‐bound AIE molecules (TdVP‐T), boosting ROS generation. The AIE photosensitizers exhibited 77‐fold higher ROS efficiency than Rose Bengal under white light (2 mW cm^−2^) and maintained efficacy under NIR excitation through 3 mm of tissue, enabling deep‐tumor therapy. In vivo, NIR‐driven PDT suppressed tumor growth in mice with negligible systemic toxicity, offering a promising theranostic platform for early cancer diagnosis and precision treatment of deep malignancies. Similarly, Song et al. demonstrated another PL cascade‐mediated therapeutic approach using a DNA/UCNP complex that integrated CRISPR‐Cas9, hemin, and protoporphyrin (PP) for enhanced PDT.^[^ ^108^ ^]^ Their system utilized UCNP‐mediated conversion of 980 nm excitation to 409 nm emission, specifically matched to the absorption spectrum of PP, enabling efficient singlet oxygen generation for therapeutic effects.

##### SERS Imaging

SERS imaging combines the molecular fingerprinting of Raman spectroscopy with plasmon enhancement, bringing new approaches to cancer diagnosis. This technique offers distinct advantages over FLI, including ultra‐sensitivity, molecular specificity, multiplexing capability, superior biocompatibility, and resistance to photobleaching.^[^ ^163^ ^]^ SERS‐active nanoparticles, when functionalized with cancer‐specific targeting ligands, effectively accumulate in tumors through both active molecular recognition and passive EPR effect, enabling detailed molecular mapping of the tumor microenvironment.^[^ ^245^ ^]^

Recent advances in SERS imaging‐based cancer detection have demonstrated remarkable capabilities across multiple biological scales, such as cellular imaging,^[^ ^246^, ^247^, ^248^ ^]^ ex vivo tissue analysis,^[^ ^249^, ^250^, ^251^, ^252^ ^]^ and in vivo tumor detection.^[^ ^253^, ^254^, ^255^ ^]^ At the cellular level, Zhong et al. developed a ratio‐type SERS nanoprobe for MMP‐2 imaging in cancer cell subtypes, utilizing AuNP as a SERS enhancer with rhodamine B‐labeled peptides and 2‐naphthalenethiol as an internal standard.^[^ ^246^ ^]^ Yue et al. introduced a DNA tetrahedron‐mediated branched catalytic hairpin assembly (DTM‐bCHA) strategy for ultrasensitive detection and in situ imaging of miRNA‐21 in living cells.^[^ ^247^ ^]^ The method dynamically fabricated hyperbranched 3D DNA structures that assembled with Raman reporter DTNB‐functionalized AuNPs (DTNB@AuNPs) to generate SERS hot spots, enabling intensified signal detection. Choi et al. developed SERS nanotags based on Ag‐encapsulated Au (Ag–Au) hollow nanospheres with three Raman reporters (MGITC, RBITC, DTDC) positioned in nanogaps to enhance the electromagnetic field.^[^ ^248^ ^]^ Through conjugation with specific antibodies, these nanotags enabled simultaneous and quantitative detection of multiple breast cancer biomarkers (EpCAM, ErbB2, and CD44) via SERS mapping techniques. Building upon this foundation, Qiu et al. further advanced this approach with Au@Ag core‐shell nanoparticles with double‐layer Raman reporters for triple‐biomarker (EGFR, ErbB2, IGF1) detection.^[^ ^249^ ^]^ In vivo investigations using xenotransplanted tumor models validated the efficacy of this platform in monitoring therapeutic responses to tamoxifen treatment and surgical intervention.

**Figure 8:**
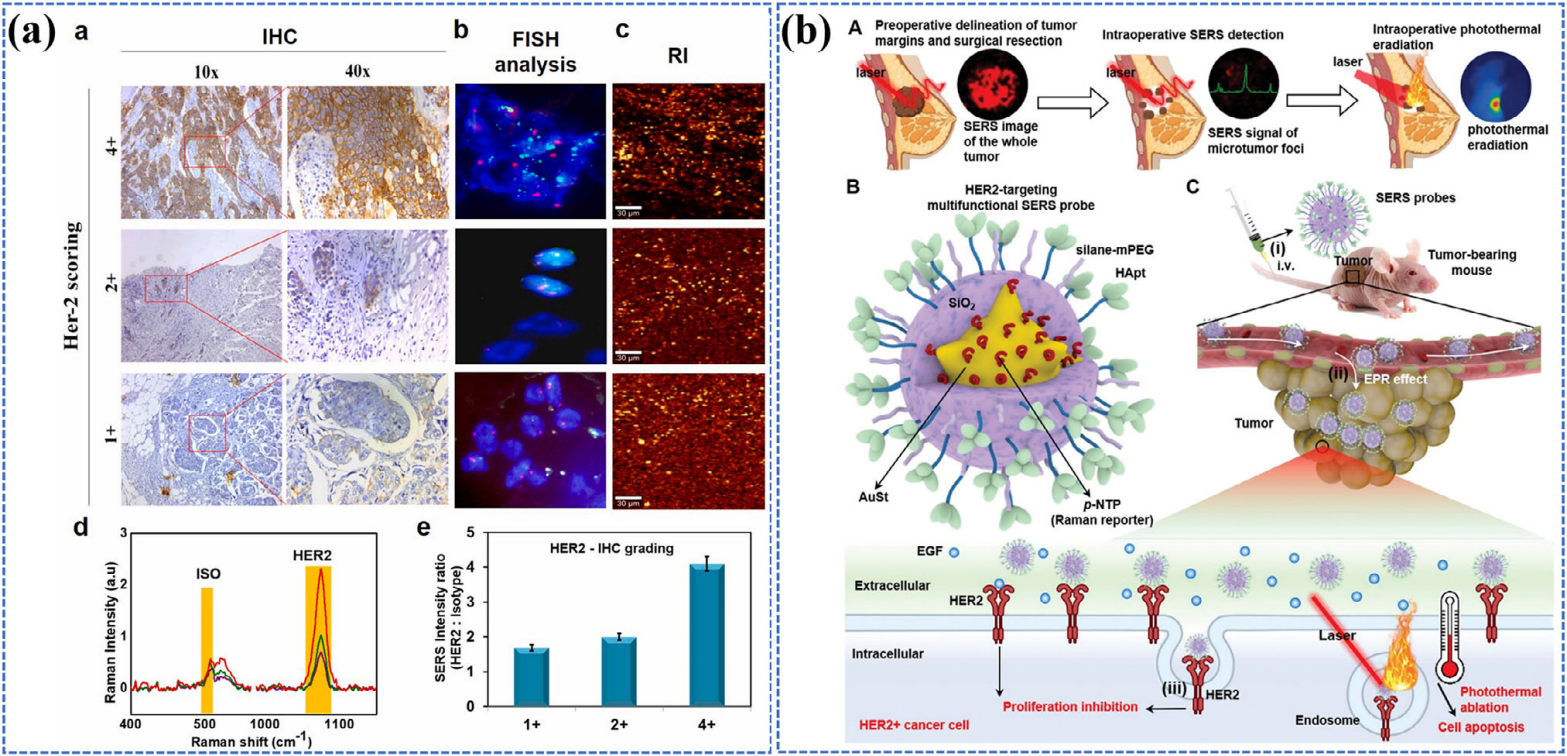
Photo‐triggered SERS imaging systems for cancer diagnosis. a) SERS analysis showing HER2 grading in HER2+ tissue using HER2‐targeted AuNP@MBA@anti‐HER2 and Isotype‐targeted AuNP@DTTC@anti‐isotype nanotags. Reproduced with permission.^[^ ^250^ ^]^ Copyright 2023, Elsevier. b) Schematic diagram of SERS imaging‐guided HER2+ breast‐conserving surgery and intraoperative real‐time elimination of microscopic tumor lesions. Reproduced with permission.^[^ ^253^ ^]^ Copyright 2024, Wiley‐VCH.

Based on these results, tissue‐level SERS imaging applications have emerged to address clinical diagnostic needs. Murali et al. reported a multiplexed detection system based on Raman‐label SERS (RL‐SERS) nanotags for simultaneous analysis of three critical breast cancer biomarkers (ER, PR, and HER2) in formalin‐fixed paraffin‐embedded tissue samples.^[^ ^250^ ^]^ The system demonstrated excellent diagnostic capabilities, with sensitivity and specificity of 95% and 92% for single biomarker detection, 88% and 85% for dual detection, and 75% and 67% for triple detection. A particularly important advance is the platform's ability to perform semi‐quantitative HER2 grading (4+/2+/1+) by ratiometric SERS intensity analysis, which showed excellent correlation with traditional fluorescence in situ hybridization (FISH) results (**Figure** **8**a). Besides, this system enabled rapid large‐area SERS imaging, capable of analyzing areas between 0.5 and 5 mm^2^ within 45 min, while employing ratiometric analysis to minimize false results. The tissue‐level analysis was also complemented by Kobu et al.’s infrared laser‐scanning platform, which effectively differentiated between ductal carcinoma in situ and invasive breast cancers in fresh‐frozen needle‐biopsied samples.^[^ ^251^ ^]^ Furthermore, Li et al. applied a volume‐active SERS (VASERS) platform with correlation network analysis to predict optimal immune checkpoint inhibitor combinations for cancer immunotherapy.^[^ ^252^ ^]^ The developed VASERS platform, utilizing 3D electromagnetic hotspots with randomly arranged Raman molecules, achieves 32 resolvable colors in the Raman‐silent region – the highest reported to date. When applied to breast cancer tissue biopsies, the system allows for simultaneous imaging of immune checkpoints in ten colors, revealing heterogeneous expression patterns across different tumor subtypes. Through correlation network analysis of this high‐throughput data, the study identifies promising inhibitor combinations for synergistic immunotherapy, offering new directions for cancer treatment strategies.

These advances have enabled in vivo SERS bioimaging applications, where Wen et al. engineered a targeted SERS imaging platform for intraoperative guidance and microscopic tumor elimination to improve breast‐conserving surgery outcomes.^[^ ^253^ ^]^ The platform utilized gold nanostar‐based probes (AuSt cores) encapsulated with Raman reporters and silica shells, functionalized with HER2‐targeting nucleic acid aptamers for specific targeting of HER2^+^ breast cancer (Figure 8b). Under 808 nm irradiation at 1.0 W cm^−2^ for 5 min, these SERS probes achieved ultrahigh detection sensitivity, enabling precise visualization of tumor margins and microscopic foci in real time. The platform demonstrated clinical potential through in vivo studies, exhibiting prolonged tumor retention (>24 h post‐injection) with high target‐to‐background contrast ratios. The plasmonic AuSt cores enabled efficient photothermal ablation of tumors, resulting in complete eradication of HER2^+^ breast tumors and 100% tumor‐free survival in preclinical models with no local recurrence. Yu et al. realized five‐plex ratiometric SERS imaging based on gold multicore‐NIR Raman dyes‐silica shell SERSnanoparticle oligomers for non‐invasive monitoring of multiple tumor biomarkers in living mice.^[^ ^254^ ^]^ This system surpassed conventional three‐channel imaging limitations, demonstrating high accuracy (Pearson correlation ≥ 0.998) and low spectral deconvolution error (21.8%), with successful visualization of nanoparticles at just 4% of the administered dose. The progression to in vivo SERS imaging was further advanced by Li et al.’s implementation of porous cubic AuAg alloy nanoshells for precise visualization of both macro‐ and micro‐tumors in living mice, particularly effective in the NIR‐II window.^[^ ^255^ ^]^

##### PAI

PAI has emerged as a powerful, non‐ionizing, and noninvasive diagnostic technique for cancer due to its ability to combine the high spatial resolution of ultrasound imaging with the rich molecular information provided by optical imaging. This integration enables real‐time visualization with deep tissue penetration while maintaining the ability to detect cancer‐specific biomarkers, making it an invaluable tool for early tumor detection, growth monitoring, and treatment guidance.

**Figure 9:**
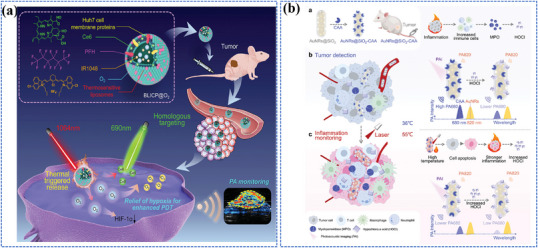
Photo‐triggered PAI systems for cancer diagnosis. a) Schematic illustration of the NIR‐II PAI guided‐controllable oxygen supply of biomimetic nanoparticles for highly specific HCC photodynamic therapy. Reproduced with permission.^[^ ^259^ ^]^ Copyright 2023, Wiley‐VCH. b) Schematic illustration of the HOCl‐responsive theranostic PA nanoprobe, AuNRs@SiO2‐CAA: a) Synthesis route of AuNRs@SiO_2_‐CAA, and its application in b) cancer detection and c) in situ monitoring of HOCl‐driven inflammation progression during PTT treatment. Reproduced with permission.^[^ ^268^ ^]^ Copyright 2024, Wiley‐VCH.

The effectiveness of PAI critically depends on contrast agents, which have evolved through two main generations. The first generation consists of “always‐on” agents, including noble metal nanomaterials,^[^ ^256^ ^]^ transition metal sulfides,^[^ ^257^ ^]^ organic dyes/polymer,^[^ ^258^, ^259^ ^]^ and AIE nanoparticles,^[^ ^260^ ^]^ which provide concentration‐dependent constant signals through passive or active tumor accumulation. Sun et al. developed glycol‐chitosan‐coated AuNPs (GC‐AuNPs) that serve as a PA contrast agent for lymph node imaging and a delivery system for tumor antigens.^[^ ^256^ ^]^ Lorenz et al. developed a bimodal PA agent using two naphthalocyanine dyes encapsulated in 40 nm polymer nanoparticles, featuring distinct peaks at 770 and 860 nm.^[^ ^258^ ^]^ This design enabled clear separation from endogenous tissue signals, allowing the detection of ovarian cancer lesions as small as 1 mm in mice. Zeng et al. reported a NIR‐II PAI‐guided biomimetic oxygen delivery system (BLICP@O_2_) integrating multiple components within thermosensitive liposomes, including IR1048 as the NIR‐II photothermal agent, perfluorohexane (PFH) as the oxygen carrier, chlorin e6 as the photosensitizer, and cancer cell membrane coating for homologous targeting (**Figure** **9**a).^[^ ^259^ ^]^ The system operates via dual‐wavelength activation: a 1064 nm laser (1 W cm^−2^, 5 min) triggers photothermal heating to melt the liposomes, releasing oxygen from PFH to alleviate hypoxia, while a subsequent 690 nm laser (1 W cm^−2^, 5 min) activates Ce6 to enhance ROS production. Real‐time monitoring is achieved through NIR‐II PAI (1064 nm), which tracks nanoparticle accumulation and dynamically measures tumor oxygen saturation, allowing precise timing of PDT. Tian et al. developed an O_2_ self‐supplying PFOB@TA‐Fe(III)‐LOX nanoplatform (PTFL, tannic acid (TA)–Fe(III) coordination complexes‐coated perfluorooctyl bromide (PFOB) nanodroplets with lactate oxidases (LOX) loading) to achieve PAI‐guided cascade metabolic chemokinetic therapy.^[^ ^260^ ^]^ By synergistically consuming ATP and lactate while generating hydroxyl radicals, this platform achieves enhanced antitumor efficacy by simultaneously disrupting tumor metabolism and oxidative stress induction. Yan et al. reported a NIR‐II fluorophore DPBTA‐DPTQ with AIE properties, which can achieve triple‐modality imaging including NIR‐II FLI, PAI, and photothermal imaging (PTI) on a single platform for balanced phototheranostics performance.^[^ ^261^ ^]^ Although these PA agents generally exhibit high extinction coefficients and photothermal conversion efficiency, their unchanging PA intensities make them susceptible to background interference, resulting in unsatisfactory signal‐to‐noise ratios.

To address these limitations, a second generation of “turn‐on” contrast agents has been developed to respond to specific biological triggers, enabling dynamic PAI of pathological changes. These activatable agents undergo structural transformations in response to tumor microenvironment conditions, including enzymes,^[^ ^160^ ^]^ pH,^[^ ^161^, ^162^ ^]^ ROS/(reactive nitrogen species) RNS,^[^ ^262^, ^263^, ^264^ ^]^ glutathione (GSH),^[^ ^265^, ^266^ ^]^ etc., resulting in distinct spectral changes. For example, Ouyang et al. developed an enzyme‐activated nanoprobe for dual‐mode imaging of breast cancer metastases using NIR‐I/NIR‐II fluorescence and multispectral optoacoustic tomography (MSOT).^[^ ^160^ ^]^ When encountered nitroreductase (the enzyme characteristically overexpressed in hypoxic tumor environments), The Q‐NO_2_ nanoaggregates can be converted into Q‐OH and transforms the probe into an activated D‐π‐A (donor–π–acceptor) structure, which further exhibited AIE in the NIR region beyond 900 nm, thus resulting in strong fluorescence and PA signals. Gao et al. synthesized a tumor acidic microenvironment‐responsive system called LET‐5, which combined croconium dye with red blood cell membrane vesicles and then covalently bonded to PEG to form amphiphilic Croc‐PEG5K.^[^ ^162^ ^]^ Under normal physiological conditions, both the NIR fluorescence (at 820 nm) and PA signals (at 780 nm) remained in an “off” state with minimal photothermal effect. However, upon encountering the acidic tumor microenvironment, the system switched “on”, demonstrating significantly enhanced PAI signals and photothermal therapeutic efficacy. Zhou et al. engineered H_2_O_2_‐responsive silica‐encapsulated gold nanochains (AuNCs@SiO_2_) that undergo a structural transformation in the tumor microenvironment, exhibiting a red‐shift to NIR‐II region and achieving 82.2% photothermal conversion efficiency at 1064 nm.^[^ ^264^ ^]^ Beyond TME‐responsive agents, Li et al. developed a magnetically modulated PAI technique using Fe_3_O_4_@Au hybrid nanorods as active contrast agents for background‐free, high‐resolution imaging of biological tissues.^[^ ^38^ ^]^ These nanorods were synthesized through an interfacial seeded growth method, integrating plasmonic and magnetic properties. By applying external alternating magnetic fields, the PA signals of these nanorods can be reversibly and instantaneously tuned, creating periodic signal changes. A fast Fourier transform‐weighted algorithm converts these periodic changes into sharp frequency‐domain peaks, effectively removing static and random noise. This approach regenerates background‐free PA images with enhanced sensitivity and specificity, achieving a remarkable 17‐fold improvement in the signal‐to‐noise ratio. Although these advances represent significant progress in molecular PAI, their reliance on single‐wavelength PA signals makes them susceptible to environmental and instrumental variations, highlighting the need for more robust detection methods.

To solve this problem, people have developed ratiometric PA detection, which compares PA signals excited at different wavelengths to improve sensitivity.^[^ ^267^ ^]^ Zhang et al. developed a responsive theranostic nanoprobe (AuNRs@SiO_2_‐CAA) that enables simultaneous cancer detection and inflammation monitoring during PTT through ratiometric PAI of hypochlorous acid (HOCl) (Figure 9b).^[^ ^268^ ^]^ This dual‐function platform consists of gold nanorods (AuNRs) coated with mesoporous silica and covalently linked to a HOCl‐responsive molecular probe (CAA), which produces distinct PA signals at 680 nm (CAA) and 820 nm (AuNRs). Through ratiometric PA signal (PA_680_/PA_820_), where PA_680_ selectively responds to HOCl while PA_820_ remains stable, the system enabled precise tumor visualization and real‐time monitoring of inflammation during PTT with a LOD of 0.34 µm (808 nm irradiation, 1.25 W cm^−2^, 8 min). Zhang et al. engineered GSH‐responsive polymer‐peptide conjugates containing purpurin‐18 that transformed from nanoparticles to nanofibers in tumors, generating ratiometric PA signals (685/730 nm) for real‐time assembly monitoring.^[^ ^269^ ^]^ Similarly. Ren et al. introduced a dual‐responsive molecular probe (POZ‐NO) that combines ratiometric FLI with “turn‐on” PAI capabilities.^[^ ^270^ ^]^ The nanoprobe utilized NO/acid‐triggered molecular transformation in the tumor microenvironment, converting from a weak electron acceptor (benzo[c][1,2,5]thiadiazole‐5,6‐diamine) to a stronger electron acceptor (5H‐[1,2,3]triazolo [4,5‐f]‐2,1,3‐benzothiadiazole), resulting in NIR fluorescence shift from 630 to 810 nm and PAI signal activation at 700 nm. Compared with single‐wavelength responsive PA nanoprobes, ratiometric PA detection offers advantages in terms of sensitivity, accuracy, and robustness against environmental factors, making it a more advanced and reliable method for quantitative PAI and molecular detection. In addition, activatable PA probes have been developed for other detections closely related to physiological and pathological processes, such as metal ions,^[^ ^271^ ^]^ CO,^[^ ^272^ ^]^ and hypoxia.^[^ ^273^ ^]^ These studies highlight the evolution of PA contrast agents from simple passive agents to responsive systems capable of specific molecular imaging and therapy.

#### 3.1.3 Multimodality Therapy

##### PTT

PTT employs photothermal transducers to convert NIR light energy into localized heat within tumors, leading to the disruption of cellular structures and protein denaturation.^[^ ^274^ ^]^ While this approach offers promising specificity for cancer treatment, several challenges currently limit its clinical implementation. Traditional PTT typically elevates tumor temperatures to ≈50 °C to achieve cell necrosis.^[^ ^275^ ^]^ However, this high‐temperature approach presents two major drawbacks: 1) necrotic cell death releases intracellular damage‐associated molecular patterns (DAMPs) that trigger severe inflammatory responses with elevated pro‐inflammatory cytokines,^[^ ^175^ ^]^ and 2) the induction of heat shock proteins (HSPs) that can contribute to thermal resistance.^[^ ^276^ ^]^

**Figure 10:**
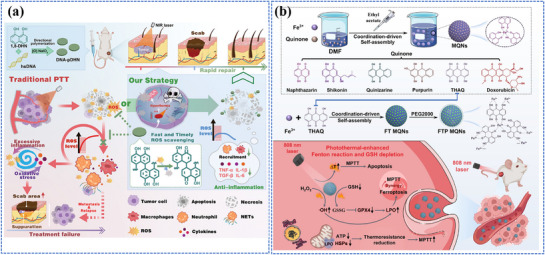
PTT for cancer. a) The preparation of DNA‐pDHN nanodisks and the designed strategy to accelerate burn tissue repair and inhibit tumor metastasis/relapse after PTT by fast scavenging of heat stress‐induced ROS. Reproduced with permission.^[^ ^277^ ^]^ Copyright 2023, Wiley‐VCH. b) Schematic illustration of the fabrication process of MQNs and the synthesis and therapeutic performance of FTP MQNs. Reproduced with permission.^[^ ^283^ ^]^ Copyright 2024, Wiley‐VCH.

To address these limitations, Wang et al. developed an innovative 2D allomelanin nanomodulator (DNA‐pDHN nanodisks) to address the inflammatory cascade after PTT (**Figure** **10**a).^[^ ^277^ ^]^ The platform utilized DNA molecules as structural templates to guide the ordered assembly of 1,8‐dihydroxynaphthalene (1,8‐DHN) oligomers through π–π stacking and hydrogen bonding interactions. This DNA‐guided assembly created nanodisks with perpendicularly oriented oligomer planes, maximizing the exposure of phenolic groups. Under 808 nm irradiation (1 W cm^−2^, 6 min), the nanodisks enabled efficient photothermal conversion, while the exposed phenolic groups rapidly neutralized heat stress‐induced ROS. Uniquely, the initial ROS scavenging and photothermal effect trigger a controlled disintegration of the nanostructure, creating a self‐amplifying process that enhances subsequent ROS elimination. Quantitative analysis demonstrated a 25‐fold reduction in ROS accumulation compared to conventional PTAs, effectively preventing the formation of a destructive inflammatory feedback loop and maintaining inflammation levels at baseline. Similarly, Wang et al. introduced a bifunctional BSA‐modified Mo‐polyoxometalate nanoagent (B‐POM NPs) that achieved efficient NIR‐II PTT with 57.2% conversion efficiency and 87% tumor inhibition, while reducing post‐treatment inflammation through ROS scavenging.^[^ ^278^ ^]^ While uncontrolled inflammation exacerbates tissue damage and tumor relapse, controlled inflammatory responses can be therapeutic. Jing et al. demonstrated this by engineering Au@MnO_2_ nanocomposites to polarize tumor‐associated macrophages to pro‐inflammatory M1 phenotypes. The system combined enhanced photothermal ablation (808 nm laser, 0.1 W cm^−2^ for 6 min in vitro; 0.3 W cm^−2^ for 10 min in vivo) with M1 macrophage polarization via TNF‐α/NF‐κB pathway activation, while also promoting TNF‐α‐enriched M1‐exosome release, effectively inhibiting bladder tumor growth.^[^ ^279^ ^]^ On the other hand, Li et al developed a comprehensive nanoplatform (MoS_2_/Fe@CPT‐11‐PEG‐iRGD) to generate low‐temperature PTT with HSP inhibition to selectively eliminate tumor cells.^[^ ^280^ ^]^ At its core, MoS_2_ nanoparticles provided controlled photothermal heating (40–48 °C), while a dopamine coating enabled the loading of both CPT‐11 and Fe^2+^ ions. The surface modification with PEG and iRGD enhanced tumor targeting specificity and circulation time. When triggered by laser irradiation and an acidic tumor environment, the released CPT‐11 increased intracellular H_2_O_2_ levels and induced cell cycle arrest, specifically during the temperature‐sensitive S‐phase. Simultaneously, Fe^2+^ ions promoted lipid ROS generation, leading to ferroptosis and the degradation of HSPs that typically confer thermal resistance to tumor cells. This approach effectively overcomes thermal resistance while maintaining the safety advantages of low‐temperature treatment, representing significant progress in addressing traditional limitations of PTT through precise temperature control and enhanced therapeutic mechanisms.

Recently, integrating ferroptosis therapy with PTT has provided an efficient approach to cancer treatment.^[^ ^281^ ^]^ PTT employs light‐absorbing agents to generate heat for tumor ablation, while ferroptosis exacerbates cytotoxicity by promoting lipid peroxidation and disrupting cellular membranes, leading to better tumor regression and prolonged survival.^[^ ^282^ ^]^ Liu et al. developed Fe‐based metal‐quinone networks (FTP‐MQNs) using a coordination‐driven self‐assembly method between Fe^3^⁺ and quinones (e.g., THAQ) to create carrier‐free nanosystems with dual Mild PTT and ferroptosis‐enhancing capabilities (Figure 10b).^[^ ^283^ ^]^ The FTP MQNs exhibited 69.3% photothermal conversion efficiency under 808 nm laser irradiation at 1 W cm^−2^ for 10 min, enabled GSH depletion and Fenton reaction‐enhanced ROS generation, and triggered ferroptosis via lipid peroxide accumulation and GPX4 downregulation. MPTT (42.8°C) induced apoptosis while photothermal‐enhanced ferroptosis disrupted HSP70‐mediated thermoresistance and reduced ATP levels, amplifying therapeutic efficacy. This work demonstrates the first mutual reinforcement of MPTT and ferroptosis, overcoming limitations of traditional high‐temperature PTT.

##### PDT

PDT serves as a minimally invasive treatment modality in various medical fields, combining three essential components: photosensitizers, light of specific wavelengths, and tissue oxygen to generate cytotoxic ROS that selectively destroy cancer cells. Despite the significant advantages of PDT, including minimal systemic toxicity, reduced long‐term side effects, and the ability for repeated treatments, its clinical application faces several critical limitations.

**Figure 11:**
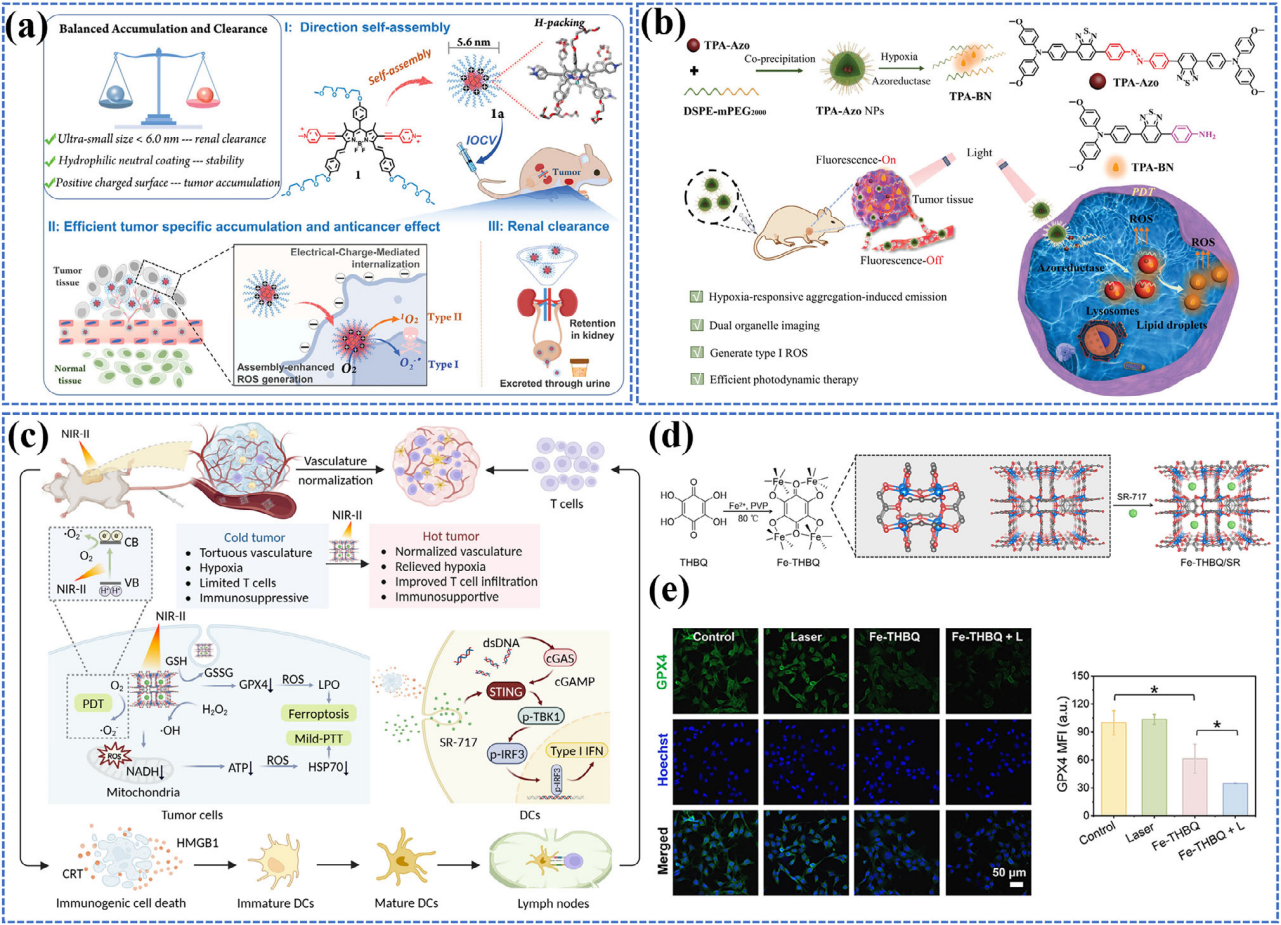
PDT for cancer. a) Schematic illustration of 1a as versatile photodynamic agent with the capability of enhanced ROS generation, tumor targeting, and renal clearance. Reproduced with permission.^[^ ^285^ ^]^ Copyright 2023, Wiley‐VCH. b) Schematic illustration of TPA‐Azo for hypoxia PDT. Reproduced with permission.^[^ ^286^ ^]^ Copyright 2022, Wiley‐VCH. c) Schematic illustration of Fe‐THBQ/SR as NIR‐II PDT and NIR‐II Mild‐PTT agent under 1064 nm laser irradiation, inducing ferroptosis, STING activation, and vasculature normalization. d) Synthetic scheme and structure of Fe‐THBQ/SR. e) Intracellular GPX4 expression in 4T1 cells under different treatments. c–e) Reproduced with permission.^[^ ^290^ ^]^ Copyright 2025, American Chemical Society.

First, a fundamental challenge in PDT development lies in the inherent conflict between tumor selectivity and systemic clearance of photosensitizers. First‐generation photosensitizers like Photofrin require 4–6 weeks for clearance, causing prolonged skin photosensitivity.^[^ ^176^ ^]^ While second and third‐generation small‐molecule photosensitizers demonstrate improved clearance profiles, they exhibit compromised tumor‐targeting efficiency.^[^ ^177^ ^]^ Although nano‐sized photosensitizers (20‐150 nm) can improve tumor targeting through the EPR effect, they suffer from mononuclear phagocyte system (MPS) uptake, leading to liver and spleen accumulation and potential long‐term toxicity.^[^ ^284^ ^]^ To address this challenge, Zhang et al. developed an ultra‐small nano‐photosensitizer (1a) through self‐assembly of boron dipyrromethene (BODIPY) derivatives with three triethylene glycol (TEG) arms and pyridinium groups (**Figure** **11**a).^[^ ^285^ ^]^ This system achieved an optimal balance with its 5.6 nm size, facilitating renal clearance, while its TEG‐modified positive surface enabled superior tumor targeting with signal‐to‐background ratios reaching 11.5. Upon 660 nm irradiation (30 mW cm^−2^), the platform demonstrated an 18.2‐fold enhancement in ROS generation compared to monomeric forms, effectively combining efficient tumor inhibition with rapid systemic clearance. Second, since traditional photosensitizers heavily rely on oxygen to generate ROS, the hypoxic nature of solid tumors often affects PDT efficacy. In this case, Tang et al. designed hypoxia‐responsive photosensitizers (TPA‐Azo) by ingeniously combining azophenyl groups with triphenylamine‐based AIE fluorophores.^[^ ^286^ ^]^ The initial state of TPA‐Azo was non‐fluorescent due to quenching by the azo group. However, when TPA‐Azo entered the hypoxic tumor environment, it was reduced by overexpressed azoreductase to generate active photosensitizer TPA‐BN, which exhibited strong fluorescence, enabling precise imaging with a high signal‐to‐noise ratio (Figure 11b). Under white light irradiation (10 mW cm^−2^), both TPA‐Azo and its reduced form TPA‐BN maintained the ability to generate type I ROS (O_2_^•−^and •OH), ensuring therapeutic efficacy under hypoxic conditions. The system also demonstrated remarkable dual‐organelle targeting capabilities, initially localizing in lysosomes before migrating to lipid droplets, thereby enabling comprehensive cellular targeting. Third, traditional photosensitizers typically require visible light activation (400–700 nm), which has poor tissue penetration due to strong absorption and scattering by biological tissues. This significantly limits PDT's effectiveness in deep‐seated tumors, as the activating light cannot reach the photosensitizers effectively. To overcome this barrier, the current solution is to use UCNP as light‐harvesting transducers, which can absorb low‐energy NIR photons and emit higher‐energy visible light that can activate nearby photosensitizers.^[^ ^108^, ^287^ ^]^ Zhang et al. developed a nanoconjugate (UCNP‐Ce6/AIEgen) that combines UCNP with Ce6 and AIEgen to achieve dual‐pathway reinforced PDT under 808 nm laser irradiation (1 W cm^−2^, 10 min).^[^ ^287^ ^]^ The system enables Ce6 activation by UCNP and AIEgen through direct lanthanide‐triplet energy transfer and FRET pathways, respectively, significantly enhancing ROS generation in deep tissues. These studies represent a significant advance toward more effective clinical PDT applications through precisely designed nanosystems that combine enhanced targeting, oxygen‐independent activation, and deep tissue treatment capabilities.

Recently, ferroptosis therapy has been effectively combined with PDT to enhance cancer treatment outcomes. For example, Yu et al. developed a bonsai‐inspired AIE photosensitizer/vermiculite nanohybrid (NSs@DCPy) by loading an AIE photosensitizer (DCPy) onto ultrathin vermiculite nanosheets via electrostatic attraction.^[^ ^288^ ^]^ This nanohybrid improved PDT efficacy by catalyzing the conversion of tumor H₂O₂ into O₂, alleviating tumor hypoxia and enabling oxygen self‐sufficiency under white light irradiation. Simultaneously, the nanohybrid induced ferroptosis through iron overloading and GSH depletion, achieving a synergistic PDT‐ferroptosis effect. Similarly, Xiong et al. developed a lipid droplet (LD)‐targeting type I photosensitizer (MNBS) that selectively accumulated in LDs due to its hydrophobicity.^[^ ^289^ ^]^ Upon 660 nm light irradiation (20 mW cm^−2^), MNBS formed H‐aggregates that enhanced O₂⁻·generation, leading to lipid peroxidation (LPO) and ferroptosis, as evidenced by GSH depletion, GPX4 downregulation, and Fe^2^⁺ elevation. This dual PDT‐ferroptosis mechanism allowed MNBS to effectively suppress tumors in both normoxic and hypoxic conditions. Furthermore, MNBS encapsulated in liposomes (MNBS‐Lip) exhibited superior tumor penetration and anti‐metastatic activity in vivo, significantly inhibiting tumor growth in 4T1 breast cancer models (IC₅₀ = 28–32 nm). To improve the tissue penetration and minimize photodamage to healthy tissues, Zhao et al. developed the first single laser‐triggered NIR‐II PDT/mild PTT‐STING activation nanosystem (Fe‐THBQ/SR) by loading a STING agonist (SR‐717) into an iron‐tetrahydroxy‐1,4‐benzoquinone (Fe‐THBQ) MOF (Figure 11c,d).^[^ ^290^ ^]^ Under 1064 nm irradiation (1 W cm^−2^), Fe‐THBQ nanosystem can generate ROS, inducing ferroptosis (via GSH/GPX4 depletion, Figure 11e) and mild‐PTT (by downregulating HSP70). Simultaneously, ROS‐mediated dsDNA leakage synergized with GSH‐responsive SR‐717 release to enhance STING pathway activation, normalizing tumor vasculature and alleviating hypoxia. This approach reversed immunosuppression, increased T‐cell infiltration, and achieved 100% survival in aggressive tumor models (4T1 and B16‐F10), highlighting the potential of combining ferroptosis, PDT, and immunotherapy in cancer treatment.

##### All‐in‐One Phototheranostics

The development of integrated multimodal theranostic platforms represents a promising trend in cancer treatment. Recent advances in photo‐triggered nanoplatforms have facilitated the rational design of all‐in‐one phototheranostic systems that integrate multiple imaging modalities (such as FLI, PAI, and SERS imaging) with diverse therapeutic approaches (such as PTT, PDT, and photo‐triggered drug release).

**Figure 12:**
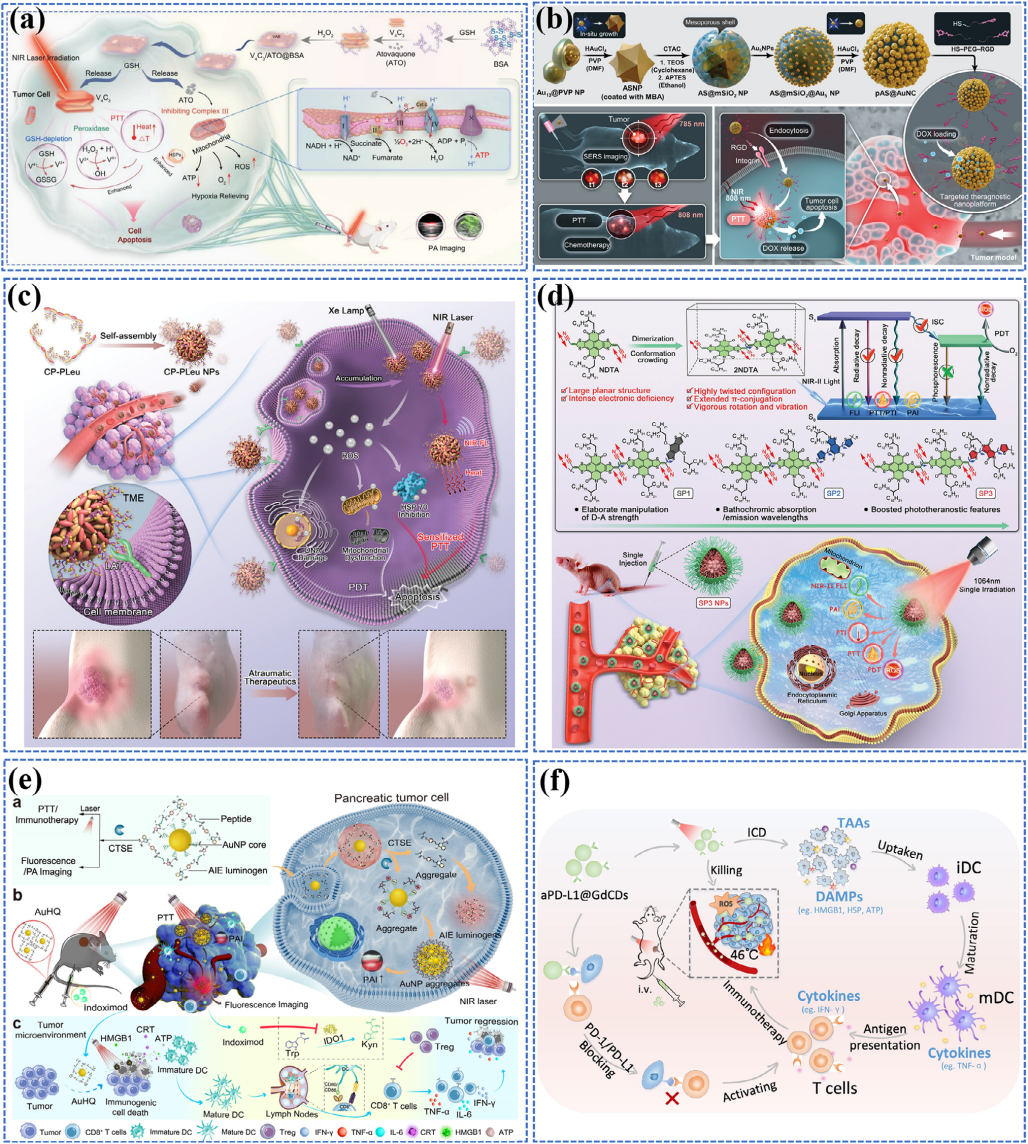
All‐in‐one phototheranostics for cancer. a) Schematic illustration of the antitumor mechanism of vanadium‐based MXene for atovaquone‐enhanced nanozyme catalytic/PTT by disrupting the tumor redox homeostasis. Reproduced with permission.^[^ ^293^ ^]^ Copyright 2023, Wiley‐VCH. b) Schematic illustration of the synthesis route and application of the multilayered mesoporous Au nanoarchitecture (RGD/DOX−pAS@AuNC) for SERS imaging‐guided synergistic therapy toward cancer. Reproduced with permission.^[^ ^34^ ^]^ Copyright 2023, Wiley‐VCH. c) Schematic illustration showing how a bioinspired photosensitizer (CP‐PLeu) performs photodynamic effects to reverse tumor thermoresistance by inhibiting the heat shock response, impairing energy metabolism, and causing DNA damage. Reproduced with permission.^[^ ^295^ ^]^ Copyright 2024, Wiley‐VCH. d) Schematic illustration of molecular design and resulting high‐performance NIR‐II excitable semiconducting polymer with AIE characteristic for FL‐PA bimodal imaging‐guided synergistic phototherapy. Reproduced with permission.^[^ ^297^ ^]^ Copyright 2024, Wiley‐VCH. e) Scheme of pancreatic cancer‐specific AuNP‐based phototheranostic modulator (AuHQ). Reproduced with permission.^[^ ^300^ ^]^ Copyright 2024, American Chemical Society. f) Schematic illustration of the synergistic phototherapy and ICB therapy via aPD‐L1@GdCDs. Reproduced with permission.^[^ ^68^ ^]^ Copyright 2024, Elsevier.

Initial developments focused on integrating single imaging modalities with multiple therapeutic mechanisms. Wang et al. used this approach with copper‐doped platinum/MOF nanostructures (PCN‐224/Pt/Cu^2+^) to combine FLI with PTT/PDT/chemodynamic therapy (CDT).^[^ ^27^ ^]^ Tian et al. constructed hybrid microbubbles (PDA–PVAMBs@GOx–TPZ) that enhanced PA signals by 6.5 times for PAI while enabling synergistic starvation therapy with low‐temperature PTT.^[^ ^39^ ^]^ Later, Zhang et al. first developed an AIPH/PDA@CuS/ZIF‐8 system that combined CuS nanoparticles with ZIF‐8 frameworks for PAI‐guided PTT/PDT/CDT,^[^ ^291^ ^]^ and then developed a more complicated ADCuSi‐FA platform that incorporated ultrasmall CuS nanodots within hollow mesoporous organosilica nanoparticles for PAI‐guided PTT/CDT/chemotherpy.^[^ ^292^ ^]^ This progress suggested a shift toward more precise modulation of the tumor microenvironment and improved nanocarrier design for synergistic cancer therapy. Zhao et al. further developed a PAI‐guided multifunctional 2D vanadium‐based MXene nanoplatform (V_4_C_3_/ATO@BSA) that combines PTT with enhanced nanozyme catalytic activity for enhanced cancer therapy (**Figure** **12**a).^[^ ^293^ ^]^ The system utilized the photothermal properties and enzyme‐like activities of V_4_C_3_ MXene nanosheets to generate ROS and deplete GSH, enhancing oxidative stress within tumors. Atovaquone (ATO), co‐encapsulated with BSA, acts as a mitochondrial respiration inhibitor, reducing ATP synthesis HSP expression, thereby overcoming cancer cell thermoresistance and amplifying the therapeutic efficacy of PTT. The results demonstrated that VAB achieves high photothermal conversion efficiency (61%) under 808 nm laser irradiation (0.6 W cm^−2^, 3 min), effectively induces cancer cell apoptosis, and significantly inhibits tumor growth.

Apart from FLI and PAI, SERS imaging‐guided therapeutic nanoplatforms represented another significant advancement. Yin et al. developed a multilayered mesoporous gold nanoarchitecture (RGD/DOX‐pAS@AuNC) that combines SERS imaging with chemotherapy and PTT.^[^ ^34^ ^]^ As shown in Figure 12b, the platform consists of mesoporous silica‐coated gold nanostars with a cyclic Arg‐Gly‐Asp (RGD)‐coated AuNCs shell, which creates multiple “hot spots” for significantly enhanced SERS signals and improved NIR photothermal conversion efficiency (85.5%). It also features a high DOX loading capacity (34.1%) and effective NIR‐triggered DOX release. In a xenograft tumor model, the nanoplatform demonstrated excellent targeting of HeLa cells, negligible cytotoxicity, and good stability both in vitro and in vivo. SERS imaging was used to determine the optimal temporal distribution of the nanoplatform at the tumor site, allowing for ultra‐effective local chemo‐PTT with 5 min of NIR irradiation (808 nm) at 0.5 W cm^−2^. Later, the phototheranostics platforms began to combine multimodal imaging with multiple treatment methods.^[^ ^43^ ^]^ Zhao et al. developed a theranostic probe embedding Gd/I‐doped carbon nanodots (Gd/I‐CDs) and DOX into mesoporous polydopamine (MPDA) (DOX@MPDA‐CDs‐CRGD, DMCR) for simultaneous triple‐modal imaging (CT/MRI/FLI) and dual‐modal therapy (chemo/PTT).^[^ ^28^ ^]^ Akhtar et al. established a dual‐targeting theranostic nanoplatform (UCNP@Tf‐RB) that combines transferrin‐mediated active targeting with UCNP for MRI/PTI‐guided synergistic PTT/PDT therapy.^[^ ^294^ ^]^ This method realized superior tumor suppression, with a tumor‐growth inhibition index (TGII) of ≈0.91, through a FRET‐mediated mechanism with minimal systemic toxicity.

Furthermore, phototheranostic platforms have increasingly emphasized NIR‐II imaging (1000–1700 nm) due to its superior tissue penetration depth and minimal photon scattering compared to traditional NIR‐I imaging. For example, Li et al. developed a bioinspired leucine‐decorated photosensitizer (CP‐PLeu) that enables efficient mild‐hyperthermia PTT (mPTT) through homogeneous tumor accumulation and photoactivated reversal of tumor thermoresistance for minimally invasive breast cancer treatment (Figure 12c).^[^ ^295^ ^]^ CP‐PLeu self‐assembled into biomimetic nanoclusters with leucine‐rich outer layers that facilitate rapid and uniform tumor accumulation through L‐type amino acid transporters (LAT) recognition. Upon dual‐light activation (Xenon lamp (0.1 W cm^−2^, 5 min) and NIR‐II laser (1064 nm, 0.5 W cm^−2^, 5 min), CP‐PLeu generated ROS to suppress HSPs (PDT) and modify the tumor microenvironment, while simultaneously providing mPTT and NIR‐II FLI capabilities. Yang et al. established ultrasmall Au44 nanocluster‐based platforms that combine NIR‐II FLI/PAI and PTT through click chemistry modification.^[^ ^296^ ^]^ The AIE behavior of this system enhanced both NIR‐II FLI sensitivity and photothermal conversion efficiency while maintaining favorable renal clearance profiles due to its ultrasmall size. Li et al. developed semiconducting polymers (SP3) with AIE characteristics, enabling NIR‐II absorption and multimodal phototheranostic functions for cancer treatment (Figure 12d).^[^ ^297^ ^]^ The polymer achieved AIE activity through the dimerization of sterically hindered NDTA acceptor units, creating large dihedral angles that prevent π‐π stacking while maintaining strong donor‐acceptor interactions. Under 1064 nm NIR‐II laser excitation (1.0 W cm^−2^, 5 min), SP3 enabled triple‐modal imaging (NIR‐II FLI, PAI, and PTI) combined with dual‐modal therapy (PTT and PDT), achieving a photothermal conversion efficiency of 35% and effective PDT through ROS generation. Similarly, Song et al. reported another all‐in‐one platform using BETT‐2 to achieve NIR‐II FL/PAI/PTI‐guided PTT/PDT for deep‐tissue cancer phototheranostics.^[^ ^298^ ^]^ In addition, Liu et al. reported a more comprehensive phototheranostic nanomedicine (Ir@PPEG‐MeEPO) that combines NIR‐II AIE Ir(III) complex with oxygen‐loaded polymer to achieve sextuple‐modal cancer theranostics via NIR‐II FLI/PAI/PTI and PTT/PDT/hydrogen gas therapy.^[^ ^299^ ^]^

While phototheranostics offers precise tumor ablation through localized photothermal and photodynamic effects, its therapeutic efficacy is often limited by incomplete tumor elimination and potential metastasis, necessitating the integration of immunotherapy to achieve systemic antitumor responses. Bian et al. established a pancreatic cancer‐specific phototheranostic modulator (AuHQ) that combines AIEgens‐tethered AuNPs with enzyme‐responsive peptides for FLI/PAI‐guided PTT/immunotherapy (Figure 12e).^[^ ^300^ ^]^ AuHQ is engineered to respond to the pancreatic tumor microenvironment by utilizing the peptide AGFSLPAGC, which is cleaved by the enzyme Cathepsin E (CTSE), prevalent in pancreatic cancer. This cleavage triggers the dual self‐assembly of AuNPs and AIE luminogens, enhancing PTT and PAI/FLI under 808 nm laser irradiation (1 W cm^−2^, 10 min). The aggregated AuNPs generate potent photothermal effects that induce immunogenic cell death, while co‐administration with an IDO1 inhibitor synergistically enhances antitumor immunity by alleviating the immunosuppressive tumor microenvironment. The CTSE‐responsive AuHQ system demonstrated enhanced photothermal efficiency (42.4%), 4.2‐fold fluorescence increase, and rapid tumor visualization (1 h), enabling effective pancreatic cancer theranostics. To address the challenges of tumor immune escape and enhance long‐term therapeutic outcomes, Fan et al. developed the all‐in‐one aPD‐L1@GdCDs nanoplatform, which successfully enables simultaneous triple‐modal imaging (FLI/PAI/MRI) and triple‐modal (PTT/PDT/checkpoint blockade (ICB)) therapy (Figure 12f).^[^ ^68^ ^]^ Under NIR laser irradiation (650 nm, 0.5 W cm^−2^, 10 min), the aPD‐L1@GdCDs nanoplatform triggers PTT and PDT effects for direct tumor cell death and ICD, promoting dendritic cell maturation and T‐cell activation. The controlled release of aPD‐L1 through mild hyperthermia blocks PD‐L1 on tumor cells, effectively reversing immunosuppression. In mouse models, this system demonstrated comprehensive therapeutic effects, including tumor growth suppression, enhanced survival, increased CD8^+^ T cell infiltration, elevated pro‐inflammatory cytokine levels, and inhibited metastasis through memory T cell formation. Zhang et al. further refined this approach by creating a PDA‐GQD bionanoprobe that combines immune checkpoint blockade with real‐time immune response monitoring via FRET for leukemia treatment.^[^ ^301^ ^]^ These modern platforms embody a combination of immunotherapy and phototherapy to both destroy local tumors and generate systemic immune responses against metastases while establishing long‐term immune surveillance against cancer recurrence.

### 3.2 Neurodegenerative Diseases

Alzheimer's disease (AD) represents one of the most significant challenges in modern healthcare, characterized by progressive neurodegeneration that manifests as severe memory loss and cognitive decline.^[^ ^302^ ^]^ The pathogenesis of AD involves three main interconnected hypotheses. The amyloid cascade and Tau hypothesis focus on the formation of senile plaques resulting from amyloid‐β (Aβ) aggregates and the development of neurofibrillary tangles (NFTs) from hyperphosphorylated Tau proteins.^[^ ^303^, ^304^ ^]^ The metal ion hypothesis elucidates the role of transition metals, particularly Cu^2^⁺, in facilitating Aβ aggregation and ROS generation.^[^ ^305^ ^]^ The oxidative stress hypothesis establishes the link between ROS imbalance and enhanced Aβ production, leading to neuronal deterioration.^[^ ^306^ ^]^ These pathological mechanisms form a complex, self‐perpetuating cycle where metal ions catalyze Aβ aggregation, subsequently amplifying ROS production, suggesting the necessity for multi‐targeted therapeutic interventions.

Current diagnostic approaches for AD face significant limitations, particularly in early‐stage detection. While conventional approaches combine clinical assessment, cognitive testing, and neuroimaging techniques with cerebrospinal fluid (CSF) biomarker analysis, these methods typically identify AD only after substantial neurological damage has occurred.^[^ ^307^ ^]^ The therapeutic landscape is equally challenging, with only five FDA‐approved medications available, including tacrine, donepezil, rivastigmine, galantamine, and memantine.^[^ ^308^ ^]^ While these interventions can provide temporary relief of symptoms, they have significant side effects and fail to address the underlying disease mechanisms or prevent disease progression. Additionally, a critical barrier in advancing AD treatment lies in the blood–brain barrier (BBB), which prevents ≈98% of potential therapeutic agents from reaching their intended targets in the brain.^[^ ^309^ ^]^ This physiological obstacle, combined with the limitations of current diagnostic tools, emphasizes the urgent need for innovative approaches in both detection and treatment strategies. The emergence of photo‐triggered nanotheranostic platforms offers promising solutions to these challenges through multiple mechanisms: 1) enhanced detection sensitivity for AD‐specific biomarkers via advanced imaging modalities such as NIR FLI and PAI, 2) improved BBB penetration through surface‐modified nanocarriers and photo‐triggered disruption techniques, and 3) controlled therapeutic delivery via photo‐responsive drug release systems. These platforms typically incorporate multifunctional nanostructures that combine imaging agents (e.g., fluorescent probes, PA contrast agents) with therapeutic payloads (e.g., Aβ aggregation inhibitors, antioxidants, metal chelators), activated by specific wavelengths of light to achieve spatiotemporal control over both diagnostic and therapeutic functions. The following sections will explore the applications of light‐triggered nanomedicine in AD, focusing on biosensing, bioimaging, and theranostics applications.

#### 3.2.1 Biosensing

The primary challenge in AD diagnosis lies in reliably detecting disease‐specific biomarkers, which are present at extremely low concentrations during early disease stages and often show biological overlap between AD patients, healthy individuals, and those with other neurological conditions. Current nanodiagnostic platforms target multiple AD‐specific biomarkers, including amyloid‐related markers (e.g., Aβ40, Aβ42, Aβ oligomers (AβO)),^[^ ^310^, ^311^ ^]^ various Tau proteins (e.g., total‐Tau (t‐Tau) and phosphorylated‐Tau (p‐Tau)),^[^ ^312^, ^313^ ^]^ small molecule biomarkers (e.g., acetylcholinesterase (AChE), hydrogen sulfide (H_2_S)),^[^ ^314^, ^315^ ^]^ and disease‐specific nucleic acids (e.g., miRNAs).^[^ ^316^ ^]^ These biomarkers can be detected in CSF and blood, with recent trends favoring blood‐based detection methods due to their less invasive nature.^[^ ^317^ ^]^ Additionally, the development of photo‐triggered nanomaterial‐based detection systems is now being developed to improve detection sensitivity and capabilities by enhancing signal transduction abilities, reducing background interference, improving specificity, and enabling multiplexed detection mechanisms, which can facilitate earlier and more accurate AD diagnosis.

##### PL‐Based Biosensing

**Figure 13:**
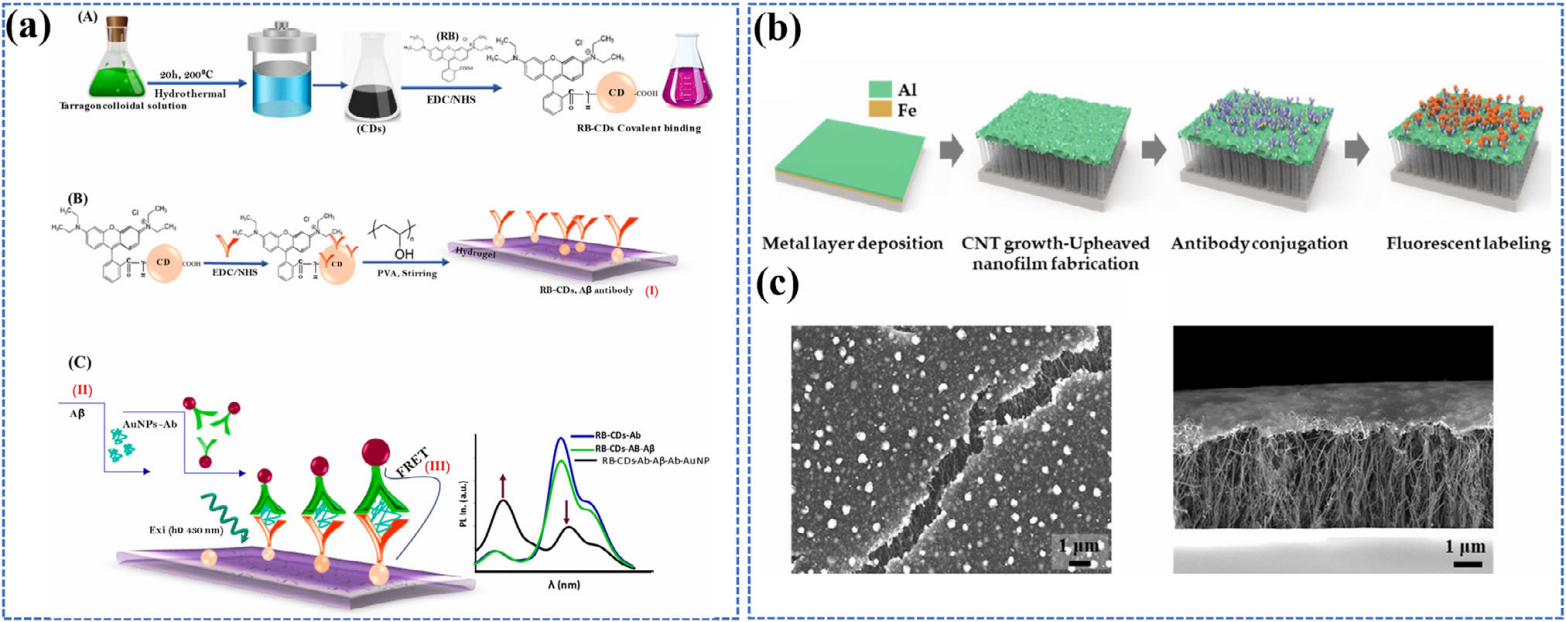
Photo‐triggered PL‐based biosensors for AD diagnosis. a) Schematic representation showing A) the fabrication of CDs, B) the CDs‐based hydrogel sensing platform, and C) the mechanism of Aβ peptide sensing. Reproduced with permission.^[^ ^318^ ^]^ Copyright 2021, Elsevier. b) Schematic depiction of the experimental process, c) Scanning electron microscope (SEM) image of the top and cross‐sectional view of CNT growth‐upheaved nanofilm with optimized growth time of 10 min. b,c) Reproduced with permission.^[^ ^321^ ^]^ Copyright 2024, Elsevier.

Recent advances in PL‐based biosensing for AD biomarkers have demonstrated remarkable progress through diverse detection mechanisms, with particular emphasis on dual‐signal and ratiometric approaches that effectively address traditional biosensing challenges such as background interference and signal stability. A notable example is the ratiometric FRET‐based hydrogel biosensor developed by Hamd‐Ghadareh et al., which employs Rhodamine B (RB)‐conjugated carbon dots in Polyvinyl alcohol (PVA) matrix and AuNPs.^[^ ^318^ ^]^ This system achieved remarkable sensitivity for Aβ peptide detection, reaching a detection limit of 0.5 pm through dual‐emission monitoring at I_582_/I_675_ nm in both serum samples and neuroblastoma cells under 430 nm excitation (**Figure** **13**a). Similarly, Liu et al. investigated a ratiometric fluorescent sensor using dual‐emission lanthanide metal‐organic coordination polymer (DE‐LMOCP) for Aβ detection in blood.^[^ ^319^ ^]^ The system utilizes Cu^2+^‐mediated fluorescence quenching of Tb^3+^, where Aβ presence triggers Cu^2+^ binding and Tb^3+^ fluorescence restoration while maintaining stable luminol emission. This ratiometric approach achieved a LOD of 20 pm for Aβ1‐40 in human plasma with high specificity. Dong et al. developed a dual‐ligand lanthanide‐based MOF (Eu‐MOF/BDC‐TPY) that demonstrated exceptional sensitivity for Zn^2+^ detection, achieving a remarkable LOD of 0.08 nm with a rapid response time of 5 s and impressive long‐term stability of 6 months.^[^ ^320^ ^]^

Most recently, novel devices have also emerged to enhance detection capabilities. Jang et al. established a large‐area fluorescent biosensor utilizing a CNT growth‐upheaved nanofilm for ultra‐sensitive detection of AD biomarkers in CSF (Figure 13b).^[^ ^321^ ^]^ The detection mechanism was achieved by optimizing the surface roughness and plasma‐treated nanofilm fabrication, where the CNT length after 10 min of growth was 7 µm, creating an ideal sensing platform for biomarker immobilization (Figure 13c). This study achieved sensitive detection of key AD biomarkers (including β‐amyloid, Tau, and APP) in both artificial and monkey CSF with detection limits as low as 0.023‐0.098 fm, which was much lower than traditional methods while requiring minimal sample volume. Xiao et al. reported an AuNPs layer‐modified paper‐based fluorescent immunosensor utilizing the internal filtering effect for the detection of phosphorylated Tau‐441 in human serum.^[^ ^322^ ^]^ The immunosensor adopted a sandwich sensing mechanism, where Tau‐441 was trapped between surface‐bound Ab1 and Au@CeO_2_‐conjugated Ab_2_, and detection was achieved by Au@CeO_2_‐catalyzed conversion of pNPP to PNP, followed by quenching of NGQDs fluorescence in proportion to Tau‐441 concentration under 365 nm UV irradiation. The system exhibits excellent sensitivity in human serum samples with a detection limit of 0.20 pg mL^−1^ and a detection range of 0.0005–10 ng mL^−1^.

##### SERS‐Based Biosensing

The development of SERS‐based biosensing for AD research has demonstrated remarkable progress, characterized by three significant trends. First, advanced material engineering has emerged as a crucial aspect, with researchers developing increasingly complex substrate designs.^[^ ^323^ ^]^ Wu et al. developed a graphene‐Au nanopyramid (GAuNP) hybrid SERS platform for ultrasensitive, label‐free detection of AD biomarkers including Tau, P‐Tau proteins, and Aβ42 peptides, achieving remarkable LODs of 10^−15^ m for Tau/P‐Tau proteins and 10^−14^ m for Aβ42 peptides.^[^ ^312^ ^]^ Similarly, Eremina et al. developed two SERS platforms, silver nanoparticle/chitosan (AgNPs/CS) and laser‐induced deposited AgNPs (AgNP/LID), for label‐free detection of Aβ aggregates, achieving nanomolar sensitivity at 532 nm laser and a LOD of 15 pm at 633 nm laser, while successfully distinguishing aggregates from monomers.^[^ ^324^ ^]^ Yang et al. further reported a sensitive SERS‐based immunosensor utilizing half antibody fragments on head‐flocked gold nanopillar substrates and SERS‐nanotags for blood‐based detection of Tau protein, achieving femtomolar‐level sensitivity (3.21 fm) under 785 nm laser excitation and successfully distinguishing AD patients from healthy controls in clinical plasma samples.^[^ ^325^ ^]^

**Figure 14:**
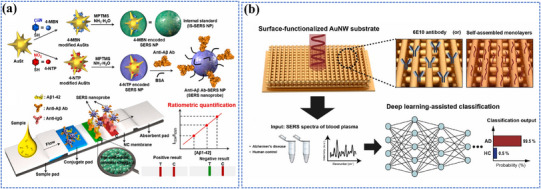
Photo‐triggered SERS‐based biosensors for AD diagnosis. a) The self‐calibrating SERS‐LFIA biosensing platform for quantitative analysis of Aβ1‐42 of Alzheimer's disease. Reproduced with permission.^[^ ^326^ ^]^ Copyright 2023, Elsevier. b) Deep learning‐assisted AuNW SERS platform for AD diagnosis. Reproduced with permission.^[^ ^330^ ^]^ Copyright 2024, Elsevier.

The second major trend focuses on practical clinical applications through integrated diagnostic platforms. Liu et al. presented a self‐calibrating SERS‐lateral flow immunoassay (SERS‐LFIA) biosensor with internal standard (IS)‐SERS nanoparticles for quantitative detection of Aβ1‐42 biomarker in biofluids.^[^ ^326^ ^]^ As shown in **Figure** **14**a, the system incorporates two types of SERS nanoparticles, where IS‐SERS NPs are encoded with 4‐MBN and detection SERS nanoprobes are encoded with 4‐NTP. IS‐SERS NPs were pre‐embedded into the T line of the test strip as a self‐calibration unit to correct signal fluctuations, while the detection SERS probes were deposited in the conjugate pad for target detection through sandwich immunoassay. The biosensor achieved remarkable sensitivity with a LOD of 12.1 pm in serum samples across a dynamic range of 0.1–50 nm, demonstrating excellent anti‐interference capability and reproducibility. Yuan et al. established another paper‐based microfluidic SERS immunoassay (NanoPAD) combining in‐situ grown AgNPs and AuNPs‐DTNB conjugates on nanocellulose paper, achieving 150 fg mL^−1^ detection limit for GFAP biomarker with high selectivity in artificial serum.^[^ ^327^ ^]^ Sun et al. developed a polystyrene/Au nanoparticles (PS/Au) composite microfluidic SERS platform with plasmon‐coupled microcavity for simultaneous detection of AD biomarkers Aβ_42_ and p‐Tau181 protein in human blood samples, achieving detection limits of 100 fg mL^−1^ via synergistic coupling between optical confinement of PS microcavity and LSPR effect of AuNPs.^[^ ^328^ ^]^ Most recently, Muhammad et al. created an aptamer‐functionalized SERS biosensing platform integrated with a glass microwell chip for multiplexed detection of blood‐based AD biomarkers, including neurogranin, angiopoietin‐2, PRDX3, L‐LDH, and τ‐441.^[^ ^329^ ^]^ This platform achieved atto‐molar sensitivity using CE‐SELEX‐generated aptamers and distinct Raman reporter‐labeled nanoprobes and was validated in mouse models and human clinical samples.

Lastly, an important trend is the integration of AI and machine learning with SERS technology. For instance, Kim et al. developed a deep learning‐assisted SERS platform using functionalized gold nanowire arrays (Figure 14b).^[^ ^330^ ^]^ The platform integrated the immobilization of 6E10 antibodies for the detection of Aβ_42_ with self‐assembled monolayers to facilitate interactions with metabolites. Advanced deep learning algorithms were used to analyze the SERS spectra, achieving classification accuracies of up to 99.5% for metabolite‐based analyses and 96–100% for Aβ_42_ oligomerization detection. Additionally, the system offers explainable AI insights to aid in biomarker identification. In the same way, Wang et al. developed another graphene‐enhanced Raman spectroscopy platform coupled with interpretable machine learning for AD biomarker screening, achieving 98% classification accuracy while identifying both known (Aβ, Tau) and novel biomarkers through SVM analysis of enhanced spectral signals.^[^ ^331^ ^]^

##### PEC Biosensing

**Figure 15:**
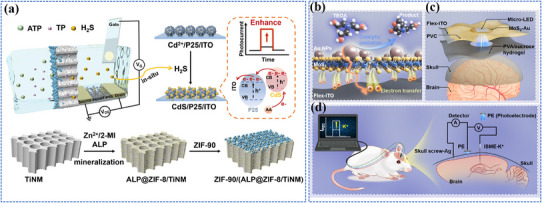
Photo‐triggered SERS‐based biosensors for AD diagnosis. a) Schematic illustrations of sensing mechanism and schedules for preparation of biomimetic nanochannels. Reproduced with permission.^[^ ^315^ ^]^ Copyright 2024, Wiley‐VCH. b) Diagram of the photo‐generated electron–hole separation and transfer process between MoS_2_–Au and Flex‐ITO. c) PVA/Sucrose hydrogel patches integrated with Flex‐ITO/MoS_2_–Au and micro‐LED, fixed onto the rat skull surface. d) Schematic of the PEC sensing system based on ISME potential regulation for real‐time monitoring of K+ concentration in the brain of awake animals. b–d) Reproduced with permission.^[^ ^337^ ^]^ Copyright 2024, American Chemical Society.

PEC‐based biosensing is gaining traction in AD research due to its high sensitivity and low background noise. Traditional Type II heterojunctions, exemplified by TiO₂/Au‐C₃N₄ systems developed by Li et al., demonstrated efficient charge separation enhanced by plasmonic effects of AuNPs.^[^ ^332^ ^]^ The biosensor achieved an impressive LOD of 0.33 fg mL^−1^ with a wide linear range (10⁻^15^ to 10⁻^11^ g mL^−1^) for Aβ40 detection and demonstrated successful validation in clinical CSF and plasma samples. More advanced designs have emerged with the development of Schottky heterojunctions. Wang et al. developed a self‐corrected split‐type PEC sensing platform with dual‐modal detection for ultrasensitive monitoring of Aβ1‐42.^[^ ^333^ ^]^ The system combines Ti_3_C_2_@Bi_2_WO_6_ Schottky heterojunction photoelectrodes with functionalized CaCO_3_@CuO_2_ nanocomposites, where glucose oxidation triggers cascade reactions leading to both colorimetric (TMB oxidation) and PEC signal changes. The dual‐modal platform achieved a LOD of 0.06 pg mL^−1^ while offering built‐in self‐correction capability. Recently, MOF‐based heterojunctions have attracted much attention due to their structural versatility and tunability, enhanced surface area and loading capacity, controlled mass transport, and improved charge separation efficiency. Lin et al. established a TiO_2_‐in‐MIL‐101(Cr)@CDs@AgNPs PEC sensing platform by confining TiO_2_ growth within MOF pores and incorporating dual signal enhancers (CDs and AgNPs) for ultrasensitive detection of amyloid‐β oligomers (AβO).^[^ ^334^ ^]^ This design achieved remarkable sensitivity (LOD 4.36 fm) with a 19‐fold enhancement in photocurrent density in human serum samples. Similarly, Zhang et al. created a magnetic PEC immunosensor array based on Fe_2_O_3_@FeS_2_@CdS MOF derivatives that enabled simultaneous detection of multiple AD biomarkers (Aβ and Tau) with detection limits of 2.1 and 7.9 pg mL^−1^, respectively, showcasing the versatility of MOF‐based platforms in multiplexed detection.^[^ ^335^ ^]^ Meanwhile, some study focuses on addressing practical challenges in the biological detection of AD. Qin et al.’s S‐scheme all‐polymer heterojunction utilizing NIR‐responsive PYIT‐C3N2 tackled the limitation of light penetration in nontransparent samples, demonstrating the ability to detect AChE in human blood with a LOD of 9.3 mU mL^−1^.^[^ ^314^ ^]^ Zheng et al. investigated a MOF‐guarded nanochannel design to prevent matrix interference through controlled molecular access.^[^ ^315^ ^]^ As shown in **Figure** **15**a, the contamination‐free organic PEC transistor (OPECT) system employs a TiO_2_ nanochannel membrane (TiNM) with ZIF‐90 MOF entrance guard, in which ATP is strongly chelated to the Zn(II) nodes in ZIF‐90, triggering the release of sodium thiophosphate (TP) substrate through the nanochannels. The confined alkaline phosphatase within the nanochannels could catalyze the production of H_2_S, which then reacted with Cd^2+^ to form CdS on the P25 gate electrode, thereby creating a type II heterojunction for enhanced photoelectric conversion. The nanochannel membrane effectively isolated the biological matrix from the detection cell to prevent interference and contamination. The biosensor achieved rapid ATP detection in undiluted serum within 15 min with an LOD of 0.32 nm, demonstrating its potential in real‐time clinical diagnosis.

Currently, another important trend in PEC biosensors for AD diagnosis is to achieve in vivo monitoring capabilities. Hao et al. developed a PEC microelectrode (Ti/TiO_2_@HSP) for real‐time monitoring of H_2_S in living brain tissue.^[^ ^336^ ^]^ The microelectrode was constructed by TiO_2_ nanotubes, which were then functionalized with the HSP probe, enabling specific recognition and photocurrent response to H_2_S under 560 nm excitation. The microelectrode demonstrated high sensitivity and selectivity for H_2_S and can effectively monitor dynamic changes in H_2_S levels across different regions of the mouse brain, revealing higher concentrations in the hippocampus (11.30 µm) compared to the striatum (3.55 µm) and cortex (2.20 µm). Wang et al. developed an excitation‐detection separated PEC platform for real‐time monitoring of K^+^ ions in awake rat brains.^[^ ^337^ ^]^ In Figure 15b, a flexible ITO electrode modified with MoS_2_ nanosheets and AuNPs was used as a photoactive electrode, and a potassium ion selective microelectrode (ISME‐K+) was used as a reference electrode. For in vivo analysis, ISME‐K+ was implanted in the brain while the photoelectrode with micro‐LED was left on the skull surface and assisted by a PVA/sucrose hydrogel patch (Figure 15c,d). By monitoring the photocurrent signal of the photoactive electrode, this platform successfully detected PM 2.5‐induced changes in extracellular K+ levels across different brain regions.

#### 3.2.2 Bioimaging

The field of photo‐triggered nanomaterials for AD bioimaging has shown remarkable progress, particularly in developing more precise and minimally invasive diagnostic tools. Current photo‐trigged bioimaging techniques for AD, including NIR‐FLI, SERS imaging, and PAI, have significantly improved our ability to visualize AD pathogenesis and monitor treatment responses.

##### PL Imaging

**Figure 16:**
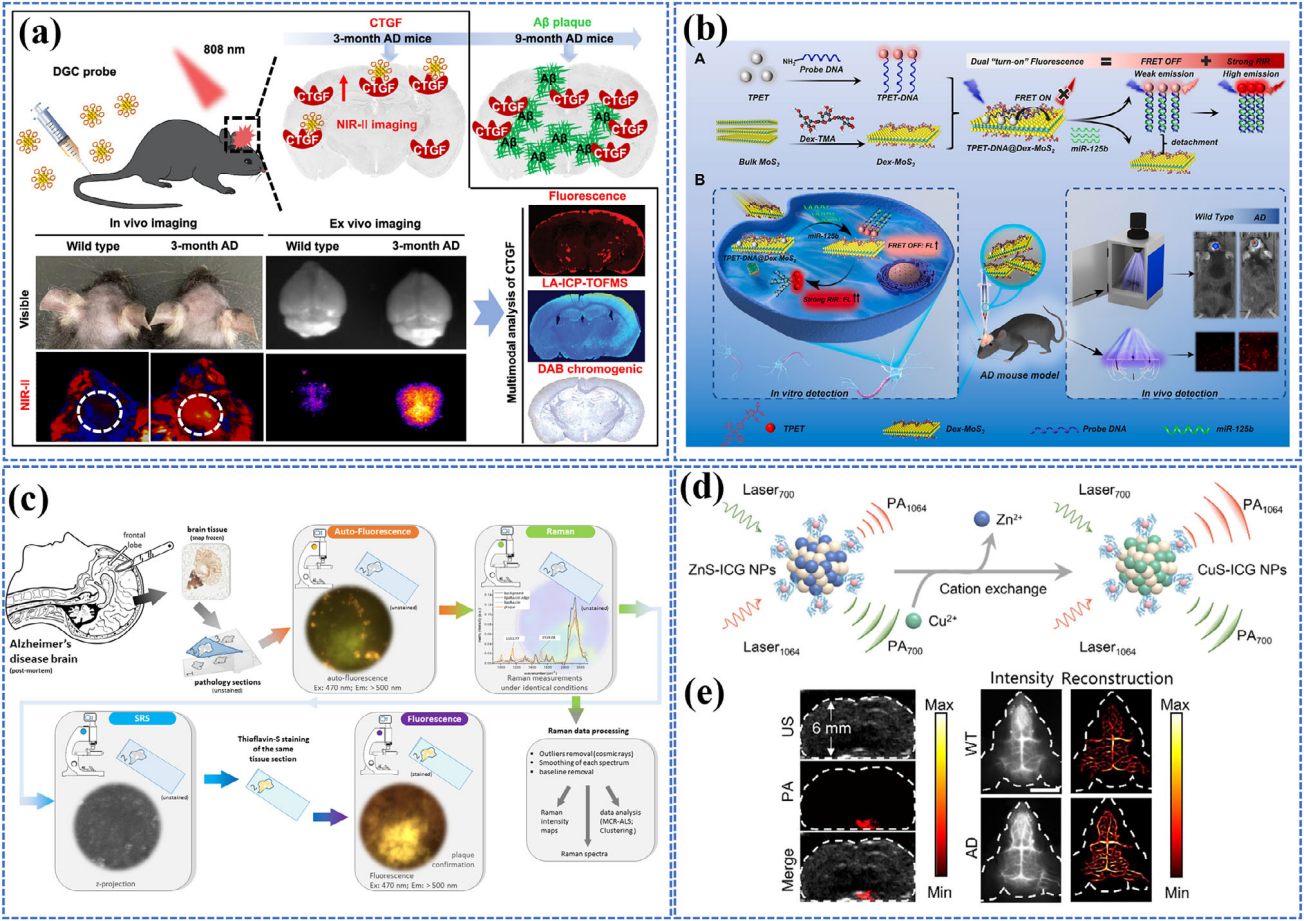
Photo‐triggered bioimaging systems for AD diagnosis. a) Schematic of the DGC probe identifying early AD by NIR‐II imaging and multimodal analysis. Reproduced under terms of the CC‐BY license.^[^ ^338^ ^]^ Copyright 2024, Lu et al., published by Springer Nature. b) Schematic diagram of the fabrication and sensing mechanism of TPET‐DNA@Dex‐MoS_2_ nanoprobes for in vitro and in vivo real‐time monitoring of miR‐125b overexpression in neurons and brain tissues of AD mouse model. Reproduced with permission.^[^ ^91^ ^]^ Copyright 2023, Elsevier. c) Experimental workflow. Reproduced under terms of the CC‐BY license.^[^ ^348^ ^]^ Copyright 2021 Lochocki et al., published by Springer Nature. d) Schematic representation of the activation of PAI through the cation exchange reaction between ZnS‐ICG NPs and copper ions in vitro. e) The penetration depth of PAI in the brain tissue of CO‐AD mice (left, scale bars = 2 mm) and NIR‐II FLI of cerebral vasculature in WT and CO‐AD mice (right, scale bar = 1 cm). d,e) Reproduced under terms of the CC‐BY license.^[^ ^350^ ^]^ Copyright 2024 Liao et al., published by Elsevier.

The development of early biomarker detection in AD imaging has evolved beyond traditional Aβ plaque visualization. Current research focuses on upstream regulators, protein oligomers, and oxidative stress markers, offering new avenues for early diagnosis and understanding of AD pathology. Lu et al. developed a connective tissue growth factor (CTGF)‐targeting AuNCs (DGC), which enabled detection before traditional Aβ plaque formation. CTGF is an upstream regulator of Aβ plaque formation.^[^ ^338^ ^]^ Their peptide‐coated AuNCs, composed of 26 gold atoms and eight cyclic peptide ligands, achieved remarkable CTGF binding affinity (KD ≈ 21.9 nm), which was more than 1000‐fold higher than that of the free peptide. Under 808 nm irradiation (100 mW cm^−2^), the multimodal detection capabilities of the DGC probe, including NIR‐II FLI, peroxidase‐like catalyzed chromogenic imaging, and ICP‐MS quantification, enabled the detection of elevated CTGF levels in APP/PS1 transgenic mice before Aβ plaque formation, effectively occurring before the 3‐month mark (**Figure** **16**a). Similarly, some fluorescent probes targeting Aβ oligomers have also been developed, demonstrating the emphasis on detecting preliminary stages of protein aggregation for early AD diagnosis.^[^ ^310^, ^339^ ^]^ Recent evidence has also shown that excessive ROS can promote Aβ production and aggregation, creating a vicious cycle in disease progression.^[^ ^340^, ^341^ ^]^ Zhang et al. developed a mitochondria‐targeting NIR fluorescent probe (HCy‐1) with dual fluorescence/afterglow imaging capabilities for real‐time visualization of hypochloric acid (HClO) in living cells and AD mouse brains.^[^ ^342^ ^]^ The probe utilized a hemocyanin‐based structure, enabling efficient penetration of the BBB and precise mitochondrial targeting. Upon the irradiation of 660 nm laser (1.0 W cm^−2^, 1 min) and rapid reaction with HClO, the dual signals of a fluorescence response with a 38‐fold enhancement at 715 nm and an afterglow emission can be generated within 10 min. This probe demonstrated high sensitivity with an LOD of 34.5 nm and achieved real‐time tracking of both exogenous and endogenous HClO in living PC‐12 nerve cells, RAW 264.7 macrophages, zebrafish models, and AD mouse brains. Chen et al. developed “turn‐off” fluorescent probes (NBD‐Y and NBD‐I) to detect peroxynitrite (ONOO^−^) via oxidative C‐N bond cleavage and intramolecular charge transfer mechanisms.^[^ ^343^ ^]^ The probes demonstrated rapid response (within 3 s) with high sensitivity (LOD: NBD‐Y 11 nm, NBD‐I 45 nm) and significant fluorescence changes (78‐fold decrease) under 488 nm excitation. Furthermore, their ability to effectively penetrate the BBB enabled successful visualization of ONOO^−^ in both cellular models (SH‐SY5Y cells) and AD mouse brains.

Another emerging direction involves enhanced targeting and signal amplification strategies. Current developments have explored the AIE‐based approach to overcome aggregation‐caused quenching.^[^ ^344^ ^]^ Zhang et al. developed a dual “turn‐on” fluorescence biosensor (TPET‐DNA@Dex‐MoS_2_) for detecting early AD biomarker miR‐125b, based on AIEgen‐labeled oligonucleotide probes immobilized on cationic dextran‐modified MoS_2_ (Figure 16b).^[^ ^91^ ^]^ This biosensor utilized AIEgen‐labeled oligonucleotide probes immobilized on cationic dextran‐modified MoS₂, offering a dual‐enhancement mechanism. Upon binding miR‐125b, the probe underwent target‐induced hybridization, causing the detachment of TPET‐DNA from Dex‐MoS₂. This triggered both FRET‐based signal recovery and AIEgen emission enhancement through restricted intramolecular rotation. Under 480 nm excitation, the biosensor achieved rapid detection (≤1 h) with high sensitivity (LOD: 20.81 pm) and successfully visualized the spatial association between miR‐125b and phosphorylated Tau protein in cellular and animal models of AD. Later, Xu et al. developed a novel NIR AIEgen, QMT‐CBT, designed for enhanced imaging of AD through dual aggregation.^[^ ^345^ ^]^ Upon activation by caspase1, a biomarker associated with AD neuroinflammation, QMT‐CBT undergoes a CBT‐Cys click reaction to form cyclic dimers (the first aggregation) and assembles into nanoparticles (the second aggregation), turning the AIE signal “on” for improved imaging sensitivity. This dual‐aggregation strategy achieved significantly enhanced fluorescence intensity (1.9‐fold in vitro, 1.7‐fold in cells, and 1.4‐fold in AD mouse models) compared to single‐aggregation controls.

##### SERS Imaging

The development of SERS imaging in AD research highlights the growing potential of nanotechnology‐based multimodal platforms for enhanced sensitivity, specificity, and mechanistic understanding of disease pathology. A major insight from recent advancements is the ability of SERS to provide real‐time, high‐resolution monitoring of Aβ aggregation and plaque formation. Zhou et al. developed a label‐free ratiometric SERS platform that employed in‐situ generated morphology‐distinct AuNPs as substrates, with Aβ serving as templates.^[^ ^346^ ^]^ This system enabled precise detection of protein conformational changes by analyzing spectral signatures of α‐helix (at 1268 cm^−1^) and β‐sheet (at 1244 cm^−1^) structures, with LODs of 70 pm for monomers and 3.0 pm for fibrils when monitoring Aβ aggregation in neurons and brain tissues in real time. It also revealed the differential effects of metal ions, such as Cu^2^⁺ and Zn^2^⁺ promoting fibrillation, while Fe^3^⁺ and Zn^2^⁺ at higher concentrations inhibited it, providing novel insights into Aβ aggregation dynamics. In another study, Xia et al. demonstrated the versatility of combining SERS imaging with FLI in a dual‐modal platform using Rose Bengal‐conjugated AuNPs (RB‐AuNPs).^[^ ^347^ ^]^ Under 633 nm laser exposure, the nanoprobe allowed for the quantification of Aβ42 peptides and superior tracking of amyloid progression in brain tissue with a high signal‐to‐noise ratio, offering a more sensitive alternative to traditional staining techniques. Extending beyond SERS alone, Lochocki et al. developed a comprehensive multimodal label‐free imaging platform combining high‐resolution FLI, pre‐resonance Raman mapping, and stimulated Raman scattering (SRS) for characterizing amyloid deposits in human AD brain tissue (Figure 16c).^[^ ^348^ ^]^ The system utilized auto‐fluorescence for initial plaque identification, followed by spectroscopic analysis using Raman mapping at 532 nm excitation for chemical specificity and SRS for protein conformational changes detection, with subsequent thioflavin‐S staining providing direct validation of plaque locations. The integrated approach revealed novel findings, including the identification of carotenoids as markers for cored amyloid plaques, observation of protein peak shifts towards β‐sheet structure specifically in cored plaques through SRS imaging, and distinct spectroscopic signatures between cored and fibrillar plaques, demonstrating the potential of label‐free multimodal imaging in understanding neuroinflammatory responses and plaque pathology in AD.

##### PAI

PAI is an emerging technique that combines optical and ultrasound imaging to provide high‐resolution images of brain tissues. Recent advancements in the use of photo‐triggered nanomaterials, particularly those optimized for the NIR‐II region, have demonstrated significant potential for improving imaging depth and contrast, which is essential for AD diagnosis and monitoring. For instance, Han et al. developed ultrathin ZnSe nanoplatelets modified with angiopep‐2 peptide for targeting BBB and activatable PAI to monitor brain copper levels in AD.^[^ ^349^ ^]^ The developed nanoplatelets can effectively cross the BBB with the help of targeting peptides and perform in situ cation exchange with endogenous copper ions in the brain to form copper selenide (CuSe) nanocrystals that enhance PAI signals. Under 700 nm irradiation, the system demonstrated high sensitivity with a LOD of 80 nm. Building on this, Liao et al. developed another ZnS‐ICG nanoparticles (NPs) that utilize the meningeal lymphatic vessel (MLV) pathway to bypass the BBB, facilitating brain delivery without complex modifications.^[^ ^350^ ^]^ These ZnS‐ICG NPs undergo a cation exchange reaction with copper ions, transforming into CuS‐ICG NPs, which enhances NIR‐II absorption and facilitates high‐sensitivity, ratiometric PAI with a penetration depth up to 6 mm (Figure 16d). By monitoring the ratiometric signals at 700 and 1064 nm, the approach effectively eliminates background interference and achieves a LOD of 0.22 mm. Furthermore, the nanoprobes can efficiently cross the BBB via the meningeal lymphatic system and provide improved imaging performance, with tissue penetration depths of up to 6 mm and high spatial resolution of 10 µm (Figure 16e). These advancements in PAI demonstrate the transformative potential of nanotechnology‐driven NIR‐II imaging systems for non‐invasive AD diagnosis and monitoring, offering deeper tissue visualization, higher resolution, and exceptional sensitivity to key AD biomarkers.

#### 3.2.3 Multimodality Therapy

Unlike cancer phototherapy, AD treatment faces challenges due to the presence of the BBB and the diffuse nature of pathology throughout brain tissue. The therapeutic goals shift from direct tumor ablation to more subtle interventions, such as disrupting protein aggregates, providing neuroprotection, and modulating neuroinflammation. The complex neural environment of the brain demands extremely precise control over both thermal effects and ROS generation, as the tolerance for collateral damage is minimal compared to cancer therapy. In addition, the better oxygenation of brain tissue potentially favors PDT efficacy, though careful control is essential to prevent neuronal damage.

##### PDT

In AD treatment, PDT utilizes the interaction between light and photosensitizers to produce ROS, which can modulate Aβ aggregation and potentially restore cognitive functions.^[^ ^351^, ^352^ ^]^ PDT offers several advantages, including high selectivity through localized photosensitizers, minimal invasiveness, and controllable ROS generation, making it suitable for repeated treatment cycles in AD.

**Figure 17:**
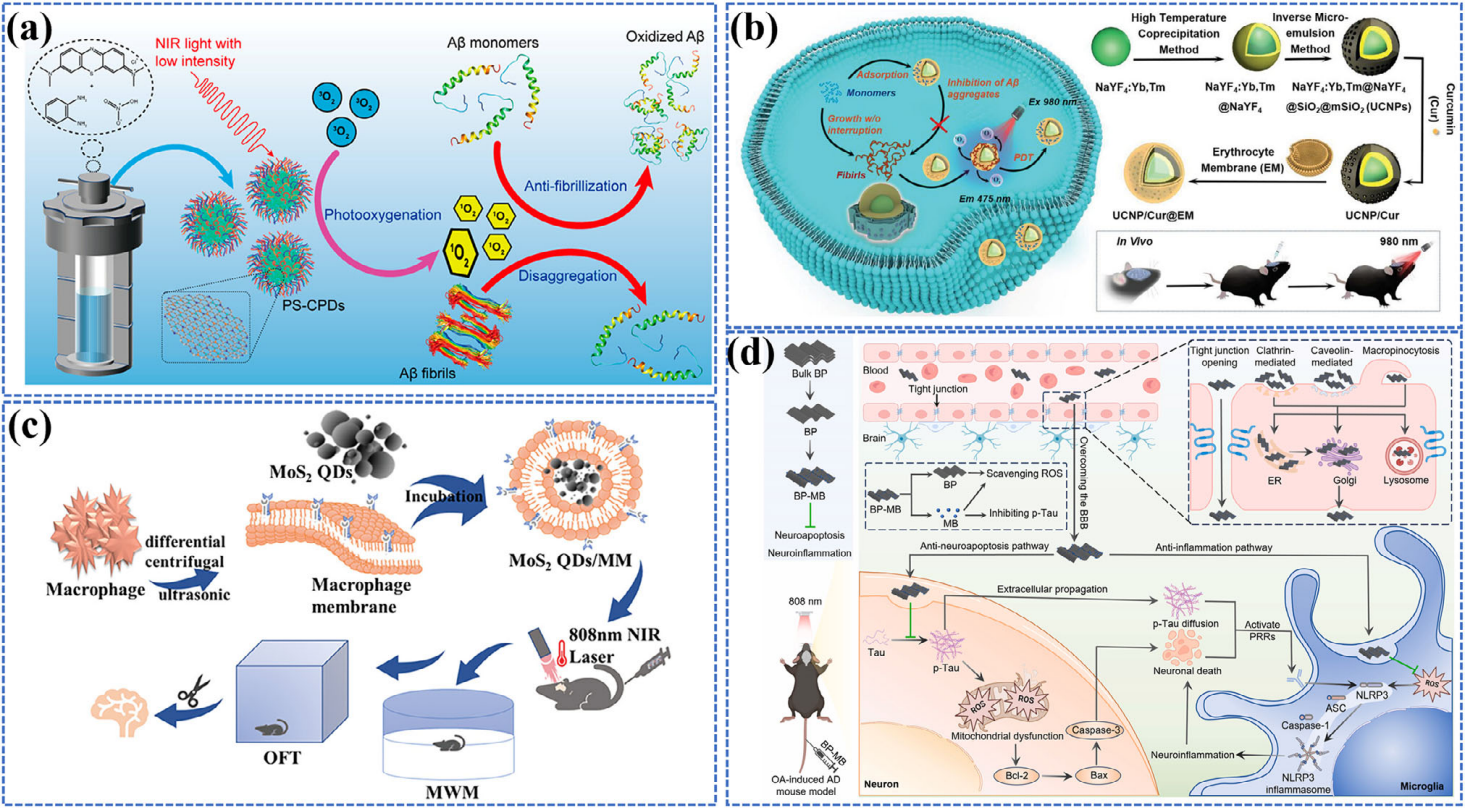
PDT and PTT for Alzheimer's disease. a) Methylene blue‐doped carbonized polymer dots: a potent photooxygenation scavenger targeting Alzheimer's β‐amyloid. Reproduced with permission.^[^ ^356^ ^]^ Copyright 2023, American Chemical Society. b) Schematic illustration of biomimetic upconversion nanobait‐based PDT for the inhibition of Aβ aggregates. Reproduced with permission.^[^ ^357^ ^]^ Copyright 2023, Wiley‐VCH. c) Schematic diagram of the synthesis of MoS_2_ QDs/MM System and its combined photo‐chemotherapy against AD. Reproduced with permission.^[^ ^363^ ^]^ Copyright 2023, Wiley‐VCH. d) Schematic representation of BP‐MB treatment in OA‐induced mouse models of AD. Reproduced with permission.^[^ ^365^ ^]^ Copyright 2024, Elsevier.

Wang et al. developed porphyrinic MOF (PCN‐224) nanoparticles for NIR‐induced suppression of Aβ aggregation, which significantly reduced Aβ‐induced cytotoxicity and achieved a cell survival rate of 90% after photooxygenation treatment.^[^ ^353^ ^]^ Lin et al. developed photosensitizer‐doped carbonized polymer dots (PS‐CPDs) with enhanced photooxygenation capability for inhibiting Aβ aggregation in AD (**Figure** **17**a).^[^ ^354^ ^]^ Under 650 nm NIR light irradiation, the MB‐CPDs demonstrated superior therapeutic efficacy compared to free MB and conventional carbon dots, achieving 83% cell viability at just 2 µg mL^−1^ concentration and extending AD nematode lifespan by 4 days, while maintaining moderate BBB penetration ability. After that, Zhang et al. reported a NIR‐II‐responsive hydrogen‐bonded organic framework (DSM@n‐HOF‐6@KD8) incorporating pyridinium hemicyanine dye DSM for targeted PDT of AD.^[^ ^355^ ^]^ The system utilized two‐photon NIR‐II‐excited FRET between DSM and porphyrin‐based HOF to generate singlet oxygen, while KLVFFAED peptide (KD8) modification enables BBB crossing and Aβ targeting. Upon 1040 nm NIR‐II irradiation (700 mW, 20 min, every 3 days over a 15‐day period), DSM@n‐HOF‐6@KD8 significantly reduced the neurotoxicity of Aβ plaques and improved cognitive function in triple‐transgenic AD model mice. Similarly, Liu et al. developed a protein‐based system using human serum albumin‐stabilized AuNCs (AuNCs@HSA‐B), which exhibited functionality in Aβ inhibition via photooxygenation.^[^ ^356^ ^]^ Most recently, Wang et al. developed an erythrocyte membrane (EM)‐modified core‐shell upconversion nanoparticle with curcumin (Cur) loading (UCNP/Cur@EM) for PDT of AD (Figure 17b).^[^ ^357^ ^]^ Upon NIR light irradiation (980 nm, 0.5 W cm^−2^, 10 min), the UCNPs activate Cur to generate ROS, overcoming tissue penetration limitations of traditional PDT. At the same time, the EM coating ensures Aβ aggregates are trapped in the phospholipid bilayer, bringing them close to Cur. This proximity improves ROS utilization efficiency for degrading Aβ aggregates, overcoming the short half‐life of ROS. In the APP/PS1 transgenic mouse model, this biomimetic “nanobait” system reduced Aβ deposits and improved cognitive functions, offering a biocompatible, controllable, and effective strategy for treating amyloid‐related pathologies.

##### PTT‐Based Multi‐Target Platforms

PTT has emerged as a promising strategy in AD treatment utilizing localized hyperthermia to induce therapeutic effects such as protein denaturation, thermal ablation of Aβ aggregates, and enhanced BBB permeability.^[^ ^358^ ^]^ Recent advancements in PTT have focused on integrating complementary therapeutic mechanisms, such as metal ion chelation, oxidative stress modulation, and neuroinflammation targeting, to address the multifaceted pathology of AD. For example, Ye et al.^[^ ^359^ ^]^ and Li et al.^[^ ^360^ ^]^ developed NIR‐responsive PTT to promote Aβ depolymerization and reduce the toxic effects of Cu^2^⁺‐mediated aggregation. Wang et al. developed a developed a multifunctional nanocarrier (CICe@M‐K) that integrated curcumin, IR780, CeO_2_ nanoparticles, and targeting peptide K for simultaneous inhibition of β‐amyloid aggregation and ROS scavenging.^[^ ^361^ ^]^ This platform employs NIR‐triggered release of curcumin and peptide K to prevent Aβ aggregation, while CeO₂ nanoparticles synergistically eliminate excessive ROS in the brain microenvironment. Similarly, Song et al. developed CKLVFFAED peptide‐modified Prussian blue nanoparticles (PBK NPs) to target ROS elimination and enhance PTT efficacy.^[^ ^362^ ^]^ In another study, Xie et al. developed a black phosphorus‐methylene blue nanosheet system (BP‐MB), which demonstrated multiple therapeutic effects, including photothermal‐enhanced BBB penetration, ROS scavenging, and modulation of microglial polarization (Figure 17d).^[^ ^365^ ^]^ Under NIR irradiation (808 nm, 1.5 W cm^−2^), BP‐MB nanosheets facilitated BBB crossing via caveolae‐ and clathrin‐mediated pathways. Once in the brain parenchyma, they effectively reduced Tau hyperphosphorylation, mitigated mitochondrial dysfunction, and attenuated neuroapoptosis and neuroinflammation. Additionally, BP‐MB promoted the anti‐inflammatory M2 polarization of microglia, significantly improving cognitive function in an AD mouse model without causing major organ damage or adverse effects. Recently, biomimetic systems have also gained traction in PTT‐based AD treatment. Qi et al. developed a membrane‐encapsulated MoS_2_ QDs (MoS_2_ QDs/MM) for multi‐target treatment of AD (Figure 17c).^[^ ^363^ ^]^ The system combines inherent enzyme‐like activities and photothermal properties of MoS_2_ QDs with macrophage membrane coating for inflammation targeting and immune evasion, while utilizing NIR irradiation (808 nm, 2 W cm^−2^, 5 min) to enhance BBB penetration and thermal ablate Aβ aggregates. Liu et al. developed a red blood cell membrane‐encapsulated CQDs‐polydopamine nanocomposite (PDA‐CQDs/RBC) to combine metal chelation, enzyme‐mimicking activity, and PTT for effective Aβ reduction.^[^ ^364^ ^]^ Last, the combination of PDT and PTT has shown promising results in treating AD. Wang et al. developed a NIR‐responsive multifunctional nanoplatform (PCN‐222@ICG@RVG) that employs photothermal effects to maintain Aβ monomeric states while simultaneously generating photo‐oxygenation to reduce Aβ aggregation potential.^[^ ^366^ ^]^ The incorporation of RVG peptide enables targeted BBB penetration via nicotinic acetylcholine receptor recognition on brain endothelial cells. Using a human brain‐on‐a‐chip model and *ex vivo* studies, they demonstrated effective BBB crossing and significant reduction of Aβ plaques, validating the therapeutic potential of this integrated approach.

##### Phototheranostics

The developments in imaging‐guided multimodality therapy have shown great promise in addressing the complex pathology of AD. By integrating advanced nanocarrier systems with real‐time monitoring capabilities, these platforms offer enhanced precision in drug delivery and therapeutic response evaluation, addressing multiple AD pathologies simultaneously.^[^ ^367^ ^]^ Huang et al. developed a traceable central nervous system drug delivery nanoformulation (RVG‐NV‐NPs), which encapsulated bexarotene and AgAuSe QDs within neural stem cell membranes overexpressing Lamp2b‐RVG peptides.^[^ ^368^ ^]^ The brain‐targeted RVG peptide possesses the natural brain‐homing properties of NSC membranes, effectively penetrating the BBB and targeting neural cells. The encapsulated AgAuSe QDs enabled multiscale in vivo monitoring of delivery processes, from systemic circulation to single‐cell targeting, via NIR‐II FLI. Simultaneously, the released bexarotene upregulates ApoE expression to enhance Aβ clearance. Using just 0.5% of the conventional oral dose, this nanoformulation achieved superior therapeutic efficacy with ∼40% reduction in brain Aβ levels and significant cognitive improvement in AD mice, demonstrating a novel strategy for efficient central nervous system‐targeted drug delivery with real‐time imaging capability. Gu et al. developed a neuron‐targeting multifunctional nanocomposite integrating tannic acid (TA)‐based nanoparticles with manganese ions, IR780 dye, and TPL peptide (IR780‐Mn@TA‐TPL) for MRI/FLI‐guided AD therapy.^[^ ^369^ ^]^ The nanocomposite exploited the antioxidant properties of tannic acid for ROS scavenging and tau pathology inhibition, while TPL peptide enhanced BBB penetration and neuron‐specific targeting. MRI and NIR‐FLI were achieved through Mn^2+^ chelation and IR780 encapsulation, facilitating real‐time monitoring of drug delivery and therapeutic efficacy. As a result, this multifunctional nanocomposite could effectively alleviate mitochondrial damage by reducing oxidative stress, inhibit tau pathology by activating Akt/GSK‐3β signaling pathway, and prevent neuronal apoptosis by regulating pro‐apoptotic proteins, ultimately improving neuronal density and spatial memory in AD rat models.

**Figure 18:**
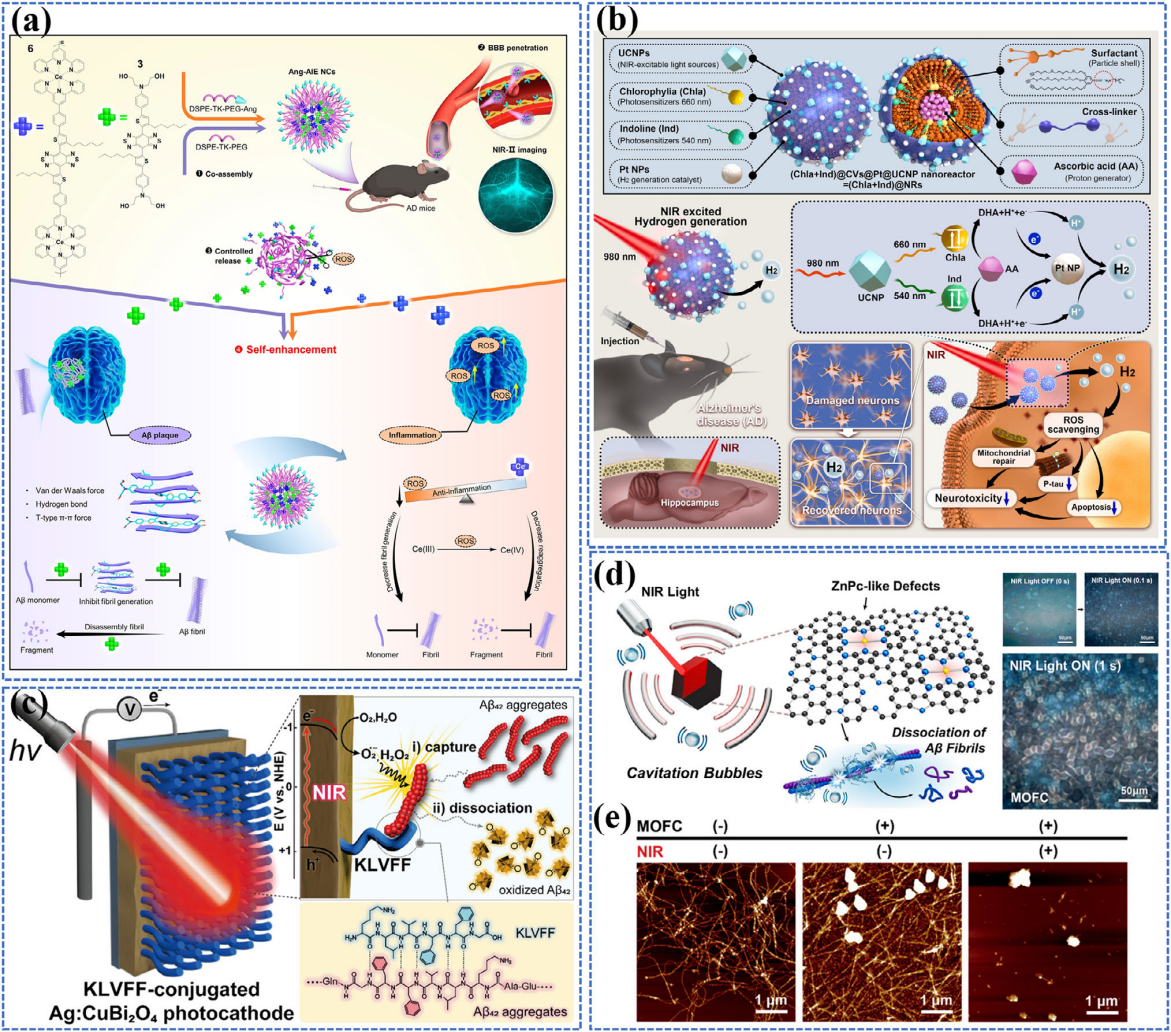
Phototheranostics and other phototherapies for AD. a) Schematic of NIR‐II brain‐target theranostics system for dual‐target therapy of AD via a four‐step route. Reproduced under terms of the CC‐BY license.^[^ ^370^ ^]^ Copyright 2024 Wang et al., published by Springer Nature. b) Schematic diagram illustrating the preparation of (Chla + Ind)@NR and NIR‐triggered in situ H_2_ release from (Chla + Ind)@NR to scavenge local excess ROS, alleviate mitochondria damage, and reduce tau hyperphosphorylation for AD therapy. Reproduced under terms of the CC BY‐NC‐ND license.^[^ ^372^ ^]^ Copyright 2024 Zhang et al., published by Elsevier. c) Schematic illustration of disaggregation of Aβ assemblies by the KLVFF‐Ag:CuBi_2_O_4_ photocathode. Reproduced with permission.^[^ ^373^ ^]^ Copyright 2020, American Chemical Society. d) Schematic illustration of the photoacoustic dissociation of Alzheimer's Aβ aggregate structure. e) AFM images of Aβ fibrils after treatment of MOFC nanoparticles (100 µg mL^−1^) and NIR light (λ = 808 nm, 1 W cm^−2^) for 2 h. d,e) Reproduced with permission.^[^ ^374^ ^]^ Copyright 2022, American Chemical Society.

Notably, some platforms can simultaneously target multiple AD pathologies. Wang et al. developed a brain‐targeting NIR‐II nanotheranostic system (Ang‐AIE NCs) incorporating two therapeutic AIEgens‐compound 3 for Aβ fibril degradation and compound 6 for ROS scavenging to achieve dual‐targeted therapy for AD (**Figure** **18**a).^[^ ^370^ ^]^ The system uses ROS‐responsive thioketal‐containing templates for co‐assembly and angiopep‐2 modification for enhanced BBB penetration, while the AIEgens enable NIR‐II imaging up to 1350 nm (excitation at 808 nm) for monitoring drug delivery and therapeutic response. Under inflammatory conditions, the nanocarriers could release compound 3 to inhibit Aβ fibril formation through multiple molecular interactions and compound 6 to scavenge ROS via Ce(III) active centers. The system also demonstrated an extended in vivo elimination half‐life of 4.8 h and significantly reduced Aβ plaques, alleviated inflammation, reversed neurotoxicity, and improved cognitive and behavioral outcomes in AD mouse models.

##### Other Phototherapies of AD

In addition to PTT and PDT, photocatalytic, photoelectric, and PA therapy have recently emerged as alternative treatments for AD. For photocatalytic therapy, Ge et al. developed a UCNP‐doped g‐C_3_N_4_ upconversion photocatalyst platform (UCNP@CoP@g‐C_3_N_4_) for AD treatment, which combines NIR‐responsive continuous hydrogen production, metal ion chelation, and PTT to simultaneously address oxidative stress and Aβ accumulation.^[^ ^371^ ^]^ Zhang et al. advanced the field further with their biomimetic nanoreactor approach.^[^ ^372^ ^]^ As illustrated in Figure 18b, their system employs biocompatible crosslinked vesicles as its foundational structure, incorporating two photosensitizers, chlorophyll a (Chla) and indoline dye (Ind), to enable broader spectrum light absorption and enhanced photocatalytic efficiency. UCNPs were used as light‐harvesting antennas, while platinum nanoparticles (Pt NPs) acted as photocatalysts. Under NIR irradiation (980 nm, 0.5 W cm^−2^, 20 min), this integrated system efficiently converted light energy into localized hydrogen gas, scavenging local ROS and mitigating tau hyperphosphorylation in AD models. This innovative approach not only restores mitochondrial function and neuronal health but also improves cognitive outcomes in AD mice, demonstrating the immense potential of nanotechnology‐enabled hydrogen therapy for AD.

Photoelectric therapy employs light‐driven electron‐hole pairs to generate reactive species or induce redox reactions for targeted therapeutic effects. Heo et al. developed a NIR‐active photocathodic platform based on silver‐doped copper bismuth oxide (Ag:CuBi₂O₄) for targeted dissociation of Aβ aggregates into nontoxic, soluble species. By utilizing the NIR absorption and narrow band gap (1.5–1.8 eV) of CuBi₂O₄, the platform achieved deeper tissue penetration with minimal photodamage (Figure 18c).^[^ ^373^ ^]^ To enhance selectivity, the photocathode surface was conjugated with the KLVFF peptide, which specifically binds Aβ aggregates. Under NIR light and cathodic bias, the KLVFF‐Ag:CuBi₂O₄ platform could generate O_2_^•−^, effectively breaking down β‐sheet‐rich Aβ fibril aggregates into small, non‐toxic, soluble species.

PA therapy utilizes light‐induced mechanical forces generated by cavitation bubbles to disrupt Aβ aggregates. Jang et al. reported MOF‐derived carbon (MOFC) nanoparticles that can generate intense cavitation bubbles within milliseconds upon NIR light absorption (808 nm, 1 W cm^−2^) (Figure 18d).^[^ ^374^ ^]^ The rapid formation and collapse of these bubbles could generate mechanical forces mediated by water molecules, targeting the negatively charged amino acid residues (Glu22 and Asp23) in the β‐strands of Aβ aggregates, thereby effectively destroying their β‐sheet structure and converting toxic fibrillar aggregates into non‐toxic globular debris. AFM imaging confirmed this result, showing the transition from micrometer‐long fibers to smaller fragments (Figure 18e).

### 3.3 Retinal Degeneration

Retinal degeneration encompasses a spectrum of progressive disorders characterized by the deterioration of photoreceptors and retinal pigment epithelium (RPE), ultimately leading to vision loss.^[^ ^375^ ^]^ Among these, age‐related macular degeneration (AMD) and retinitis pigmentosa (RP) represent two distinct pathological patterns. AMD primarily affects the macula, resulting in central vision loss, and manifests in two forms: dry AMD (dAMD characterized by drusen formation and RPE atrophy) and wet AMD (wAMD marked by choroidal neovascularization (CNV)).^[^ ^376^ ^]^ RP initially affects rod photoreceptors in the peripheral retina, leading to the loss of “tunnel vision”. Both of them can lead to severe visual impairment and eventually blindness.^[^ ^377^ ^]^ Current therapeutic strategies for vision restoration in AMD and RP follow distinct pathways based on their disease mechanisms. For AMD, treatments primarily focus on managing disease progression, with anti‐vascular endothelial growth factor (VEGF) injections being the standard care for wet AMD, while dry AMD relies mainly on nutritional supplements and preventive measures.^[^ ^378^, ^379^ ^]^ RP treatment approaches include gene therapy (notably Luxturna for specific genetic mutations), experimental stem cell therapies, and various neuroprotective strategies.^[^ ^380^ ^]^ However, both conditions still lack definitive cures, driving the development of more precise treatments.

Emerging photo‐triggered nanomaterial‐based therapeutic platforms offer significant promise for retinal degeneration by enabling localized, minimally invasive treatment with reduced systemic side effects. For wet AMD, PDT and photo‐responsive drug delivery systems are undergoing refinements via nanomaterial engineering and photosensitizer design to enhance drug stability, targeting, and activation efficiency. In RP, optogenetic approaches and photoelectric nanodevices are being explored to restore visual function by converting light into electrical signals to stimulate surviving retinal neurons. While these photo‐triggered nanotechnologies are still in experimental stages, they offer a versatile platform for addressing retinal degeneration.

#### 3.3.1 PDT

**Figure 19:**
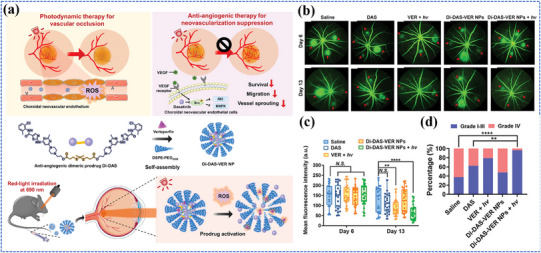
PDT for age‐related macular degeneration. a) Schematic illustration of the photoactivatable prodrug‐based nanosystem (Di‐DAS‐VER NPs) for anti‐angiogenic and photodynamic combination therapy of wAMD. b,c) Representative fundus fluorescein angiography images and the corresponding quantification results of mean fluorescence intensity on days 6 and 13. Red arrows denote the leaking CNV regions. *n* = 8 mice, 24–28 lesions. d) Clinical grading of the fluorescein leakage degree on day 13 according to (b,c). a–d) Reproduced under terms of the CC‐BY license.^[^ ^381^ ^]^ Copyright 2023 Xu et al., published by Wiley‐VCH.

AMD is a leading cause of vision loss in older adults, particularly in developed countries. Neovascular AMD (wAMD), characterized by the pathological growth of new blood vessels (CNV) beneath the retina, often results in significant vision distortion and scarring. PDT has been explored as a treatment option to selectively target these neovascular membranes, minimizing damage to the surrounding retina. Xu et al. developed a photoactivatable nanosystem (Di‐DAS‐VER NPs) combining PDT and anti‐angiogenic therapy for wAMD treatment through intravenous administration (**Figure** **19**a).^[^ ^381^ ^]^ The nanosystem was constructed by self‐assembling a ROS‐sensitive dasatinib (DAS) prodrug with photosensitizer verteporfin. Upon irradiation with 690 nm red light directed at the diseased eyes, the system generates ROS via verteporfin for PDT, while simultaneously triggering the controlled release of the anti‐angiogenic drug DAS, creating a synergistic therapeutic effect localized to the target site. As shown in Figure 19b,c, fundus fluorescein angiography (FFA) was used to evaluate CNV lesion vascular leakage before (day 6) and after (day 13) treatments. The saline control and Di‐DAS‐VER NPs without light irradiation groups maintained high vascular leakage (87.1% and 87.8% of day 6 levels). In contrast, Di‐DAS‐VER NPs with light irradiation (690 nm, 80 mW cm^−2^, 312 s) significantly reduced leakage to 38.6%, outperforming monotherapy with DAS (76.1%) or VER with light (55.9%). Further clinical grading revealed that 62.5% of lesions in the saline‐treated group were classified as severe leakage (grade IV), compared to only 3.6% in the Di‐DAS‐VER NPs plus light group, demonstrating the superior efficacy of the combination therapy (Figure 19d). After that, Xu et al. improved the photoactivatable nanosystem by transforming drug delivery into topical eye drops, which utilized the natural oxidative microenvironment of CNV to activate drugs without external light triggers, simplifying the treatment process.^[^ ^382^ ^]^

Despite the advancements in PDT‐based therapies, the introduction of anti‐VEGF therapy has transformed the clinical management of wAMD. Anti‐VEGF injections are now the gold standard, effectively targeting the underlying angiogenic mechanisms of the disease. Consequently, PDT has transitioned from a primary treatment to a specialized adjunctive role. PDT is now primarily utilized in specific scenarios, such as in the treatment of polypoidal choroidal vasculopathy, cases resistant to anti‐VEGF therapy, or in patients for whom frequent anti‐VEGF injections are contraindicated or impractical.^[^ ^383^, ^384^ ^]^

#### 3.3.2 Photoelectric Strategy

Artificial retinal technologies have emerged as a promising approach for vision restoration, particularly in cases where photoreceptors are completely lost. These technologies can be broadly categorized into two main approaches: electronic retinal prostheses and photo‐responsive materials. Traditional electronic prostheses, such as subretinal (PRIMA, Alpha AMS) and epiretinal (Argus II) implants, have demonstrated clinical success but face limitations in resolution and biocompatibility due to their rigid electronic components, reliance on external power sources, and complex signal processing requirements.^[^ ^385^ ^]^ In contrast, emerging approaches based on photo‐responsive materials offer significant advantages by mimicking the natural light‐sensing properties of photoreceptors without requiring external power or bulky electronics.^[^ ^386^ ^]^ These materials, such as organic semiconductors,^[^ ^387^ ^]^ Metal oxide (TiO_2_),^[^ ^388^ ^]^ 2D nanomaterials (MoS_2_),^[^ ^389^, ^390^, ^391^ ^]^ carbon‐based materials,^[^ ^392^ ^]^ and hybrid nanomaterials,^[^ ^32^, ^393^ ^]^ are capable of directly converting light into bioelectric signals while conforming seamlessly to the curved and delicate structure of the retina. Their nanoscale design improves biocompatibility, reduces inflammation, and enables higher resolution by interfacing more precisely with retinal neurons.

**Figure 20:**
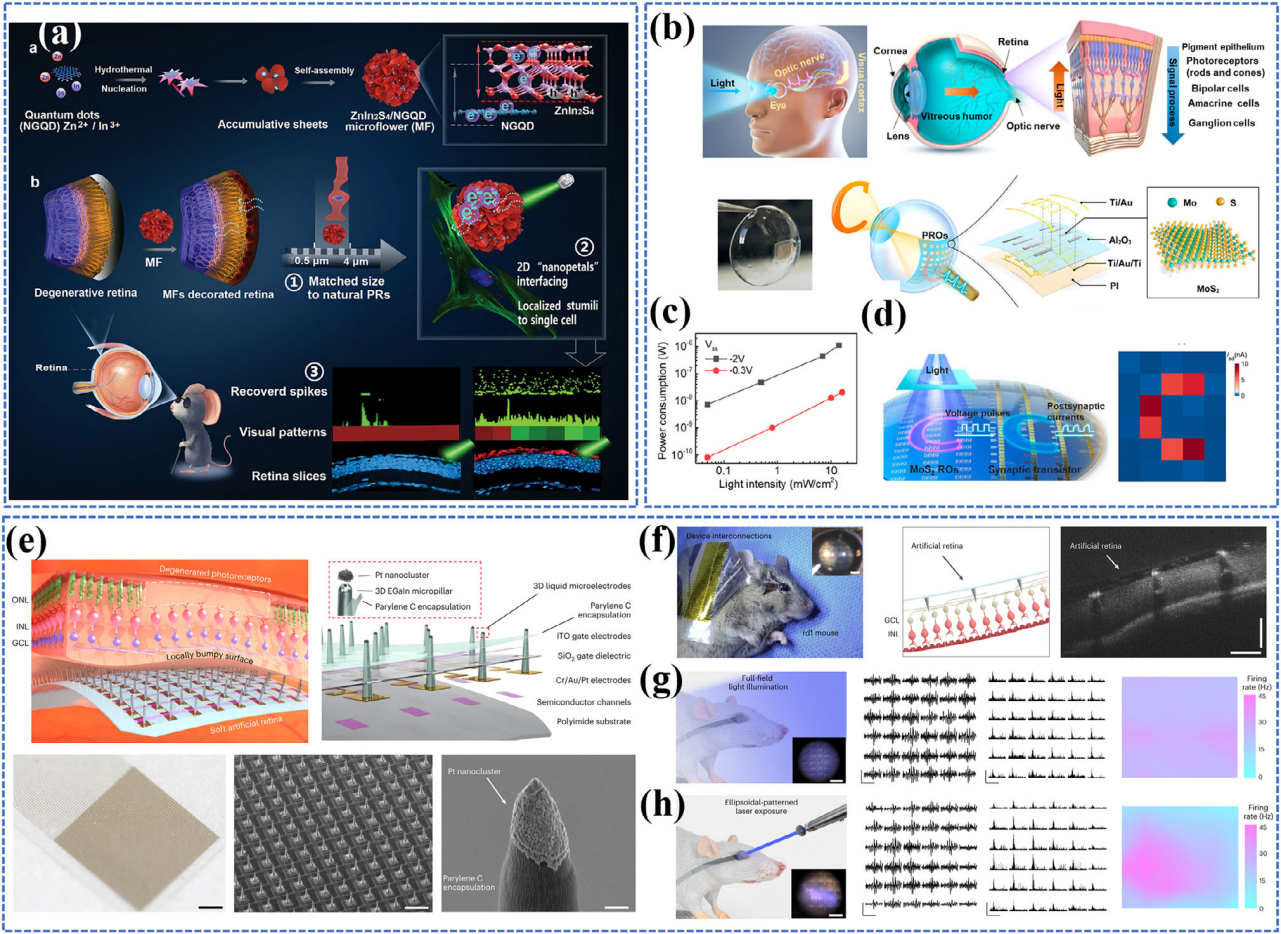
Photoelectric strategy for vision restoration in artificial retinal technologies. a) Retina–ZnIn_2_S_4_/NGQD‐based MF biointerface. Reproduced under terms of the CC‐BY license.^[^ ^32^ ^]^ Copyright 2024 Oudeng et al., published by Wiley‐VCH. b) Illustration of the human visual system and the MoS_2_‐based artificial retina. c) Power consumption of a PRO with VSS = −0.3 V/−2 V as a function of light intensity. d) The letter “C” transmitted and recorded by the postsynaptic currents of synaptic transistors. b–d) Reproduced with permission.^[^ ^391^ ^]^ Copyright 2023, American Chemical Society. e) Soft artificial retina with 3D LM microelectrode arrays. f) Photograph of the in vivo experimental setup using the live rd1 mouse (left), schematic and optical coherence tomography image (right) of the retina of the rd1 mouse after device implantation. g) In vivo experiment under full‐field blue‐light illumination (470 nm, 1.80 mW cm^−2^). h) In vivo animal experiment under continuous laser exposure (415 nm, 1.80 mW cm^−2^) through an ellipsoidal‐patterned shadow mask. e–h) Reproduced under terms of the CC‐BY license.^[^ ^394^ ^]^ Copyright 2024 Chung et al., published by Springer Nature.

First, photo‐triggered nanomaterials can mimic the function of natural photoreceptors. For example, Tang et al. investigated Au‐TiO_2_ nanowire arrays, which exemplify the potential of vertical 1D nanostructures for efficient charge transport and direct photovoltaic conversion without requiring external power sources.^[^ ^388^ ^]^ These nanowire arrays achieved remarkable spatial resolution better than 100 µm and successfully activated primary visual cortex neurons, demonstrating the feasibility of nanoscale approaches in artificial vision. To more effectively simulate natural retinal function, Oudeng et al. developed GQD‐doped ZnIn_2_S_4_ microflowers (MF) with a biomimetic 0D/3D heterostructure photoelectric interface for restoring light response in photoreceptor‐degenerative mice affected by retinitis pigmentosa (**Figure** **20**a).^[^ ^32^ ^]^ The system featured ZnIn_2_S_4_ microflowers with nitrogen‐doped GQDs (NGQDs), forming a heterostructure that enhances visible light absorption and photocurrent conversion efficiency. The MF biointerface exhibited dimensions of 2–5 µm, which closely matched the natural photoreceptors (0.5‐4 µm) and can be effectively dispersed on the curved retinal surface. The soft 2D nanopetals of MF provided extensive surface area for photoelectric activation and flexible cell connection. Under Vis light excitation, charge/hole separation occurred on the ZnIn_2_S_4_/NGQD MFs, generating photocurrent at the biointerface. This process stimulates remaining bipolar cells and transfers electrical signals to retinal ganglion cells (RGCs). The results showed that the MF biointerface successfully restored light responses in seven types of RGCs responsible for brightness coding, with the distribution of responsive cells matching patterns similar to normal mice.

Beyond photodetectors, advancements in optical memory and artificial synapses are revolutionizing artificial vision systems by integrating sensing, memory, and processing into a single device. Meng et al. established a flexible artificial retina perception device based on 2D Janus MoSSe with electronic/ionic and optical co‐modulation capabilities in AI systems.^[^ ^390^ ^]^ This system could mimic biological synaptic plasticity, such as short‐term and long‐term memory, achieving an impressive 83.3% recognition rate for handwritten digits. Similarly, Li et al. developed flexible photoresponsive ring oscillators (PROs) based on 2D MoS_2_ for artificial retina applications.^[^ ^391^ ^]^ As shown in Figure 20b, the device utilizes an array of ring oscillators on flexible substrates as vision pixels, where monolayer MoS_2_ serves as the semiconductor material due to its exceptional photoelectric properties. Under natural light illumination, the PRO pixels generate light‐intensity‐dependent electrical pulses that can be processed and transmitted through the human visual nerve system. The device achieved record‐low power consumption of 12.4 nW per pixel at 10 mW cm^−2^ illumination, approximately 500 times lower than existing silicon‐based devices (Figure 20c). The flexible PRO array successfully demonstrated pattern recognition and information transfer capabilities, capturing and recording the letter “C” at light intensities as low as 0.02 mW cm^−2^ for 500 s (Figure 20d). Liu et al. further developed a hybrid perovskite‐metal‐oxide synapses that integrated optical perception and neuromorphic computing.^[^ ^393^ ^]^ These devices achieved over 90% accuracy in face recognition tasks and introduced novel functionalities, such as passerby filtering via artificial visual persistence, mimicking the selective attention mechanisms of the human retina.

Most recently, Chung et al. developed a soft artificial retina that integrated flexible ultrathin photosensitive transistors with 3D eutectic gallium‐indium alloy (EGaIn) stimulation electrodes for vision restoration in retinal degeneration.^[^ ^394^ ^]^ The system featured a 10 µm‐thick flexible device where photosensitive transistors generate amplified photocurrent upon light stimulation, which is then delivered to retinal neurons through 3D liquid‐metal electrodes (Figure 20e). These EGaIn electrodes, with their low Young's modulus (234 kPa) and liquid form, can conform to the uneven retinal surface while minimizing tissue damage. Their tips were selectively coated with platinum nanoclusters to enhance charge injection capacity (72.84 mC cm^−2^). The device ensured precise neural stimulation, as the 3D electrodes brought the system closer to target cells, while its functional mechanism effectively translated light‐induced photocurrent into neural activation. In vivo experiments using rd1 mice with fully degenerated photoreceptors demonstrated the efficacy of this artificial retina. (Figure 20f). Optical coherence tomography confirmed the structural integrity of the 3D microelectrodes, with no collapse observed across 180 electrodes implanted in five mice. Functional testing showed consistently evoked potentials and uniform retinal responses under 470 nm blue light illumination. (Figure 20g). Furthermore, spatial discrimination capabilities were demonstrated using an ellipsoidal‐patterned 415 nm laser stimulus, where RGCs activity in light‐illuminated regions was approximately four times higher than in dark areas and the spatial distribution of RGCs responses precisely matched the illumination pattern (Figure 20h).

### 3.4 Osteoarthritis (OA)

OA represents the most prevalent degenerative joint disease globally, affecting over 500 million people worldwide and posing a significant healthcare burden.^[^ ^395^ ^]^ Currently, the diagnosis of OA relies heavily on a combination of clinical symptoms, physical examination, and conventional imaging techniques such as X‐ray and MRI, which often detect the disease only after substantial joint damage has occurred.^[^ ^396^ ^]^ Traditional therapeutic approaches primarily focus on symptom management rather than disease modification, typically involving analgesics, non‐steroidal anti‐inflammatory drugs (NSAIDs), intra‐articular injections, and ultimately joint replacement in severe cases.^[^ ^397^ ^]^ However, these conventional treatments have limitations, including systemic side effects, temporary relief, and the inability to halt or reverse disease progression.^[^ ^398^ ^]^ As a result, there is an urgent need for more precise diagnostic tools and innovative therapeutic strategies, particularly those that can provide early detection and targeted treatment. Photo‐triggered therapy represents a cutting‐edge approach to OA treatment. Current advancements focus on designing nanomaterials that respond to specific light (particularly NIR light) to achieve localized imaging, controlled drug delivery, and targeted therapeutic effects, or integrating multifunctionality into these nanomaterials to achieve imaging (e.g., FIL or PAI) guided therapy (e.g., PTT or PDT) on one platform for OA phototheranostics.

#### 3.4.1 Bioimaging

Photo‐triggered imaging modalities are emerging as powerful tools for the diagnosis and monitoring of OA, offering high sensitivity, molecular specificity, and the ability to detect early biochemical and structural changes in joint tissues.

##### PL Imaging

**Figure 21:**
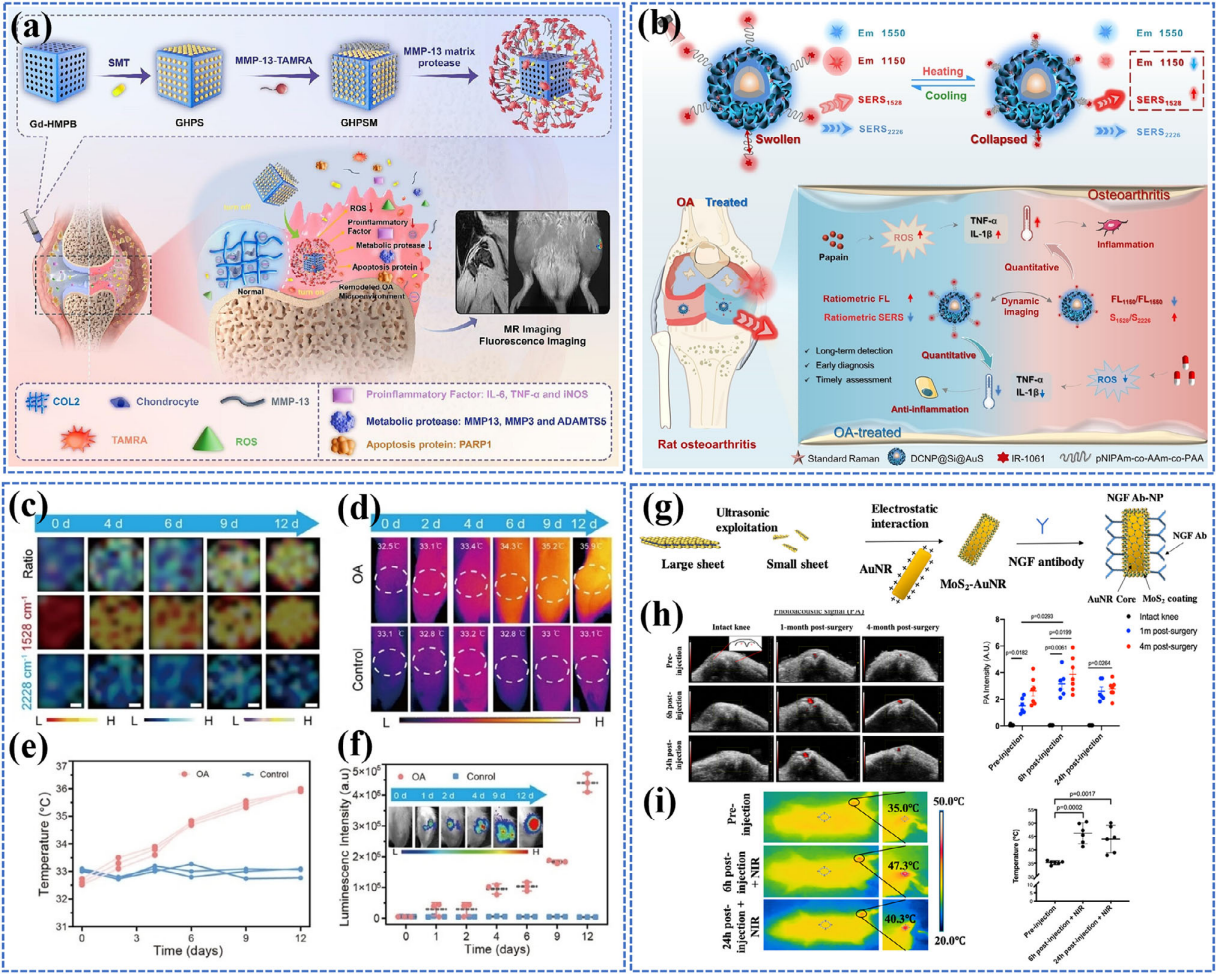
Photo‐triggered bioimaging systems for osteoarthritis diagnosis. a) Schematic illustration of fabrication for GHPSM and its precise diagnosis and treatment as an MMP‐13 responsive nanoprobe for early cartilage degeneration. Reproduced with permission.^[^ ^399^ ^]^ Copyright 2024, Elsevier. b) Schematic illustration of the temperature‐responsive mechanism of DCNP@Si@AuNSP‐PNAP‐I nanoprobe. c) SERS mapping at 1528 cm^−1^ and 2226 cm^−1^, as well as S1528/S2226 images of arthritic knees in rats, scale bar: 2 µm. d) The thermal images of healthy and arthritic knees following the DCNP@Si@AuNSP‐PNAP‐I injection. e) The changes in knee temperature over time in rats with OA and normal knees. f) In vivo luminescence images of arthritic and normal knees in rats at various times following L‐012 injection. b–f) Reproduced with permission.^[^ ^401^ ^]^ Copyright 2024, Wiley‐VCH. g) Construction of anti‐NGF‐MoS_2_‐AuNPs nanocomplex. h) Representative in vivo US–PA overlay images (right) and quantitative PA intensities (right) of intact knees and surgery knees from mice 1 month (*n* = 6) and 4 months (*n* = 10) after surgery at different time points. i) IR thermographs showing an increase of temperature in OA knees after intravenous injection of anti‐NGF‐MoS_2_‐AuNR in 1‐month postsurgery mice (left). Temperature of the OA knees before and 6 h/24 h after injection (right). g–i) Reproduced with permission.^[^ ^403^ ^]^ Copyright 2021, American Chemical Society.

PL imaging in OA research is advancing as a non‐invasive tool to visualize cartilage degradation, inflammation, and key biomarkers with high sensitivity and spatial resolution. Recent developments in nanotechnology have further advanced this technology through the design of nanoprobes that respond to OA‐specific biomarkers. For example, Zhang et al. developed an intelligent responsive nanoprobe, Gd‐HMPB@SMT@MMP (GHPSM), with FLI/MRI and targeted drug delivery capabilities for the diagnosis, monitoring, and treatment of early OA.^[^ ^399^ ^]^ As shown in **Figure** **21**a, the nanoprobe was constructed by encapsulating S‐methylisothiourea (SMT)‐loaded mesoporous Prussian Blue (Gd‐HMPB) with a Tetramethyl Rhodamine (TAMRA)‐labeled MMP‐13 peptide. This design enabled selective accumulation in OA cartilage and specific cleavage of MMP‐13 by overexpressed MMP‐13 protease in the OA microenvironment. This cleavage could activate fluorescence signals for imaging and trigger the controlled release of the loaded SMT and PB, which could reshape the OA microenvironment by scavenging ROS, reducing inflammatory factors (TNF‐α, IL‐6, iNOS), and inhibiting matrix degradation enzymes (MMP‐13, MMP‐3, ADAMTS5). Additionally, the nanoprobe preserved intermediate cartilage structural proteins (e.g., collagen II and aggrecan) and prevented chondrocyte apoptosis by downregulating apoptotic proteins such as PARP1, ultimately delaying cartilage degradation and OA progression. Another application of PL imaging in OA diagnosis is the detection of redox imbalances in the joint microenvironment. Luo et al. developed a mitochondria‐targetable NIR fluorescent probe (NIR‐ClO) with a “turn‐off” fluorescence response for the specific detection and imaging of HClO in OA.^[^ ^400^ ^]^ The probe utilizes a HClO‐triggered C═C bond cleavage reaction, leading to a rapid (<60 s), highly sensitive (LOD: 28.3 nm), and selective fluorescence “on–off” response under physiological conditions. NIR‐ClO successfully visualized HClO levels in living inflammatory cells and an OA rat model.

##### SERS Imaging

SERS imaging leverages the plasmonic enhancement of Raman signals by metallic nanostructures, providing ultra‐sensitive detection of molecular and physiological changes associated with OA. Li et al. developed a temperature‐responsive NIR‐II FL/SERS bimodal imaging nanoprobe (DCNP@Si@AuNSP‐PNAP‐I) for long‐term monitoring of temperature variations and early OA diagnosis (Figure 21b).^[^ ^401^ ^]^ The nanoprobe was constructed by grafting a thermosensitive polymer (pNIPAm‐co‐AAm‐co‐PAA) with an NIR‐II FL dye (IR‐1061) onto nanoporous gold nanoshells. This design allowed the dual detection of FL and SERS signals, modulated by temperature changes in the OA microenvironment. Under 980 nm laser irradiation, temperature changes caused the thermosensitive polymer to approach or move away from the gold nanoshells, altering energy transfer. This resulted in a suppression of the NIR‐II FL signal (FL_1150_/FL_1550_) and amplification of the SERS signal at 1528 cm⁻¹, with Raman signal at 2226 cm⁻¹ serving as an internal calibration standard for precise measurements (Figure 21c). In an OA rat model, the nanoprobe detected a ∼3.22°C temperature difference in inflamed joints compared to normal joints after 12 days (Figure 21d,e). Since temperature changes in inflamed joints often precede other inflammatory markers, such as elevated ROS levels (Figure 21f), this nanoprobe demonstrated significant potential for the early diagnosis of OA. Additionally, it effectively monitored therapeutic responses to COX‐2 inhibitors, highlighting its utility in evaluating treatment efficacy over time.

##### PAI

Unlike traditional imaging methods like MRI or X‐ray, which primarily identify structural damage in advanced OA stages, PAI enables the early detection of biochemical and molecular alterations, such as inflammation and cartilage degeneration, which are critical for timely intervention. For example, Shen et al. engineered Au@PDA‐WL NPs to visualize articular cartilage through collagen II targeting.^[^ ^402^ ^]^ The nano‐probe was fabricated by coating AuNPs with PDA and immobilizing the collagen II‐targeting WYRGRL peptide on the particle surface. After injection, these nanoparticles specifically target collagen II on the surface of articular cartilage, and their aggregation induces a localized plasmon resonance coupling effect, enabling clear visualization of cartilage degradation patterns and enhancing PAI signal intensity. Additionally, the catechol groups in the PDA shell scavenged ROS produced by chondrocytes, alleviating oxidative stress and delaying OA progression. Similarly, Au et al. developed an NGF‐targeted molecular theranostic nanoprobe (anti‐NGF–MoS_2_–AuNR) for precise pain imaging‐guided PTT in OA joints.^[^ ^403^ ^]^ The nanoprobe was constructed by coating AuNRs with 2D MoS_2_ nanosheets to enhance photothermal conversion efficiency and photothermal stability, followed by conjugation with an NGF monoclonal antibody to actively target peripheral OA pain (Figure 21g). Upon intravenous injection, the nanoprobes selectively accumulated in the inflamed synovium of OA joints, as confirmed by PAI at 710 nm, with no signal observed in intact knees (Figure 21h). PTI further showed a localized temperature increase in OA knees upon 808 nm laser irradiation (0.2 W cm^−2^ for 10 min), indicating precise treatment of inflamed tissues (Figure 21i). Additionally, the development of dual‐modal imaging nanoprobes has enabled more precise OA diagnosis and monitoring. Li et al. developed a dual‐ratiometric core–satellite nanoprobe with SERS and PAI capabilities to monitor H₂O₂ levels in OA joints in real time.^[^ ^404^ ^]^ The nanoprobe consisted of mesoporous silica‐coated gold nanogapped nanorods (AuNNRs) conjugated with AuNPs modified with 4‐mercaptobenzoboric acid and loaded with ABTS and horseradish peroxidase (HRP). In the OA microenvironment, the high concentration of H₂O₂ triggered the dissociation of AuNPs, leading to the reduction of SERS signal at 2228 cm⁻¹ and enhancement of PA signal at 750 nm while maintaining stable internal signals (SERS at 1418 cm⁻¹ and PA at 1250 nm). In a rabbit model of knee OA induced by papain, the nanoprobe successfully detected elevated H₂O₂ levels in the diseased joint, differentiated between healthy and inflamed tissues, and tracked the progression of OA in real‐time using SERS imaging and PAI. This dual‐modal approach highlights the potential for precise, non‐invasive diagnostics and therapeutic monitoring in OA.

#### 3.4.2 Multimodality Therapy

##### PTT

PTT is emerging as a promising approach in OA treatment, which can be combined with other therapeutic mechanisms, such as ROS scavenging, drug delivery, and immunomodulation, to address multiple challenges in OA, including inflammation,^[^ ^405^, ^406^ ^]^ pain,^[^ ^403^, ^407^ ^]^ chondrocyte degeneration,^[^ ^408^ ^]^ and cartilage destruction.^[^ ^409^ ^]^

**Figure 22:**
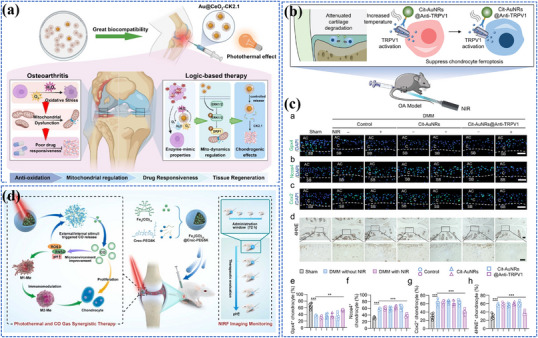
Phototherapy for osteoarthritis. a) Mechanism of the Au@CeO_2_‐CK2.1 yolk‐shell nanozymes in alleviating inflammation and enhancing chondrogenic drug response to combat osteoarthritis by combining the antioxidant, mitochondrial regulation, and chondrogenic effects. Reproduced with permission.^[^ ^406^ ^]^ Copyright 2024, Elsevier. b) Illustration of Cit‐AuNRs@Anti‐TRPV1 switch for photothermal activation of TRPV1 signaling to attenuate OA. c) Cit‐AuNRs@Anti‐TRPV1+NIR plays an anti‐ferroptotic role in the mice OA model. b,c) Reproduced under terms of the CC‐BY license.^[^ ^416^ ^]^ Copyright 2024 Li et al., published by Wiley‐VCH. d) Schematic illustration of Fe_3_(CO)_12_ @Croc‐PEG5K for NIRF imaging‐guided photothermal and CO gas synergistic therapy of OA. Reproduced with permission.^[^ ^418^ ^]^ Copyright 2023, Elsevier.

ROS plays a significant role in the pathogenesis of OA by contributing to oxidative stress, inflammation, and cartilage degradation, which has driven the development of PTT systems with ROS management capabilities. Nanozymes with catalytic activity have gained attention in PTT‐based OA therapy due to their ability to mimic natural antioxidant enzymes such as superoxide dismutase (SOD) and catalase (CAT). Shi et al. developed biomimetic molybdenum nanodots with the synergistic effects of ROS/RNS scavenging and NIR‐induced mild PTT to treat OA. This dual functionality not only protected cartilage but also promoted subchondral bone regeneration in an OA mouse model.^[^ ^405^ ^]^ Similarly, Li et al. introduced multifunctional Au@CeO_2_ yolk‐shell nanozymes (YSNs) loaded with the bioactive peptide CK2.1. The CeO₂ shell provided ROS‐scavenging activity, while the Au core enabled photothermal effects.^[^ ^406^ ^]^ Under NIR irradiation (635 nm, 0.8 W cm^−2^), these nanozymes neutralized excessive ROS, restored mitochondrial homeostasis, and regulated mitochondrial dynamics by inhibiting the ERK1/2‐DRP1 phosphorylation pathway (**Figure** **22**a). Additionally, the system achieved controlled release of the chondrogenic peptide under NIR, significantly enhancing cartilage repair in vivo. Other nanozyme systems have shown similar success. Mn_3_O_4_@PDA@Pd‐SS31 and Pt SA/C_3_N_4_ nanozymes demonstrated enhanced SOD/CAT‐mimicking activity combined with PPT under NIR irradiation.^[^ ^410^, ^411^ ^]^ Li et al. enhanced SOD/CAT‐like activity and achieved 49.1% photothermal efficiency with Pd/CoPcS‐Ti_3_C_2_T_x_ nanozymes using ultrafine Pd clusters.^[^ ^412^ ^]^ Yang et al. improved catalytic performance and reached 55.41% photothermal efficiency with PtCuOX/CeO_2_‐X nanozymes through bimetallic Cu and Pt integration.^[^ ^413^ ^]^ Additionally, the integration of PTT with hydrogel technology has created multifunctional treatment platforms for OA. He et al. developed an injectable hyaluronic acid‐thiourea‐Cu^2+^ (HSC) hydrogel with photothermal and antioxidant properties.^[^ ^408^ ^]^ The hydrogel utilized dynamic and reversible chelation between thiourea groups (NCSN) and Cu^2^⁺ ions, providing excellent photothermal conversion, injectability, self‐healing capabilities, and ROS scavenging capabilities. Upon NIR light exposure (808 nm), the HSC/NIR hydrogel mitigates inflammation, promotes cartilage anabolism, and suppresses catabolism by modulating the JAK/STAT signaling pathway and macrophage polarization. Both in vitro and in vivo studies demonstrated its ability to reduce cartilage degeneration, synovitis, osteophyte formation, and subchondral sclerosis.

Beyond ROS management, PTT has been utilized for precise therapeutic delivery and controlled release. Qiao et al. designed a light‐responsive gold nanocage‐based delivery system (AuDPNAs) coated with poly(β‐amino‐ester) and phase‐change materials for co‐delivering diacerein (DIA) and NGF‐targeting siRNA.^[^ ^414^ ^]^ This system enabled the controlled release under NIR irradiation, providing anti‐inflammatory effects, pain relief, and protection against chondrocyte apoptosis through HSP70 upregulation, ultimately improving joint repair. Xu et al. developed a NIR‐sensitive tea polyphenol‐modified zirconium‐based porphyrin MOF (TP‐Au@PCN) nanoplatform with photocatalytic and photothermal properties for repairing mitochondrial function and improving the osteoarthritic microenvironment.^[^ ^415^ ^]^ Upon NIR irradiation, the nanoplatform raised the joint temperature to 46.9 °C, triggering controlled drug (TP) release, alleviated oxidative stress, and reduced chondrocyte apoptosis, thus offering a multifunctional and synergistic therapeutic strategy for OA with antimicrobial and cartilage‐regenerative properties. Additionally, receptor‐targeted approaches have shown promise. Li et al. developed a photothermal switch by conjugating citrate‐stabilized AuNRs (Cit‐AuNRs) with transient receptor potential vanilloid 1 (TRPV1) monoclonal antibody (Cit‐AuNRs@Anti‐TRPV1) for targeted activation of TRPV1 in chondrocytes to attenuate OA progression (Figure 22b).^[^ ^416^ ^]^ The Cit‐AuNRs@Anti‐TRPV1 system utilized the thermal sensitivity of TRPV1, where NIR irradiation induced localized heating, activating TRPV1 to suppress chondrocyte ferroptosis. In Figure 22c, GPX4^+^ chondrocytes decreased after DMM surgery, while Ncoa4, COX2, and 4HNE levels increased significantly. After treatment with Cit‐AuNRs@Anti‐TRPV1 under NIR irradiation (880 nm, 0.75 W cm^−2^), GPX4 expression could be restored, while Ncoa4, COX2, and 4HNE levels decreased, demonstrating its chondroprotective effects by inhibiting ferroptosis. In vivo studies demonstrated that Cit‐AuNRs@Anti‐TRPV1 with NIR irradiation significantly reduced cartilage degeneration, osteophyte formation, and subchondral bone sclerosis while improving physical activity and alleviating pain in the destabilization of OA mice.

##### Phototheranostics

Phototheranostics offers a cutting‐edge solution for precise OA diagnosis and personalized treatment by integrating light‐responsive nanomaterials into imaging‐guided therapeutic platforms. For example, Ruan et al. developed a dual‐responsive hybrid micelle system (DHMP/M) with a melanin core and polydopamin shell for PAI‐guided therapy.^[^ ^417^ ^]^ This system enabled ROS‐controlled long‐term release and NIR‐triggered short‐burst release of melanin, effectively scavenging ROS in the OA microenvironment. The DHMP/M system exhibited excellent biocompatibility, stability, and therapeutic efficacy, with significant chondroprotective effects via TLR‐2/MAPK/Akt signaling pathway inhibition in vitro and in vivo. Recently, advanced gas‐therapy approaches have further expanded the possibilities of phototheranostic platforms. Gao et al. developed the first intelligent cascade‐responsive nano gas tank, Fe_3_(CO)_12_ @Croc‐PEG5K, which combines photothermal and CO gas release capabilities for NIR FLI‐guided OA therapy (Figure 22d).^[^ ^418^ ^]^ This system encapsulates Fe₃(CO)₁₂ within a hydrophilic and hydrophobic Croc‐PEG5K shell, enabling photothermal‐triggered CO release under 808 nm irradiation (1 W cm^−2^, 16 min). The released CO can scavenge ROS/RNS, normalize pH, alleviate inflammation, and protect cartilage integrity. Furthermore, its pH‐sensitive NIR FLI capability enables real‐time monitoring of the pathological microenvironment, thereby optimizing the therapeutic window. Luan et al. developed a ROS‐sensitive upconversion nanocomposite hydrogel (UCNP/VitroGel) for OA treatment.^[^ ^419^ ^]^ The hydrogel was constructed using Er‐doped UCNPs coated with mesoporous silica (UCNP@mSiO_2_) and functionalized with a ROS‐sensitive thioketal (TK) linker, a fluorescence quencher (BHQ), and β‐cyclodextrin (β‐CD) as a gatekeeper for drug release. The drug kartogenin (KGN), which induces chondrogenic differentiation of mesenchymal stem cells (MSCs), was loaded into the nanocomposite. When exposed to ROS (e.g., H2O2) in the OA environment, the TK linker ruptured, releasing BHQ to restore UCNP fluorescence for ROS detection and detaching β‐CD to release KGN for cartilage repair. This dual‐function system allowed real‐time diagnosis of OA through fluorescence recovery under 980 nm NIR light (0.2 W cm^−2^) and simultaneous therapeutic intervention by promoting MSC differentiation, demonstrating its potential as a powerful tool for integrated OA management.

### 3.5 Comparison of Photo‐Energy Conversion‐Based Technologies Across Diseases

The demands for photo‐triggered biosensing, bioimaging, and phototherapy vary significantly across diseases such as cancer, AD, retinal degeneration, and OA due to their distinct pathological features and therapeutic goals (**Table** **3**). In cancer, the need for early diagnosis requires biosensing techniques with ultra‐high sensitivity to detect minute concentrations of biomarkers like circulating tumor cells or DNA. Additionally, high‐resolution bioimaging is critical for visualizing tumor margins, detecting metastases, and guiding surgical interventions. Phototherapy in cancer demands selective and localized treatments, which can overcome challenges like tumor heterogeneity, hypoxia, and multidrug resistance. Similarly, AD requires biosensing systems capable of detecting low‐abundance biomarkers such as Aβ plaques and tau tangles during early disease stages. Bioimaging approaches must penetrate the BBB to visualize small Aβ aggregates and neuroinflammatory changes while remaining non‐invasive. Phototherapy in AD focuses on addressing protein aggregation, oxidative stress, and chronic neuroinflammation, requiring precise, BBB‐penetrating approaches. Retinal degeneration emphasizes precise layer targeting within the delicate eye structure. Osteoarthritis requires bioimaging to monitor core joint structures and phototherapy to attenuate inflammation and pain.

**Table 3:** Comparison of photo‐energy conversion‐based technologies across cancer, AD, retinal degeneration, and OA.

Diseases	Key demands	Specific challenges	Photo‐energy‐conversion based solutions	Advantages	Limitations	Refs.
Cancer	Early diagnosis	Heterogeneous biomarkers, low marker abundance, complex biological matrices	PL‐based biosensing:	High sensitivity, real‐time monitoring, multiplexed detection	Standardization challenges	[90, 197, 198, 199, 200, 201, 202, 203, 204, 206]
			SERS‐based biosensing	Ultra‐sensitive, molecular fingerprinting, label‐free, minimal photobleaching, multiplexing capability, label‐free detection options	Complex instrumentation, reproducibility issues, limited in vivo translation, complex data analysis	[210, 211, 212, 213, 218, 219, 220, 221, 222, 223, 224, 225, 226]
			PEC‐based biosensing	Label‐free, low background, miniaturization potential	Electrode fouling, limited multiplexing, narrower biomarker range	[230, 231, 232, 233, 234, 235, 236, 237, 238]
	Tumor visualization	Deep tissue limits, real‐time surgical needs, tumor margin definition, metastatic mapping	PL imaging	High resolution, real‐time imaging, FDA‐approved agents	Limited depth, potential toxicity	[100, 107, 108, 239, 240, 241, 242, 244]
			SERS imaging	Molecular specificity, multiplexing, minimal photobleaching, high signal‐to‐background ratio	Limited penetration, slower acquisition, lack of clinical‐grade probes	[246, 247, 248, 249, 250, 251, 252, 253, 254, 255]
			PAI	Deeper penetration (cm), functional information; no ionizing radiation, oxygenation mapping	Lower sensitivity than PL	[38, 160, 161, 162, 256, 257, 258, 259, 260, 262, 263, 264, 265, 266, 268, 269, 270, 271, 272, 273]
	Effective treatment	Superficial tumor: cosmetic outcomes recurrence field cancerization	PDT	Excellent cosmetic result, non‐invasive, multiple lesions at once, repeatable without resistance	Limited depth (2‐3 mm), treatment pain; photosensitivity period, not for pigmented lesions	[285, 286, 287, 288, 289, 290]
		Deep tumors: Accessibility, hypoxia, treatment resistance, lack of guidance	PTT, PDT, or phototheranostics	Effective in hypoxic conditions, rapid, image‐guided treatment, minimal invasiveness	Heat diffusion issues, potential collateral damage, thermotolerance in tumors	[27, 28, 34, 68, 291, 292, 293, 294, 295, 296, 297, 298, 299, 300]
AD	Early diagnosis	Low abundance of early AD makers, lack of reliable early biomarkers, heterogeneous presentation, long prodromal phase	PL‐based biosensing:	High sensitivity for early‐stage AD biomarker, real‐time monitoring of aggregation	Limited biomarker validation	[318, 319, 320, 321, 322]
			SERS‐based biosensing	Ultra‐sensitive, molecular fingerprint	Complex instruments, reproducibility difficulties, limited in vivo application,	[322, 324, 325, 326, 327, 328, 329, 330, 331]
			PEC‐based biosensing	Label‐free; wearable potential; continuous monitoring	Electrode stability, limited biomarker panels	[314, 315, 332, 333, 334, 335, 336, 337]
	Brain visualization	Difficulty in crossing the BBB, monitoring early‐stage protein aggregation, chronic inflammation	PL imaging	High‐resolution, real‐time tracking of Aβ and Tau aggregation, early‐stage biomarker detection	Limited depth penetration, autofluorescence, photobleaching, potential cytotoxicity	[91, 338, 342, 343, 344, 345]
			SERS imaging	ultrasensitive, molecularly specific detection of AD biomarkers, real‐time monitoring of Aβ aggregation	Limited depth penetration, complex nanoprobe design, potential toxicity	[346, 347, 348]
			PAI	Deep‐tissue imaging; monitoring brain hemodynamics and vascular dysfunction	Lower molecular specificity, dependence on contrast agents, lower sensitivity	[349, 350]
	Disease modification	BBB challenges, chronic neuroinflammation, protein misfolding/aggregation, progressive neuronal loss	PDT	Aβ/tau clearance, reduces neuroinflammation, repeatable treatments	Limited penetration through skull, photosensitizer delivery challenges, ROS overproduction	[356, 357]
			PTT	Enhance BBB permeability, disassembles Aβ/tau aggregates	Potential neurotoxicity from heat, precise targeting challenging	[359, 360, 361, 362, 363, 364, 365, 366]
Retinal Degeneration	Targeted treatment	Precise layer targeting, photoreceptor/RPE death, abnormal neovascularization, geographic atrophy	PDT	Minimally invasive, selective targeting of abnormal vessels, potentially reduces oxidative stress and inflammation	Risk of collateral retinal damage, limited therapeutic depth, retinal photosensitivity	[381]
			Photoelectric stimulation:	Potential restoration of visual function; direct electrical stimulation of retinal neurons, independent of exogenous chemicals	Long‐term implant biocompatibility, device lifespan, potential inflammatory response, surgical implantation necessary	[32, 388, 390, 391, 392, 393, 394]
OA	Early Detection & Monitoring	Low‐biomarker concentration, deep visualization needs	PL imaging	Early detection of biochemical changes (e.g., cartilage degradation)	Autofluorescence	[399, 400]
			SERS imaging	Detection of multiple molecular biomarkers at ultralow concentrations	Low penetration, signal variability,	[401]
			PAI	Monitoring deep joint structures, vascularization, and inflammation	Lower spatial resolution	[402, 403, 404]
	Pain Management & Joint Preservation	Joint space accessibility, chronic inflammation, cartilage degradation, pain management, maintaining mobility	PTT	Localized heat for pain relief, cartilage protection, precise targeting, minimal invasiveness;	Risk of thermal injury to surrounding tissues, limited penetration depth in larger joints, potential heat diffusion issues	[405, 406, 407, 408, 409, 410, 411, 412, 413, 414, 415, 416]

Photo‐triggered biosensing, bioimaging, and therapy provide unique solutions to these disease‐specific demands. In cancer, PL, SERS, and PEC biosensing offer ultra‐sensitive detection of biomarkers in complex biological matrices. Imaging techniques like PL and SERS imaging visualize tumor heterogeneity and margins with high precision, and PAI overcomes tissue penetration challenges to provide functional and structural insights into deeper tumors. Phototherapy techniques like PDT selectively generate ROS to kill tumor cells in superficial areas, while PTT uses localized heat to treat hypoxic or resistant tumors. Combined PDT/PTT approaches further enhance efficacy by addressing tumor complexity. In AD, PL and SERS biosensing detect Aβ and tau biomarkers with high sensitivity, while PEC biosensing facilitates real‐time, label‐free monitoring in wearable or implantable systems. Imaging techniques like PL and SERS imaging detect Aβ plaques and tau tangles with high resolution and molecular specificity, while PAI provides deeper tissue penetration to visualize neurovascular dysfunction and inflammation. Phototherapy approaches such as PDT reduce protein aggregation and neuroinflammation, while PTT transiently disrupts the BBB to enhance drug delivery and disassemble aggregates. Retinal degeneration benefits from PDT for effectively targeting abnormal blood vessels and photoelectric stimulation for restoration visual function by activating surviving retinal neurons. For osteoarthritis, PL imaging detects early biochemical changes in cartilage, while PAI visualizes deep joint inflammation and vascularization. Phototherapy approaches like PTT reduce inflammation and pain through localized heat, protecting cartilage from further degradation. Overall, these light energy conversion‐based technologies enable precision theranostics tailored to the specific pathological features of each disease.

## 4 Current Challenges and Future Directions

Photo‐energy conversion‐based nanomaterials represent a transformative approach in modern medicine, offering precise spatial and temporal control over therapeutic interventions through controlled light activation. Despite their remarkable potential in disease diagnosis and treatment, several significant challenges remain in their clinical translation, necessitating systematic, interdisciplinary advancements. Below, we delve deeper into these challenges and propose advanced future directions.

### 4.1 Clinical Translation Stages and Remaining Challenges

Photo‐energy conversion‐based technologies, including PDT, PTT, and photoelectric implants, have demonstrated considerable clinical potential due to their selective activation, minimal invasiveness, and precise spatiotemporal control. Notably, cancer and ophthalmology represent the most advanced fields for clinical translation, whereas AD and OA remain in their infancy.

In oncology, PDT has achieved the most substantial clinical adoption among photo‐triggered technologies. As shown in **Table** **4**, established photosensitizers such as Porfimer Sodium (Photofrin),^[^ ^420^ ^]^ Aminolevulinic Acid (Levulan),^[^ ^421^ ^]^ and Methyl Aminolevulinate (Metvix)^[^ ^422^ ^]^ are FDA‐approved and widely used in treating esophageal, lung, bladder, and skin cancers, as well as actinic keratosis (AK) and basal cell carcinoma (BCC). Temoporfin (Foscan)^[^ ^423^ ^]^ and Padeliporfin (Tookad soluble)^[^ ^424^ ^]^ have received EMA approval for advanced head and neck cancer and prostate cancer, respectively, while Talaporfin Sodium (Laserphyrin, NPe6)^[^ ^425^ ^]^ has been approved in Japan for lung cancer treatment. However, the clinical effectiveness of PDT in deep‐seated tumors remains limited by insufficient tissue penetration of conventional visible and NIR‐I photosensitizers and the hypoxic tumor microenvironment, which restricts the generation of ROS. Emerging PDT agents such as Verteporfin (Visudyne), TLD‐1433 (Rutherrin), and Redaporfin (LUZ11) are currently undergoing clinical trials to address these issues, though rigorous validation is still needed. Despite these advancements, current photosensitizers used in PDT still present challenges, including prolonged retention in tissues, off‐target effects, and phototoxicity. On the other hand, PTT is at an earlier stage of clinical development compared to PDT. Noble metal nanoparticles‐based PTT agents, like gold nanoshells (AuroShell® and AuroLase®), have shown promising results in Phase I/II trials for cancers, such as prostate and head and neck cancers. However, concerns about long‐term safety, biodistribution, and clearance of nanoparticles are still crucial for broader clinical adoption. Additionally, FDA‐approved agents like ICG^[^ ^426^ ^]^ and Pafolacianine^[^ ^427^ ^]^ are widely used for imaging and tumor localization rather than direct therapy. These agents take advantage of NIR absorption (≈780–808 nm) to achieve deep tissue penetration, making them valuable tools for surgical guidance. Emerging agents such as Panitumumab‐IRDye800 (Phase I/II) and ZW800‐1 (Phase II) are under early clinical investigation, while carbon nanoparticles, which are now entering Phase III clinical trials as surgical guides, hold significant promise for translation into routine cancer care.

**Table 4:** Clinical translation stages of photo‐energy conversion‐based technologies.

Diseases	Photo‐therapy	Phototherapy agents	Class	Wavelength	Clinical status	Indications	Refs.
Cancer	PDT	Porfimer Sodium (Photofrin®)	Porphyrin	630 nm	FDA‐approved	Oesophageal, lung, bladder, brain, head and neck cancer	[420]
		Aminolevulinic Acid (ALA, Levulan®)	Porphyrin precursor	417‐635 nm	FDA‐approved	Skin cancers (AK)	[421]
		Methyl Aminolevulinate (MAL, Metvix®, Metvixia®)	Porphyrin precursor	570‐670 nm	FDA‐approved	Skin cancers (AK, BCC)	[422]
		Temoporfin (Foscan®)	Chlorin	652 nm	EMA‐approved	Advanced head and neck cancer	[423]
		Padeliporfin (Tookad® soluble, WST11)	Bacteriochlorophyll derivative	753 nm (NIR)	EMA‐approved	Prostate cancer	[424]
		Talaporfin Sodium (Laserphyrin®, NPe6)	Chlorin	664 nm	Japan‐approved	Lung cancer	[425]
		Verteporfin (Visudyne®)	Benzoporphyrin derivative	689 nm	Phase I/II	Breast, pancreatic cancer	NCT02872064, NCT02939274, NCT03033225
		TLD‐1433 (Rutherrin®)	Metal‐organic compound	525 nm	Phase II	Bladder cancer	NCT03945162
		Redaporfin (LUZ11)	Bacteriochlorin derivative	749 nm (NIR)	Phase I/II	Head and neck cancer	NCT02070432
	PTT	Gold nanoshells (AuroShell®)	Noble metal nanoparticle	∼800 nm (NIR)	Phase II	Prostate cancer	NCT04240639
		Gold nanoshells (AuroLase®)	Noble metal nanoparticle	∼800 nm (NIR)	Phase I	Head and neck cancer	NCT00848042
	PL	Indocyanine Green (ICG)	Cyanine dye	780‐808 nm (NIR)	FDA approved (for imaging)	Various cancer	[426]
		Pafolacianine	Folate receptor dye	774 nm	FDA‐approved	Ovarian cancer, lung cancer	[427]
		Panitumumab‐IRDye800	Organic dye	774 nm	Phase I/II	Head and neck, lung cancer	NCT03405142;NCT03582124
		ZW800‐1	Zwitterionic dye	776 nm	Phase II	Pancreatic, oral, head and neck cancer	NCT05518071;NCT04191460;NCT05752149
		Carbon nanoparticle	Carbon	/	Phase III	Thyroid, breast cancer	NCT06791005;NCT04951245
Retinal degeneration	PDT	Verteporfin (Visudyne®)	Benzoporphyrin derivative	689 nm	FDA‐approved for AMD;	AMD	NCT01846273
	Photoelectric strategy	Bionic Vision Systems (e.g., PRIMA system by Pixium Vision)	Photovoltaic subretinal implant	850 nm	Clinical trials	AMD	NCT03392324; NCT04676854
AD	NIR light therapy	/	/	Not specific	Early Phase 1	AD	NCT06836180
		/		Not specific (300 mW cm^−2^, 11 min)	Phase 2	AD	NCT04784416
OA	Low‐/high‐intensity laser therapy	/	/	Not specific	N.A.	OA	NCT06825767

In ophthalmology, Verteporfin (Visudyne) has successfully been approved by the FDA for AMD treatment. Other innovative approaches such as the PRIMA system by Pixium Vision, which uses photovoltaic subretinal implants to stimulate surviving retinal neurons to restore vision, are currently in clinical trials for AMD treatment. The accessibility of the retina to light‐based interventions further enhances the translational potential of these technologies in ophthalmology. However, their long‐term biocompatibility and stable integration within retinal tissue present significant clinical hurdles. For neurological diseases such as AD and chronic degenerative diseases like OA, clinical translation remains challenging, primarily due to the difficulty in noninvasively penetrating deep tissues such as the brain and joints. Current clinical trials investigating near‐infrared light or laser therapy (e.g., NCT06836180 and NCT04784416 for AD, NCT06825767 for OA) demonstrate the growing interest in light therapy but also highlight the need for more efficient light‐activated platforms that can achieve targeted penetration of deep tissues and improve treatment outcomes.

Despite the promising high energy conversion efficiency and therapeutic potential of many nanomaterials, only a small fraction has successfully transitioned to clinical applications. This underscores several critical barriers, including the challenges of scaling up laboratory‐synthesized nanomaterials for industrial production, maintaining stability during storage, ensuring cost‐effectiveness, and addressing potential biological toxicity. Furthermore, regulatory and standardization issues pose significant obstacles. Current photo‐triggered platforms often lack standardized protocols for light dosage, wavelength selection, and treatment duration, which hampers broad clinical implementation and consistent therapeutic outcomes. Addressing these challenges is essential to fully realize the potential of light‐triggered nanomaterials in clinical settings.

### 4.2 Future Perspectives

Looking toward the future, the development and application of photo‐triggered nanomaterials offer transformative potential across multiple disease areas, but addressing the current challenges requires innovative, interdisciplinary approaches. Below, we outline several advanced directions explicitly connected to enhancing photo‐energy conversion efficiency and therapeutic outcomes.

1. Advanced long‐wavelength photosensitizers and light delivery techniques: To overcome the limitations of tissue penetration in phototherapy, the development of longer‐wavelength photosensitizers, particularly in the NIR‐II range, remains a critical priority. Recent breakthroughs in AIEgens, metal nanoclusters, 2D nanomaterials, and hybrid nanomaterials have demonstrated efficient NIR‐II activation capabilities, and deeper tissue penetration, reduced scattering, and minimal phototoxicity in preclinical studies. Additionally, multiphoton excitation,^[^ ^428^ ^]^ fiber‐optic probes,^[^ ^429^ ^]^ and nanoscale UCNPs^[^ ^108^, ^287^ ^]^ have emerged as advanced methods to deliver and convert photon energies efficiently, enabling therapeutic activation at greater tissue depths without invasive procedures.
2. Overcoming hypoxia and enhancing conversion efficiency via smart delivery strategies: Hypoxia in solid tumors significantly limits ROS generation during PDT. Developing oxygen‐generating nanoparticles (e.g., catalase‐loaded nanoplatforms)^[^ ^242^, ^430^ ^]^ and hypoxia‐tolerant photosensitizers (e.g., metal–organic frameworks^[^ ^431^ ^]^ or AIE molecules)^[^ ^286^ ^]^ could directly enhance photo‐energy conversion efficiency in hypoxic environments. Moreover, biomimetic delivery systems (e.g., cell membrane‐coated nanoparticles)^[^ ^242^, ^259^ ^]^ can modulate the local tumor microenvironment by improving oxygenation, thus directly influencing photodynamic efficiency and minimizing collateral tissue damage.
3. Bacteria‐based platforms advancing precision medicine beyond passive cell membrane coating: Unlike cell membrane‐based coatings, which passively mimic surface properties of source cells for immune evasion and targeting, bacteria‐based platforms are inherently dynamic and active.^[^ ^432^ ^]^ Engineered bacteria can autonomously respond to the tumor microenvironment, addressing limitations like hypoxia through photosynthetic oxygen generation and modulating metabolic pathways to overcome immunosuppression.^[^ ^433^ ^]^ Additionally, bacteria can actively home to tumors, utilizing their natural affinity for hypoxic and acidic environments. When combined with photosensitizers and nanomaterials, bacteria‐based platforms enable precise and multifunctional therapies, significantly improving photothermal and photodynamic therapeutic outcomes.^[^ ^434^ ^]^ While cell membrane‐based methods excel in biocompatibility, immune evasion, and stability, they lack the active and adaptive therapeutic capabilities of bacteria. Future advancements in bacteria‐based systems, such as improved biosafety and genetic engineering, will further distinguish them as powerful tools for next‐generation precision medicine.
4. AI and machine learning to optimize therapeutic platforms: AI and machine learning approaches are transforming the design, optimization, and application of photo‐energy conversion nanomaterials. Recent developments include machine learning‐driven materials discovery platforms that rapidly identify optimal photothermal and photodynamic agents based on predicted quantum yields, photostability, and biocompatibility.^[^ ^435^, ^436^ ^]^ AI‐assisted imaging techniques, including advanced spectral analysis of photoacoustic and SERS data, significantly improve diagnostic precision and accuracy.^[^ ^212^, ^222^, ^331^ ^]^ Furthermore, real‐time AI‐driven treatment planning offers breakthroughs in precision oncology by enhancing tumor characterization, enabling personalized dosimetry, optimizing treatment protocols, and predicting adverse events.^[^ ^437^, ^438^ ^]^ Together, these innovations represent a paradigm shift toward more effective and personalized therapeutic approaches.
5. Quantum computing for advanced materials design and optimization: Quantum computing represents an emerging frontier capable of modeling complex quantum mechanical properties beyond classical computational limits.^[^ ^439^ ^]^ Recent quantum algorithms have demonstrated practical applications in materials science, successfully simulating complex phenomena like spin‐orbit coupling and low‐energy bands in 2D materials (e.g., TMDs).^[^ ^440^ ^]^ Quantum machine learning approaches applied to perovskite materials have achieved ≈87% identification accuracy through hybrid classical‐quantum models, highlighting its potential for scalable materials discovery as quantum hardware develops.^[^ ^441^ ^]^ The continued development of quantum computational methods promises to revolutionize materials discovery and optimization in photo‐triggered nanomedicine.
6. Exploring synergistic combinations with other remote energy conversion: Combining photo‐triggered theranostics with other remote energy conversion techniques enhances precision in targeting and activating therapeutic agents, leading to more effective treatments with reduced side effects.^[^ ^442^, ^443^, ^444^ ^]^ Synergistic approaches could be particularly beneficial in complex diseases that involve multiple pathological processes.
7. Optimizing phototherapy and standardization efforts: Precise control of light intensity, wavelength, and exposure time is crucial for optimizing phototherapy, as these factors directly impact efficacy, safety, and outcomes. Uniform light delivery, achieved by adjusting the distance between the source and target, ensures accurate dosing and maximizes effectiveness. Selecting the appropriate wavelength based on the photosensitizer and tissue depth prevents unintended damage while ensuring effective results. Modern phototherapy devices with features like digital timers, automated systems, and advanced technologies such as programmable light devices and real‐time feedback systems further enhance precision and safety. Additionally, efforts to create standardized clinical guidelines will promote wider adoption and ensure consistent, reliable treatment outcomes across different patient populations.

## 5 Conclusion

In conclusion, photo‐energy conversion‐based technologies hold immense potential to revolutionize the diagnosis and treatment of cancer, neurodegenerative diseases, retinal degeneration, and osteoarthritis, offering unprecedented precision and multifunctionality. By addressing current challenges such as biocompatibility, light penetration, and targeted delivery, the light‐responsive nanomaterials could enable earlier diagnosis, more effective treatments, and real‐time monitoring of therapeutic outcomes. As research progresses, photo‐energy conversion‐based technologies may transform these diseases from being difficult‐to‐manage conditions into ones that are treatable with highly tailored, minimally invasive approaches, marking a significant advancement in the field of nanomedicine.

## Conflict of Interest

The authors declare no conflict of interest.
